# Ba–Ni–Ge Clathrate Transformation Maximizes Active Site Utilization of Nickel for Enhanced Oxygen Evolution Performance

**DOI:** 10.1002/anie.202424743

**Published:** 2025-05-05

**Authors:** Ziliang Chen, Hongyuan Yang, J. Niklas Hausmann, Stefan Mebs, Viktor Hlukhyy, Holger Dau, Matthias Driess, Prashanth W. Menezes

**Affiliations:** ^1^ Department of Materials Chemistry for Catalysis Helmholtz‐Zentrum Berlin für Materialien und Energie Albert‐Einstein‐Str. 15 12489 Berlin Germany; ^2^ Department of Chemistry: Metalorganics and Inorganic Materials Technical University of Berlin Straße des 17 Juni 135. Sekr. C2 10623 Berlin Germany; ^3^ Department Chemie Technische Universität München Lichtenbergstraße 4 85747 Garching Germany; ^4^ Department of Physics Free University of Berlin Arnimallee 14 14195 Berlin Germany; ^5^ Institute of Functional Nano and Soft Materials (FUNSOM) Jiangsu Key Laboratory for Carbon‐based Functional Materials and Devices Soochow University Suzhou 215123 P.R. China

**Keywords:** Bulk intermetallic, Oxygen evolution reaction, Phase reconstruction, Reversible transformation, Ternary clathrate

## Abstract

Discovering novel oxygen evolution reaction (OER) (pre)catalysts with exceptional catalytic activity and long‐term stability is pivotal for advancing decarbonization technologies. In this study, we present the ternary Ba_8_Ni_6_Ge_40_ phase with an open clathrate structure exhibiting remarkable performance in alkaline OER. When integrated into an alkaline water electrolyzer, this clathrate precatalyst achieves high stability under a sustained current density of ∼550 mA cm^−2^ for 10 days. By combining in situ Raman spectroscopy, quasi in situ X‐ray absorption spectroscopy, and (micro)structural characterizations, we elucidate the complete electrochemical transformation of Ba_8_Ni_6_Ge_40_ (~90 weight% leaching) forming ultrathin nanosheets composed of a porous and defective NiOOH nanostructure with maximized accessible active site exposure. Notably, a reversible phase transition mainly between Ni(OH)_2_ and NiOOH has also been established in the electrochemical redox process. Meanwhile, the successful application of the model Ba_8_Ni_6_Ge_40_ precatalyst represents a promising new class of functional inorganic materials for water electrolysis.

## Graphical abstract

For the first time, this study demonstrates a huge advantage of the clathrate Ba–Ni–Ge intermetallic bulk over metallic nickel as a high‐performance alkaline OER precatalyst. It displays remarkable activity and stability under high current densities, facilitated by a thorough reconstruction process that yields a porous and defective NiOOH nanoarchitecture, as comprehensively examined through a series of in situ and ex situ techniques.

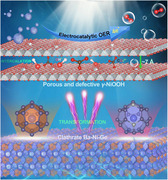

## Introduction

A carbon‐free way to produce hydrogen is electrochemical water splitting, which enables the use of renewable energy sources such as wind and solar power.^[^ ^1^, ^2^, ^3^, ^4^, ^5^ ^]^ However, the critical half‐reaction of water splitting—the anodic oxygen evolution reaction (OER)—is kinetically demanding due to the involvement of four‐proton coupled electron transfers, leading to poor energy conversion efficiency.^[^ ^6^, ^7^, ^8^ ^]^ In order to reduce the kinetic barrier, the discovery of practically relevant, highly active, and long‐term durable electrocatalysts for OER is of great significance.

Over the past decade, a wide variety of transition‐metal (e.g., Ni)‐based oxides, (oxy)hydroxides, chalcogenides, pnictides, and intermetallics have been extensively investigated as OER (pre)catalysts.^[^ ^9^, ^10^, ^11^, ^12^, ^13^, ^14^, ^15^, ^16^, ^17^, ^18^, ^19^, ^20^, ^21^, ^22^, ^23^, ^24^ ^]^ Notably, these materials undergo at least partial transformation into NiOOH phases during catalysis, which then serve as real OER‐active structures.^[^ ^9^, ^10^, ^11^, ^12^, ^13^ ^]^ Based on this observation, Mai et al. further demonstrated that a deeper transformation into NiOOH could foster the formation of more available active sites, resulting in enhanced OER performance.^[^ ^14^ ^]^ Subsequent reports have shown that such transformations, especially when leading to highly porous and defective nanodomains, significantly increase the involvement of active sites.^[^ ^15^, ^16^ ^]^ Therefore, a precatalyst capable of fully reconstructing into porous and defective NiOOH nanodomains is crucial for maximizing active site exposure and OER performance.

Recently, inorganic clathrates have fascinated the scientific community owing to their unique crystal structure, composition, and physicochemical properties.^[^ ^25^, ^26^, ^27^, ^28^, ^29^, ^30^ ^]^ These clathrates exhibit a 3D framework assembled from large polyhedral cages featuring 20–28 vertices, adept at accommodating guest anions or cations. Within these polyhedral cages, host atoms are interconnected by covalent bonds while the guest species reside in off‐center positions of the cage, which may lead to their rattling large‐amplitude anharmonic motions inside the cages and result in extremely low thermal conductivities. Moreover, the inorganic clathrate can be composed of s‐, p‐, d‐, and f‐block elements, endowing them with diverse functionality. Benefiting from these traits, inorganic clathrates have been explored for a variety of applications (e.g., thermoelectrics, superconductors, and Li‐ion batteries), with significant strides made in their development.^[^ ^25^, ^26^, ^27^, ^28^, ^29^, ^30^ ^]^ However, despite these advancements, the potential use of clathrates as electrocatalysts has remained unexplored until now.

As one of the most extensively studied inorganic clathrates, Ba_8_Ni_6_Ge_40_ crystallizes in a cubic structure (*Pm* $\bar{3}$ *n*), featuring a framework comprising two types of cages: smaller tetrakaidecahedra ([Ge_20_Ni_4_]) and larger pentagonal dodecahedra ([Ge_20_]).^[^ ^28^, ^29^, ^30^ ^]^ Each cage accommodates a single guest Ba atom, resulting in the formation of Ba_8_Ni_6_Ge_40_ with a large volume of ∼203 Å^3^ per Ni (only ~8 weight% are Ni). This unique configuration offers several potential advantages over metallic Ni when Ba_8_Ni_6_Ge_40_ is employed as an OER precatalyst: (i) The significant volume disparity between Ba_8_Ni_6_Ge_40_ and NiOOH (>31 Å^3^ per Ni),^[^ ^12^, ^19^ ^]^ along with their distinct local structures, are expected to synergistically drive the formation of a highly porous and electrolyte‐penetrable defective catalyst structure with many edge sites; (ii) a significant amount of Ba and Ge species within the framework are likely to leach, accelerating the reconstruction of the porous active phase during OER; (iii) the intermetallic nature of Ba_8_Ni_6_Ge_40_ facilitates charge transfer, thereby promoting phase conversion. Having said this, the following research questions arose: (i) Can Ba_8_Ni_6_Ge_40_ be transformed into a Ni‐based active phase during the electrochemical alkaline OER process? (ii) What are the structural characteristics of the resulting active phase? (iii) Despite the low Ni content, does the reconstructed active phase demonstrate high OER activity and durability? (iv) Could this transformation provide new insights into the Ni‐based OER mechanism concerning structure‐activity relationships?

In our study, we investigated Ba_8_Ni_6_Ge_40_ precatalyst deposited on both fluorine‐doped tin oxide (Ba_8_Ni_6_Ge_40_/FTO) and nickel foam (Ba_8_Ni_6_Ge_40_/NF). After electrochemical activation, both of them revealed significantly lower overpotential compared to previously reported intermetallics and Ni‐based electrocatalysts.^[^ ^16^, ^31^, ^32^, ^33^, ^34^ ^]^ In particular, the mass activity based on Ni content for Ba_8_Ni_6_Ge_40_ surpassed that of pristine Ni by nearly an order of magnitude across a wide potential range under the same alkaline OER conditions. Furthermore, the NF‐supported Ba_8_Ni_6_Ge_40_ precatalyst exhibited remarkable stability. When used as the cathode and anode of an assembled alkaline water electrolyzer, a pronounced current density of around 550 mA cm^−2^ can be maintained over a duration of 10 days. Extensive analyses employing in situ Raman spectroscopy, quasi in situ X‐ray absorption spectroscopy (XAS), as well as ex situ (micro)structure characterizations, uncovered that the large particles of Ba_8_Ni_6_Ge_40_ underwent complete electroconversion into ultrathin nanosheet‐composed, porous, and defective NiOOH nanocrystals under alkaline OER catalysis. This transformation is attributed to the unique cage‐like structure of Ba_8_Ni_6_Ge_40_ and the synergistic effects of its multiple components, resulting in the formation of a highly active structure. To the best of our knowledge, this is also the first report that fast and complete reconstruction can be achieved for bulk intermetallics. Moreover, our investigation confirmed a reversible phase transition between Ni(OH)_2_ and NiOOH during the electrochemical redox process, as evidenced by the reversible electrode color variation between light grey and dark grey. This reversible transition is crucial for maintaining high catalytic activity over prolonged periods. In this contribution, we successfully utilized Ba₈Ni₆Ge₄₀ for the first time as an efficient alkaline OER precatalyst. Its remarkable performance highlights the immense potential of inorganic clathrates, a previously overlooked material class, for transformative applications in water electrolysis.

## Results and Discussion

### Structural Characterizations on the Fresh Ba_8_Ni_6_Ge_40_

**Figure 1:**
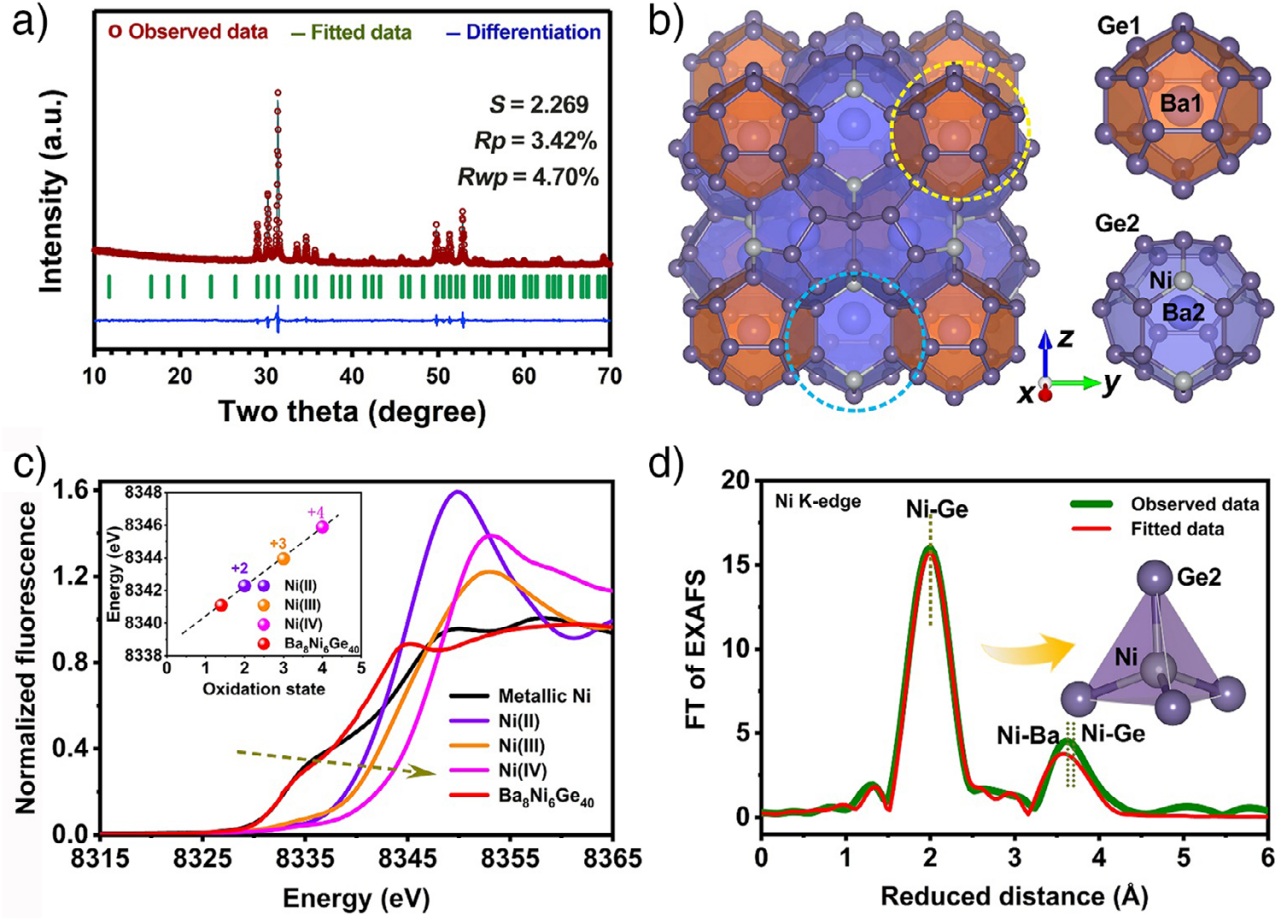
a) Rietveld refinement of PXRD pattern of the Ba_8_Ni_6_Ge_40_ phase, where *S*, *R*_p_, and *R*_wp_ represent the goodness‐of‐fit indicator, unweighted profile parameter, and weighted profile parameter, respectively; b) schematic illustration of the crystal structure of the Ba_8_Ni_6_Ge_40_ clathrate with two types of cages; c) XANES spectra of Ni K‐edge of Ba_8_Ni_6_Ge_40_, together with standard samples including Ni foil (metallic Ni^0^), Ni^II^O, LiNi^III^O_2_, and K_2_Ni^IV^(H_2_IO_6_)_2_. To quantify differences in the edge positions, the K‐edge energy was calculated using the integral method.^[^ ^35^ ^]^ The resulting values were plotted in the inset of (c). The three reference compounds (i.e., Ni^II^O, LiNi^III^O_2_, and K_2_Ni^IV^(H_2_IO_6_)_2_) were employed for linear regression (black broken line in the inset of (c)) to correlate the edge energies to oxidation states. The energy axis was calibrated by measurement of a Ni metal foil in transmission mode for the powder samples and shifting the first inflection point of the XANES rise to 8333 eV. d) Fourier‐transformed (FT) Ni K‐edge EXAFS spectra of Ba_8_Ni_6_Ge_40_. For precise distances and further parameters determined by EXAFS simulations (fits), see Table S2. The indicated reduced distances are about 0.3 Å smaller than the precise distances determined by EXAFS fits.

Rietveld refinement of the powder X‐ray diffraction (PXRD) pattern of the as‐prepared sample (Figure 1a and Table S1) shows that we successfully synthesized pure Ba_8_Ni_6_Ge_40_, which crystallized in the cubic space group *Pm* $\bar{3}$ *n*, with a lattice parameter of *a* = 10.6754(5) Å. The clathrate structure of Ba_8_Ni_6_Ge_40_ contains two types of cages, constructed by 20‐ and 24‐atom polyhedra, respectively (Figure 1b). Notably, the central position within these cages is occupied by Ba atoms, while the framework is formed by Ni and/or Ge atoms.^[^ ^28^ ^]^ The slow rise of the Ni K‐edge X‐ray absorption near‐edge structure (XANES) of Ba_8_Ni_6_Ge_40_ strongly indicates a metallic Ni‐like character (Figure 1c). A slight surface passivation could take place, which was commonly observed in intermetallics.^[^ ^20^, ^21^, ^22^, ^23^ ^]^ Furthermore, extended X‐ray absorption fine structure (EXAFS) analysis of Ni species confirmed the clathrate structure of Ba_8_Ni_6_Ge_40_, with bond distances of 2.29, 3.92, and 3.98 Å for Ni–Ge, Ni–Ba, and Ni–Ge, respectively (Figures 1d, S1, and Table S2).

**Figure 2:**
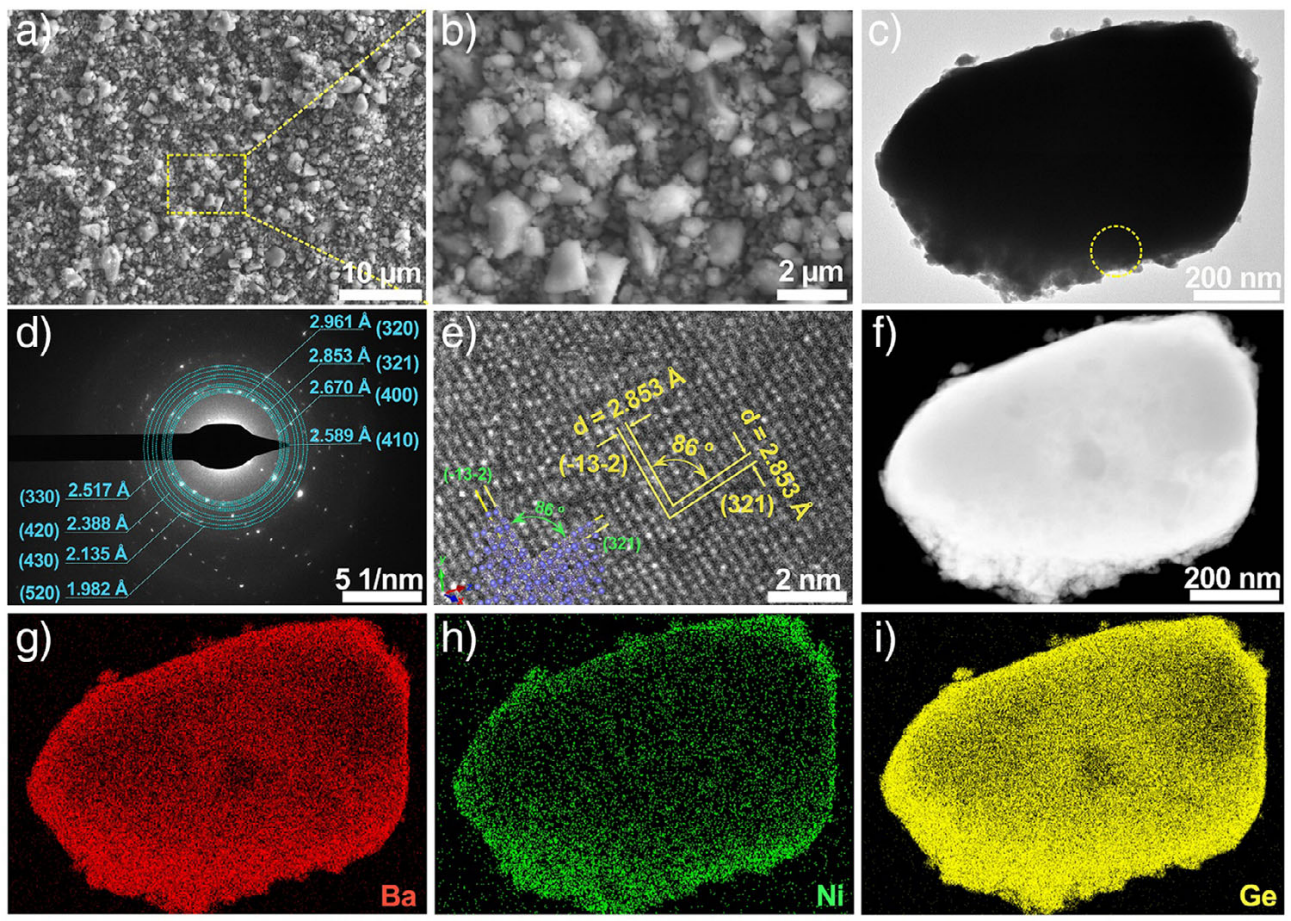
a, b) SEM images of Ba_8_Ni_6_Ge_40_/FTO; c) TEM image of Ba_8_Ni_6_Ge_40_ scratched off from electrode and corresponding d) SAED, e) HRTEM, f) HAADF, and g) Ba, h) Ni, and i) Ge elemental mappings.

To examine the electrocatalytic properties of Ba_8_Ni_6_Ge_40_, it was first deposited on FTO via electrophoretic deposition (EPD) (Ba_8_Ni_6_Ge_40_/FTO) and utilized directly as the working electrode. To demonstrate that the EPD process does not affect the clathrate phase, the PXRD pattern for Ba_8_Ni_6_Ge_40_/FTO was also examined (Figure S2), clearly showing the diffraction peaks for Ba_8_Ni_6_Ge_40_ and SnO_2_ substrate. Next, the morphology of bulk Ba_8_Ni_6_Ge_40_ powder deposited on FTO substrate was analyzed by scanning electron microscopy (SEM). Numerous particles were observed to be tightly coated on the surface of FTO (Figure 2a), with the particles exhibiting a pseudosphere‐like morphology mainly distributed in the range from 0.2 to 3 µm (Figure 2b). Consistent with the SEM results, the transmission electron microscopy (TEM) image of the powder sample scratched off from the FTO also revealed the pseudosphere‐like morphology (Figure 2c). The diffraction rings in the selected area electron diffraction (SAED) pattern could be well indexed as the lattice planes of the Ba_8_Ni_6_Ge_40_ phase (Figure 2d). Furthermore, a high‐resolution TEM (HRTEM) image demonstrated the crystal structure of Ba_8_Ni_6_Ge_40_ with an included angle of around 86° between (3 2 1) and (−1 3 −2) facets (Figure 2e). The high‐angle annular dark field‐scanning TEM (HAADF‐STEM) image (Figure 2f) and corresponding elemental mappings displayed the homogeneous overlap of Ba (Figure 2g), Ni (Figure 2h), and Ge species (Figure 2i). Additionally, the inductively coupled plasma (ICP) also revealed the atomic ratio of 1.6:1:8.4 among Ba, Ni, and Ge, which closely approximated the theoretical ratio. Therefore, these characterization results strongly validated the well‐preserved phase composition, structure, and morphology after EPD.

### OER Electrochemistry on FTO

**Figure 3:**
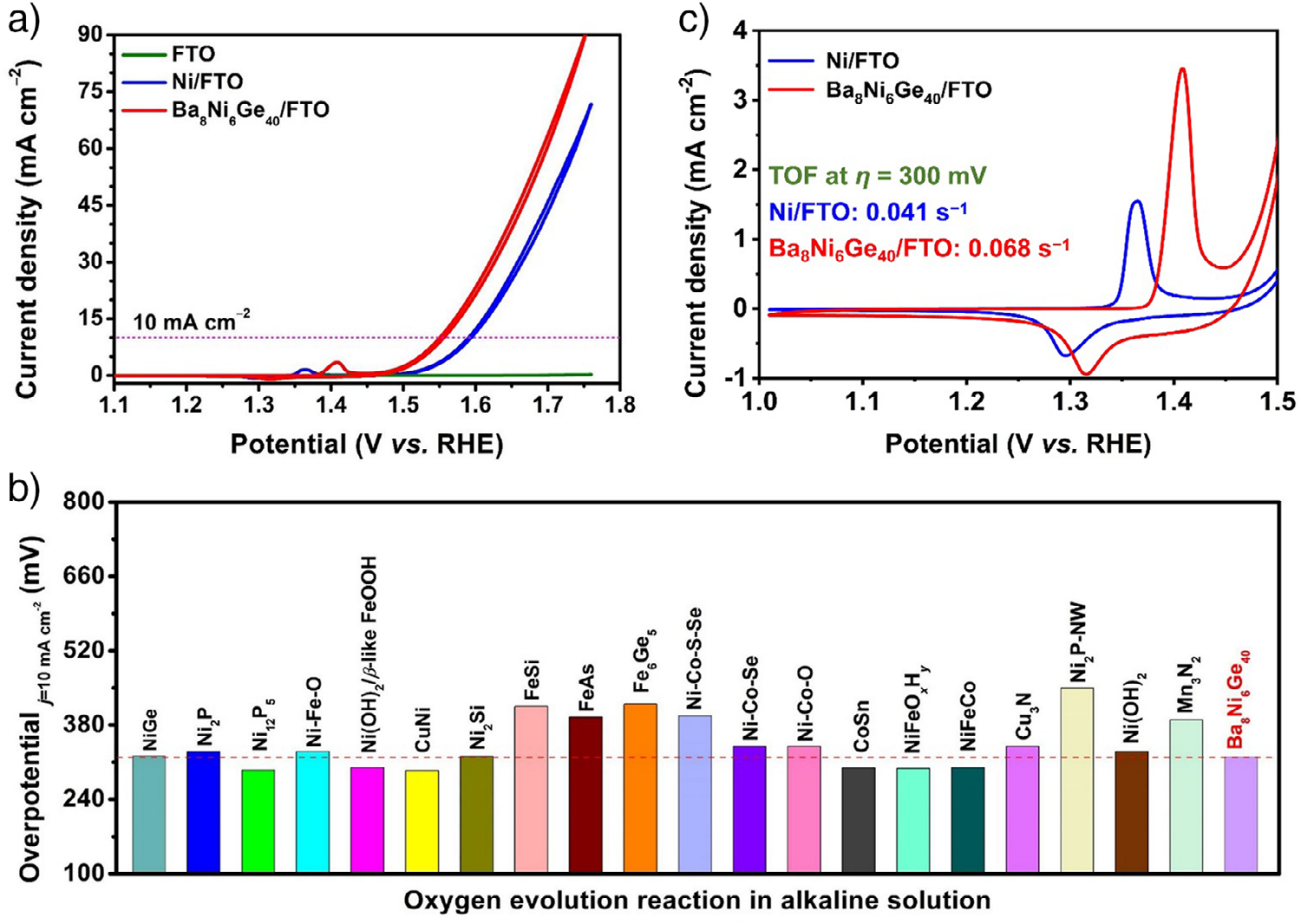
a) CV curves of Ni/FTO and Ba_8_Ni_6_Ge_40_/FTO (5 mV s^−1^; 1 mg cm^−2^ catalyst loading and for Ba_8_Ni_6_Ge_40_/FTO, only 80 µg cm^−2^ Ni loading. b) Comparison of OER overpotential at 10 mA cm^−2^ in 1.0 M KOH between Ba_8_Ni_6_Ge_40_/FTO and recently reported FTO‐supported intermetallics and Ni‐based electrocatalysts (the mean value of the overpotential for Ba_8_Ni_6_Ge_40_/FTO was adopted herein). c) Redox peaks from CV curves in (a) for Ni/FTO and Ba_8_Ni_6_Ge_40_/FTO and their respective redox‐normalized TOF values (estimated based on integrating reduction peaks; details in Figure S7). Note that the pre‐activation was performed before all the crucial electrochemical tests, including CV and Tafel measurements.

The electrocatalytic properties of Ba_8_Ni_6_Ge_40_/FTO were evaluated in a three‐electrode setup in 1 M KOH. Figure 3a shows the cyclic voltammetry (CV) data. To better demonstrate the superior activity, the CV curve of metallic Ni particles deposited on FTO with the same mass loading (Figures S3 and S4) was also tested, together with the blank FTO for comparison. Remarkably, the electrochemically activated Ba_8_Ni_6_Ge_40_/FTO only required an overpotential of 321 ± 2 mV at the current density of 10 mA cm^−2^, which was not only much lower than that of Ni/FTO sample but also superior/comparable to those of state‐of‐the‐art intermetallics and Ni‐based catalysts supported on FTO (Figures 3b, S5, and Table S3). Notably, steady‐state Tafel slopes (Figure S6) for Ni/FTO and Ba_8_Ni_6_Ge_40_/FTO after activation showed similar values, implying similar reaction kinetics and kinds of active sites. On the other hand, in order to reflect the intrinsic catalytic activity, the turnover frequency (TOF) values were estimated based on the catalyst's redox activity for metallic Ni/FTO and Ba_8_Ni_6_Ge_40_/FTO are presented in Figures 3c and S7. Notably, Ba_8_Ni_6_Ge_40_/FTO has around 25 times higher redox activity per loaded nickel site than the Ni/FTO reference sample. Thus, its redox activity is still larger even though its nickel loading is around 12.4 times smaller than that of Ni/FTO. In addition, the redox‐normalized TOF value of Ni/FTO was close to that of Ba_8_Ni_6_Ge_40_/FTO. At the same time, the current densities of the OER CV curves for these two samples were normalized against their respective number of redox‐active Ni that were involved into the catalysis per cm^2^ of the geometrical sample area (*n*). As shown in Figure S8, Ni/FTO and Ba_8_Ni_6_Ge_40_/FTO were again verified to possess similar intrinsic activity. Interestingly, Ba_8_Ni_6_Ge_40_/FTO exhibited significantly higher mass‐normalized activity than that of Ni/FTO. Specifically, at 0.3 V overpotential (1.53 V vs. RHE), Ba_8_Ni_6_Ge_40_/FTO can afford ∼50 mA mg^−1^ current density, ∼50 times higher than that of Ni/FTO (Figure S9). These results suggest that the clathrate precatalyst does not lead to different kinds of active sites but to a substantially higher active site availability. It is also worth noting that Ba_8_Ni_6_Ge_40_ sample exhibited a more anodic shift of Ni^II/III^ redox peaks compared with that of the bare Ni, which could be ascribed to more incorporation of Fe from the KOH electrolyte. It has been widely known that more Fe doping can induce a positive shift of the Ni^II/III^ redox peaks for Ni‐based (oxy)hydroxides. This is probably due to the stabilization of low‐valent Ni^II^ by the incorporated Fe. Thus, the conversion between Ni^II^ and Ni^III^ takes place at more positive potentials.^[^ ^36^, ^37^, ^38^ ^]^ Moreover, the charge transfer resistance derived from electrochemical impedance spectroscopy (EIS) of the activated Ba_8_Ni_6_Ge_40_/FTO was slightly smaller than the activated Ni/FTO (Figure S10 and Table S4).

### Determination of the Reconstructed Structure

**Figure 4:**
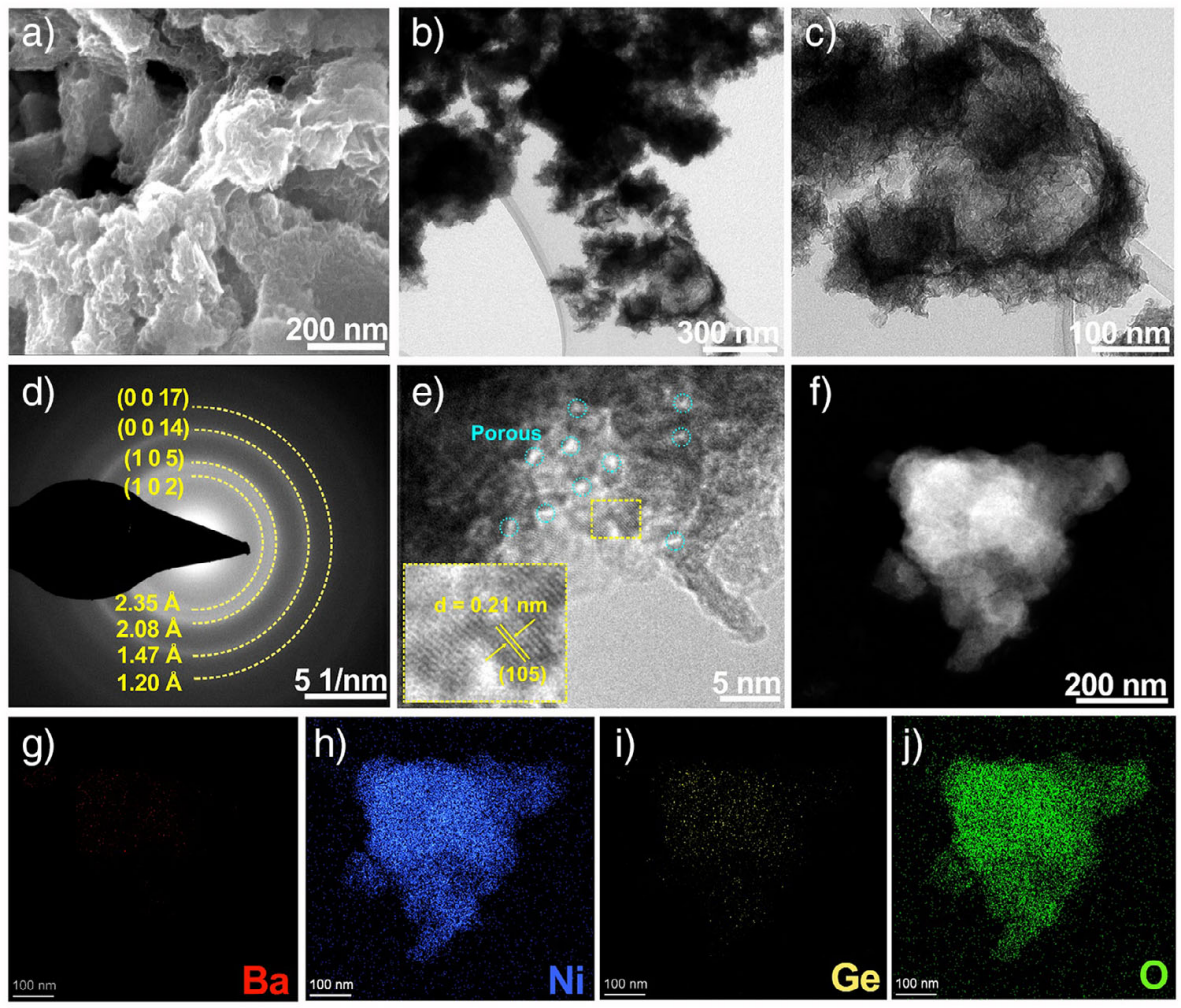
a) SEM image of Ba_8_Ni_6_Ge_40_/FTO after OER cycle (CA, 24 h), b) and c) TEM images, d) SAED pattern, e) HRTEM, f) STEM‐HAADF and corresponding elemental mappings of g) Ba, h) Ni, i) Ge and j) O for Ba_8_Ni_6_Ge_40_ after OER cycle (CA, 24 h, see Figure S17 for the corresponding EDX spectrum).

To understand the fundamental factors driving the higher OER activity, a series of characterizations, including PXRD, X‐ray photoelectron spectroscopy (XPS), SEM, TEM, and energy dispersive X‐ray (EDX), were performed for a post‐OER Ba_8_Ni_6_Ge_40_/FTO electrode (treated using 24 h chronoamperometry, CA, at 1.55 V vs. RHE). Figure S11 showed the CV curves of Ba_8_Ni_6_Ge_40_/FTO against cycle numbers, from which an activation process was observed. Besides, during the OER CA, the activity of the FTO‐supported Ba_8_Ni_6_Ge_40_ precatalyst was stabilized within 24 h (Figure S12). The post‐CA PXRD pattern for the Ba_8_Ni_6_Ge_40_/FTO displayed only diffraction peaks attributable to the FTO substrate, implying the complete reconstruction of the Ba_8_Ni_6_Ge_40_/precatalyst (Figure S13). The grazing incidence X‐ray diffraction (GIXRD) measurement was further conducted for the same sample. As shown in Figure S14, no clear XRD peaks could be assigned to a reconstructed phase. This was probably due to the ultrasmall crystallite size of the in situ reconstructed species (as indicated by the below TEM characterizations), the low content of the ultrathin catalyst film, and the strong intensity of the diffraction peaks of the FTO substrate. High‐resolution XPS spectra of Ni 2p indicated the conversion of all Ni species to Ni^III^ (Figure S15a), suggesting the possible formation of NiOOH. Corresponding high‐resolution XPS spectra of Ba 3d and Ge 3d also displayed an oxidative leaching trend, consistent with their respective Pourbaix diagrams (Figure S15b,c).^[^ ^16^, ^21^, ^39^, ^40^, ^41^, ^42^, ^43^, ^44^, ^45^ ^]^ Moreover, SEM and TEM (recorded from different angles) images illustrated the transformation from pseudosphere‐like morphology to a flower‐like architecture assembled by nanosheets (Figures 4a–c and S16). The diffraction rings recorded from the SAED pattern could be well indexed as (1 0 2), (1 0 5), (0 0 14), and (0 0 17) facets of the nanocrystalline γ‐NiOOH phase (PDF #06‐0075) (Figure 4d), respectively, and no diffraction rings of the clathrate phase could be found, signifying the complete reconstruction after OER CA. As shown in the HRTEM image of the post‐OER Ba_8_Ni_6_Ge_40_, the interplanar spacing of the lattice fringes within the selected region was 0.21 nm, corresponding to the (1 0 5) plane of γ‐NiOOH (PDF #06‐0075), confirming again the reconstructed NiOOH nanocrystals (Figure 4e). Besides, this HRTEM image depicted a number of γ‐NiOOH nanocrystals with an average size of around 5 nm, which were interconnected to form porous and defective nanosheets (Figure 4e). HAADF‐STEM images and the related elemental mapping showed an intensive and homogeneous distribution of Ni and O species within the nanoarchitecture, along with a dissolution of almost all Ba and Ge (Figure 4f–j). Consistently, EDX analysis indicated an atomic ratio of Ni:Ba:Ge as 1:0.02:0.06 post‐OER CA, consistent with ICP results (1:0.12:0.15), suggesting facile leaching of Ba and Ge from the clathrate structure, contributing to electrochemical reconstruction (Figure S17). Compared to Ni/FTO, Ba_8_Ni_6_Ge_40_/FTO exhibited enhanced CA stability (Figure S18). Further analysis revealed only a very limited transformation for Ni/FTO after OER CA, with an amorphous phase shell (∼5 nm thickness) surrounding the metallic Ni core (Figures S3, S4, S19, and S20). Therefore, the incorporation of the clathrate structure, along with the presence of Ba and Ni facilitated rapid electrochemical phase reconstruction of the bulk intermetallic into defective and porous Ni‐based (oxy)hydroxides, which further promoted the penetration of KOH electrolyte and, accordingly, the impurity Fe doping.^[^ ^39^, ^46^ ^]^ Note that the elemental analysis revealed an atomic ratio of Fe to Ni species of approximately 0.013:1 in the reconstructed phase after CA, attributed to the presence of Fe species from the electrolyte. Despite the relatively low quantity, the presence of Fe contributed to enhancing OER activity (Figure S21).^[^ ^39^, ^46^, ^47^, ^48^ ^]^

**Figure 5:**
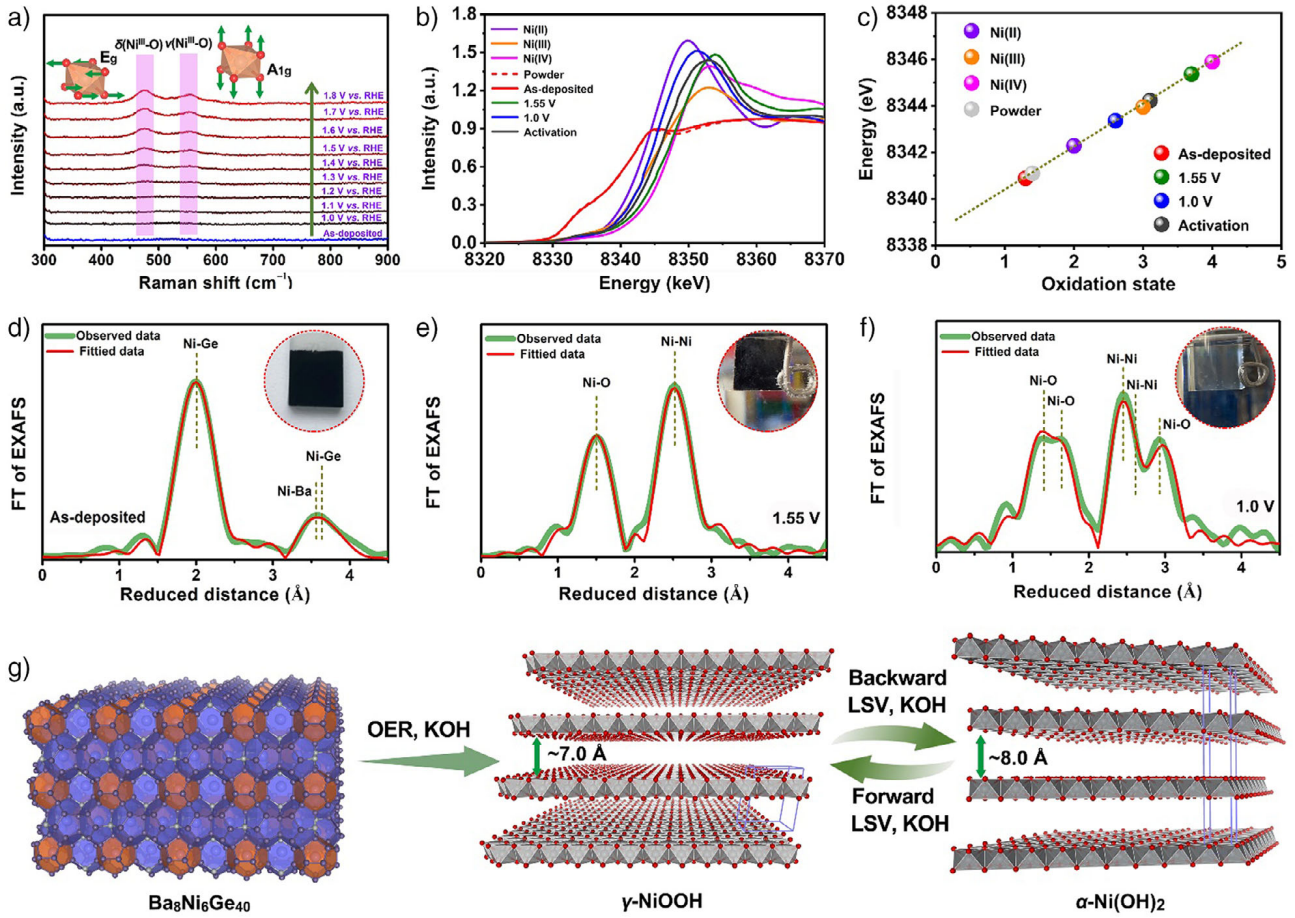
a) In situ Raman spectra of Ba_8_Ni_6_Ge_40_/FTO collected at different applied voltages in alkaline OER process, as well as the ex situ one on the fresh as‐deposited Ba_8_Ni_6_Ge_40_/FTO; b) XANES spectra of Ni *K*‐edge in Ba_8_Ni_6_Ge_40_/FTO electrode, Ba_8_Ni_6_Ge_40_/FTO electrode after OER CA at 1.55 V versus RHE, Ba_8_Ni_6_Ge_40_/FTO electrode after OER CA at 1.55 V versus RHE followed by an OER CA at 1.0 V versus RHE, as well as Ba_8_Ni_6_Ge_40_/FTO electrode after the 10th CV cycle, and c) corresponding Ni oxidation state determined by analysis of XANES spectra. The Ni *K*‐edge XANES spectra of the standard reference samples, including Ni foil (metallic Ni^0^), Ni^II^O, LiNi^III^O_2_, and K_2_Ni^IV^(H_2_IO_6_)_2_, were also included. Fitted FT‐EXAFS spectra of Ni *K*‐edge in d) Ba_8_Ni_6_Ge_40_/FTO electrode, e) Ba_8_Ni_6_Ge_40_/FTO electrode after OER CA at 1.55 V versus RHE, f) Ba_8_Ni_6_Ge_40_/FTO electrode after OER CA at 1.55 V versus RHE followed by an OER CA at 1.0 V versus RHE. g) Schematical illustration for the structural evolution of Ba_8_Ni_6_Ge_40_ during alkaline OER. Inset in d–f: digital images showing electrode color at the corresponding reaction states; at 1.0 V versus RHE (inset in f), the electrode film is transparent. In d–f, the indicated reduced distances are about 0.3 Å smaller than the precise distances determined by EXAFS fits (see Table S5).

In order to gain a deeper understanding of the reconstruction mechanism, the real‐time in situ Raman spectra of the Ba_8_Ni_6_Ge_40_ film/FTO under different applied voltages, together with the ex situ one of the fresh electrodes, were collected (Figure 5a). As compared to the as‐deposited electrode with no detectable Raman signals, two characteristic bands at around 473 and 556 cm^−1^ were identified, which could be assigned to the Ni─O vibration in γ‐NiOOH.^[^ ^16^, ^17^, ^39^ ^]^ Specifically, the former corresponds to the depolarized *E*_g_ mode (bending), while the latter is associated with the polarized *A*_1g_ mode (stretching). As shown in Figure 5a inset, the oxygen atoms vibrated along the plane in the *E*_g_ mode, whereas they vibrated perpendicular to the plane in the *A*_1g_ mode. This finding clearly reaffirmed the transformation of Ba_8_Ni_6_Ge_40_ to γ‐NiOOH after OER. To gain more insights, quasi in situ XAS was performed for the Ba_8_Ni_6_Ge_40_ film by freeze quenching the samples in liquid nitrogen (see Supporting Information for details). As seen in Figures 5b–d, S22 and Table S5, both the XANES and EXAFS curves for the as‐deposited Ba_8_Ni_6_Ge_40_ film (on FTO) overlap well with those for the Ba_8_Ni_6_Ge_40_ powder, indicating that EPD did not alter the clathrate structure. Subsequently, quasi in situ XAS was performed for the Ba_8_Ni_6_Ge_40_ film after 24 h of OER CA at 1.55 V (vs. RHE; the as‐obtained sample was denoted as “1.55 V”). As seen in Figure 5b,c, the valence state for Ni was increased from its initial metallic state to +3.7 during OER CA at 1.55 V. EXAFS simulation illustrated the complete formation of NiOOH (Figures 5e, S22, and Table S5), in accordance with the aforementioned HRTEM, XPS, and in situ Raman results. Following a further 24 h of CA at the potential of 1.0 V versus RHE to reduce the sample to 1.55 V (Figure S23, the as‐obtained sample was denoted as “1.0 V”), the valence state for Ni was decreased from the initial +3.7 to +2.6 (Figure 5b,c). Analysis from the EXAFS fitting (Figures 5f, S22, and Table S5) revealed the major existence of Ni(OH)_2_, indicating that most of the reconstructed NiOOH phase was reduced into Ni(OH)_2_. Moreover, we specially conducted the CV cycle for this sample within a more cathodic potential window to detect the Ni^II^ reduction peak. As shown in Figure S24, a notable Ni^II^ reduction peak emerged close to the HER region, in agreement with the previous reports, as well as the Pourbaix diagram of Ni.^[^ ^49^, ^50^ ^]^ This finding further validated the domination of Ni(OH)_2_ phase. The OER activity of the same sample was also examined, which was still good (Figure S25), probably because Ni(OH)_2_ rapidly transformed into active NiOOH at OER potentials.

Notably, in many cases, the precise determination and deconvolution of the exact Ni^II^‐based phases transformed from the Ni^III^‐based ones are lacking based on the in situ XAS measurements, likely due to the insufficient accumulation of such Ni^II^‐based phases.^[^ ^37^, ^51^, ^52^, ^53^ ^]^ Specifically, at the potentials below the redox Ni^II/III^ transition, the Ni^II^‐based phases should, in principle, form.^[^ ^51^, ^54^ ^]^ Whereas (i) the evolved Ni^II^‐based species could typically be present in low amounts and confined at the (near) surface region, as only small portions of Ni^III^‐based species can be reduced during short‐term electrochemical treatments in real‐time in situ measurements.^[^ ^55^ ^]^ (ii) At the same time, the Ni^II^‐based species concentrating on the (near) surface area are barely detected by the bulk‐sensitive XAS.^[^ ^56^ ^]^ (iii) Literature reports involving reversible Ni^II/III^‐based phases well defined by in situ XAS mostly utilize (oxy)hydroxides as the studied catalytic models.^[^ ^54^, ^57^, ^58^ ^]^ In contrast, deep transformation among Ni^II/III^‐based phases is seldom observed on the intermetallic precatalysts, as these precatalysts generally exhibit a limited reconstruction degree during the alkaline OER.^[^ ^15^, ^51^ ^]^ In this context, our bulk Ba_8_Ni_6_Ge_40_ precatalyst successfully undergoes a complete transformation into porous and defective Ni^III^OOH under alkaline OER scenarios. Subsequently, it is reduced under 1.0 V versus RHE (far below the potentials of the Ni^II/III^ redox peaks) for a sufficient treatment period. Consequently, the deep reversible conversion of the reconstructed Ni^III^OOH into sufficient XAS‐detectable Ni^II^(OH)_2_ is realized.

To demonstrate the fast reconstruction ability of Ba_8_Ni_6_Ge_40_ during OER, quasi in situ XAS spectra for the electrode after activation by 10 OER CV (denoted as “activation”) were obtained, which were found to be similar to that of the sample at 1.0 V. Specifically, the valence state for Ni was increased from +1.3 to +3.1 (Figure 5b,c). EXAFS fitting (Figures S22 and S26, Table S5) indicated a composition primarily comprising Ni(OH)_2_ and NiOOH phases, affirming robust reconstruction ability, which was further supported by the PXRD pattern for Ba_8_Ni_6_Ge_40_/FTO after CV activation (Figure S27). Moreover, compared with the fresh samples, Ba and Ge were severely dissolved from Ba_8_Ni_6_Ge_40_ precatalyst under different stages of electrochemistry, as shown in their respective raw spectra of the samples at 1.55 V, 1.0 V, and activation (Figure S28). Furthermore, regarding the residual Ba and Ge within these three quasi in situ samples, the associated XANES spectra revealed an apparently increased oxidation state compared to those of the as‐prepared and as‐deposited Ba_8_Ni_6_Ge_40_ (Figures S29a, S30a). This illustrated that Ba and Ge of the fresh Ba_8_Ni_6_Ge_40_ were oxidized into soluble oxyanions and subsequently dissolved into the electrolyte during electrochemistry. The comparison between Ba and Ge EXAFS data of the three quasi in situ samples with those of the as‐prepared and as‐deposited Ba_8_Ni_6_Ge_40_ uncovered that the coordination environments were severely changed, which could be induced by oxidative leaching (Figures S29b–d, S30b–d, and Table S6). These data supplementally evidenced that the reconstruction of Ba_8_Ni_6_Ge_40_ precatalyst into Ni(OH)_2_/NiOOH was accompanied by the oxidation and dissolution of Ba and Ge during OER electrocatalysis. Interestingly, noticeable changes in color occurred at various OER stages (inset in Figure 5d–f). Particularly, the sample 1.55 V exhibited a dark grey color (inset of Figure 5e), which was strikingly turned into transparent after the follow‐up 24 h CA at a 1.0 V versus RHE (1.0 V, inset of Figure 5f). Subsequently, the sample 1.0 V was further subject to CV cycle. With the potentials proceeding into the substantial OER region, the color of the deposited catalyst film gradually changed into dark grey and became transparent when the applied potential reverted and recovered (see Video, Supporting Information), probably aligning with the reversible shift between Ni(OH)_2_ and NiOOH phases. As suggested by the SAED and HRTEM analysis on the post‐CA Ba_8_Ni_6_Ge_40_ (the sample 1.55 V) in Figure 4d,e, as well as its high Ni oxidation state of +3.7 in Figure 5b,c, the NiOOH reconstructed from Ba_8_Ni_6_Ge_40_ under 1.55 V versus RHE was in the form of *γ* phase, known for a higher oxidation state (around +3.6) and larger interlayer distance facilitating ion intercalation (e.g., CO_3_^2−^ and K^+^), correlating with high activity.^[^ ^16^, ^17^, ^39^ ^]^ Remarkably, according to the Bode diagram (Figure S31),^[^ ^57^, ^59^, ^60^ ^]^ the *γ*‐NiOOH can be reduced into both α‐Ni(OH)_2_ and β‐Ni(OH)_2_. In addition, the transformation between α‐Ni(OH)_2_ and β‐Ni(OH)_2_ occurs quickly under ambient conditions, with precise identification of local structural variances between these phases challenging. Thus, we suggest that the phase transformation from *γ*‐NiOOH to α‐/β‐Ni(OH)_2_ possibly occurred concurrently during CV cycles. Nevertheless, this study represents the first demonstration of the reversible transformation between *γ*‐NiOOH and α‐/β‐Ni(OH)_2_ during the OER process, substantiated through both micro‐structural characterization and macro‐color visualization for intermetallic precatalysts. In addition, exploring the corresponding phases and their mutual transformation is highly significant for understanding the real active species (sites). Based on the above analyses, the phase reconstruction process is schematically depicted in Figure 5g.

### OER Performances Measured on NF

**Figure 6:**
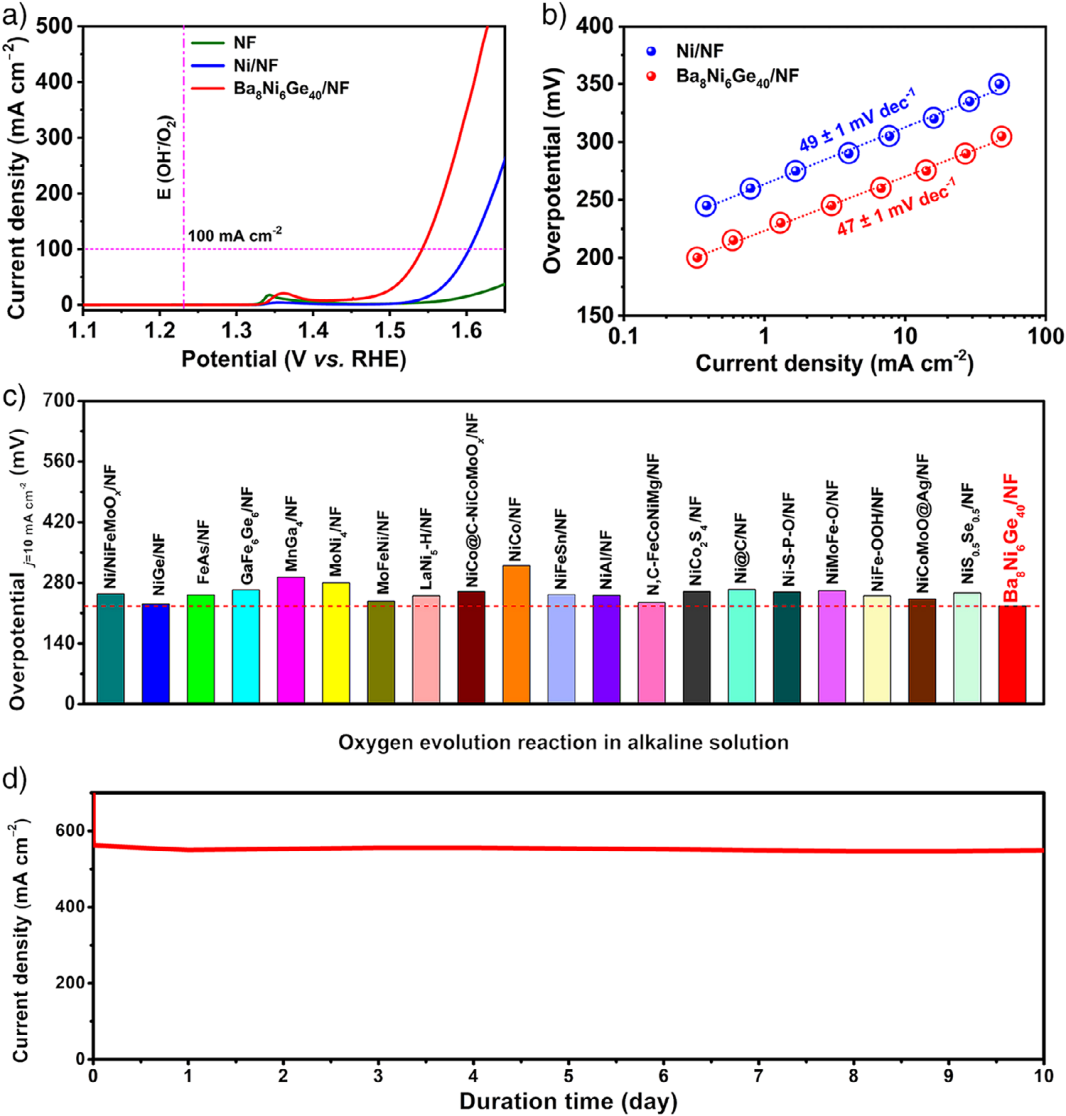
a) LSV curves of Ni/NF, Ba_8_Ni_6_Ge_40_/NF, and blank NF (2 mg cm^−2^ catalyst loading, and for Ba_8_Ni_6_Ge_40_/NF, only 160 µg cm^−2^ Ni loading); b) Tafel slopes of Ni/NF and Ba_8_Ni_6_Ge_40_/NF. Note that the LSV and Tafel measurements were conducted after activation. c) Comparison of OER overpotential at 10 mA cm^−2^ in 1.0 M KOH between Ba_8_Ni_6_Ge_40_/NF and recently reported NF‐supported intermetallics and Ni‐based electrocatalysts (the mean value of the overpotential for Ba_8_Ni_6_Ge_40_/NF was adopted herein), d) stability (CA) of an assembled electrolyzer equipped with the NF‐supported Ba_8_Ni_6_Ge_40_ precatalyst as both cathode and anode at a fixed cell voltage of 2.25 V (at the OER potential of ∼1.718 V vs. RHE and HER potential of ∼0.532 V vs. RHE, respectively).

Inspired by the above results, Ba_8_Ni_6_Ge_40_ precatalyst was further deposited on the low‐cost, high surface area, and highly conductive nickel foam (Ba_8_Ni_6_Ge_40_/NF) to explore its potential application as an OER precatalyst under large current density (Figures S32 and S33). The OER activity of Ba_8_Ni_6_Ge_40_/NF was initially examined by linear sweep voltammetry (LSV) scans, alongside blank NF, Ni/NF, and commercial RuO_2_/NF and IrO_2_/NF for comparison in identical conditions (Figures 6a, S34, and S35). Remarkably, Ba_8_Ni_6_Ge_40_/NF only required overpotentials of 227 ± 2 and 319 ± 2 mV at current densities of 10 and 100 mA cm^−2^, respectively, significantly lower than those of Ni/NF and RuO_2_/NF. Moreover, the Ba_8_Ni_6_Ge_40_/NF also showed a Tafel slope as low as 47 ± 1 mV dec^−1^, which was still similar to that of Ni/NF, in agreement with the trend measured on FTO (Figure 6b). Its impressive activity positions Ba_8_Ni_6_Ge_40_/NF among the top‐performing NF‐supported intermetallics and Ni‐based electrocatalysts reported to date (Figure 6c and Table S7). Moreover, EIS data demonstrated a better charge transfer capability of NF‐supported Ba_8_Ni_6_Ge_40_ compared to metallic Ni (Figure S36 and Table S4) during OER catalysis. To further validate the performance at large current densities, the CA measurement was conducted over an assembled membrane‐free two‐electrode electrolyzer containing the NF‐supported Ba_8_Ni_6_Ge_40_ precatalyst as both cathode and anode at the industrial current density range (>500 mA cm^−2^) under room temperature. This configuration enables a simple and effective preliminary evaluation of its application potential in alkaline water electrolyzers, as widely utilized in previous literature.^[^ ^61^, ^62^, ^63^ ^]^ As expected, the CA curve delivered a stable output of around 550 mA cm^−2^ for over 10 days (Figure 6d) at a cell voltage of 2.25 V (corresponding to an OER potential of ∼1.718 V vs. RHE), substantiating the remarkable catalytic stability of Ba_8_Ni_6_Ge_40_/NF at the large current density. Note that the Ba_8_Ni_6_Ge_40_/NF displays a moderate alkaline hydrogen evolution reaction (HER) (Figure S37); thus, a relatively high cell voltage (2.25 V) was caused by the assembled electrolyzer. A more efficient HER cathode potentially diminishes the cell voltage when integrated with the Ba_8_Ni_6_Ge_40_/NF anode, which will be investigated in future studies. The post‐CA Ba_8_Ni_6_Ge_40_/NF used as the anode was further characterized, revealing phase structures and morphologies akin to those of Ba_8_Ni_6_Ge_40_/FTO after OER CA (Figures S38, S39). Moreover, the above‐assembled electrolyzer can further deliver a significantly high current density of ∼880 mA cm^−2^ at a cell voltage of 2.2 V at an industrially relevant operation temperature (80 °C) (Figure S40), suggesting the promising application potentials of the Ba_8_Ni_6_Ge_40_ precatalyst. It is worth noting that Ba_8_Ni_6_Ge_40_ belongs to the type I clathrates, whose framework typically consists of Group 14 elements, especially Si and Ge, together with some particular substitutional transition metals (TMs, such as Ni in our case).^[^ ^28^, ^64^, ^65^, ^66^ ^]^ Given the abundant availability and low cost of Si in the Earth's crust, as well as its high susceptibility to dissolution in aqueous electrolytes under alkaline OER,^[^ ^23^, ^50^, ^51^, ^67^ ^]^ the Si‐based type I clathrates containing OER‐active TMs potentially act as the inexpensive and efficient precatalysts for alkaline OER. They are expected to resolve the economic concerns of the current Ge‐based counterparts caused by the high price of Ge metal and will be explored in our future work.

## Conclusion

We have systematically addressed and resolved the research questions (i)–(iv) outlined in the introduction. Concerning questions (i) and (ii), our post‐OER analysis, combining in situ Raman, quasi in situ XAS, and ex situ (micro)structure characterization, demonstrated a complete transformation from the clathrate Ba_8_Ni_6_Ge_40_ precatalyst to electrolyte‐penetrable, porous and defective nanosheets comprising NiOOH nanodomains, serving as highly active OER structures. This contrasts with previous reports on intermetallic bulk materials, which typically form core‐shell structures as a result of OER‐induced partial transformation. Regarding questions (iii) and (iv), electrochemical measurements confirmed the exceptional alkaline OER performance of the Ba_8_Ni_6_Ge_40_ precatalyst, surpassing state‐of‐the‐art commercial benchmarks. When employed as both cathode and anode in an assembled two‐electrode alkaline water electrolyzer, negligible activity decay was present over 10 days at around 550 mA cm^−2^. This remarkable catalytic behavior is attributed to the clathrate structure's characteristics and the high leaching tendency of both Ba and Ge during alkaline OER, facilitating significant active site availability. The electrochemically activated Ba_8_Ni_6_Ge_40_ exhibited approximately 25 times higher redox activity per loaded Ni site when compared with the metallic Ni reference. Additionally, a highly reversible phase transition between Ni(OH)_2_ and NiOOH has been confirmed both before and after OER for the reconstructed structure. We anticipate that the findings presented in this study not only trigger broader interest in novel clathrate intermetallics‐based electro(pre)catalysts but also lay the basis for designing highly efficient OER electrocatalysts.

## Conflict of Interests

The authors declare no conflict of interest.

## Supporting information



Supporting Information

Supporting Information

## Data Availability

The data that support the findings of this study are available from the corresponding author upon reasonable request.
