# Supplementary material for: Ba–Ni–Ge Clathrate Transformation Maximizes Active Site Utilization of Nickel for Enhanced Oxygen Evolution Performance
Source: Angew Chem Int Ed Engl. 2025 May 5;64(23):e202424743. doi: 10.1002/anie.202424743 (PMC12124348; doi:10.1002/anie.202424743)
Supplement: Supplementary file 2 — Supporting Information [file ANIE-64-e202424743-s001.docx]

Supporting Information

Ba-Ni-Ge Clathrate Transformation Maximizes Active Site Utilization of Nickel for Enhanced Oxygen Evolution Performance

Ziliang Chen,^#[a, e]^ Hongyuan Yang,^#[b]^ J. Niklas Hausmann,^[a]^ Stefan Mebs,^[d]^ Viktor Hlukhyy,^[c]^ Holger Dau,^[d]^ Matthias Driess,*^[b]^ and Prashanth W. Menezes*^[a,b]^

[a] Dr. Z. Chen, Dr. J. N. Hausmann, Dr. P. W. Menezes
Department of Materials Chemistry for Catalysis, Helmholtz-Zentrum Berlin für Materialien und Energie, Albert-Einstein-Str. 15, 12489 Berlin, Germany
E-mail: prashanth.menezes@helmholtz-berlin.de

[b] H. Yang, Prof. Dr. M. Driess, Dr. P. W. Menezes
Department of Chemistry: Metalorganics and Inorganic Materials, Technical University of Berlin, Straße des 17 Juni 135. Sekr. C2, 10623 Berlin, Germany
E-mail: prashanth.menezes@mailbox.tu-berlin.de, matthias.driess@tu-berlin.de

[c] Dr. V**.** Hlukhyy
Department Chemie, Technische Universität München, Lichtenbergstraße 4, 85747 Garching, Germany

[d] Dr. S. Mebs, Prof. Dr. H. Dau
Department of Physics, Free University of Berlin, Arnimallee 14, 14195 Berlin, Germany

[e] Dr. Z. Chen
Institute of Functional Nano and Soft Materials (FUNSOM), Jiangsu Key Laboratory for Carbon-based Functional Materials and Devices, Soochow University, Suzhou 215123, P.R. China

[#] These authors contributed equally to this work

Supporting information for this article is given via a link at the end of the document.

**Chemicals and Materials**

All chemical reagents (analytical grade) were directly employed as received without further purification. A commercially available potassium hydroxide solution (1.0 M KOH) was purchased from Sigma-Aldrich. The content of Fe was <0.05 ppm, determined by the inductively coupled plasma atomic emission spectroscopy (ICP-AES) measurement in our previous work which used the same KOH solution.^[1]^ Iridium oxide (IrO_2_) and ruthenium oxide (RuO_2_) were obtained from Alfa Aesar. Pure Ni powders with an average particle size of 1.2 µm were obtained from Hangzhou Xinchuan New Material Co., Ltd. The nickel foam (NF) and fluorine-doped tin oxide (FTO, resistivity 8~12 Ω/sq) were obtained from Recemat BV and Sigma Aldrich, respectively, which then served as the electrode substrate. Deionized water was utilized for the whole experiment.

**Synthesis of the Catalysts**

**Synthesis of Ba_8_Ni_6_Ge_40_**

Pieces of barium (ChemPur, 99.5%), nickel wire (Alfa Aesar, 99.98%) and germanium (EVOCHEM, 99.999%) were weighed with the nominal composition of 8Ba : 6Ni : 40Ge and arc melted using a modified Mini Arc Melting system (MAM-1, Johanna Otto GmbH) with a water-cooled copper-hearth in argon filled Glovebox (MBraun 20G, argon purity 99.996%). The resulting regulus was turned upside down three times and remelted in order to ensure homogenization. The sample is stable against air and moisture. The as-prepared Ba_8_Ni_6_Ge_40_ ingot was further manually crushed into powders and sieved with a 325-mesh griddle for structural characterization and performance testing.

**Synthesis of References**

The reference catalysts are all commercially purchased. The metallic nickel particles were bought from Hangzhou Sinchin New Material Co., Ltd. (China). The commercially available iridium oxide (IrO_2_, 99%) and ruthenium oxide (RuO_2_, 99%) were from Alfa Aesar.

**X-ray Diffraction**

In order to achieve the phase component and structure, powder X-ray diffraction (PXRD) was performed on a Bruker AXS D8 advanced automatic diffractometer equipped with a position-sensitive detector and curved germanium (111) primary monochromator. The PXRD pattern was further analyzed by the RIETAN-FP program which was developed based on the Rietveld refinement method.^[S2]^ The crystalline structural models of as-prepared compounds were three-dimensionally visualized by the VESTA program version 3.0.^[S3]^ The PXRD for the FTO and NF-deposited electrodes were conducted on Bragg-Brentano geometry under air, using a Rigaku SmartLab 3 kW diffractometer (Rigaku Corporation, Japan, Cu-*K*_α_ radiation: *λ* = 1.5418 Å). SmartLab Guidance software package (Rigaku Corporation, Japan; Version 2.1.0.0) was further used to acquire the PXRD data. The grazing incidence X-ray diffraction (GIXRD) with a beam knife was conducted using based on the PANalytical X-ray diffractometer with Cu Kα radiation (incident angle = 1.2°, step size = 0.05°, and scan rate = 0.2° s^–1^).

**Elemental Analysis**

The chemical composition of the Ba-Ni-Ge catalyst before and after the OER durability test was accurately determined by the ICP-AES analysis. Specifically, the catalysts were digested in aqua regia (the volume ratio of HNO_3_ to HCl is 1 : 3), then diluted with deionized water up to 15 mL. The calibration solutions of each element to be probed were prepared with different concentrations (0.1, 1, 10, and 50 ppm) from the standard solutions (1000 mg L^–1^ Single Element ICP-Standard Solution ROTI®STAR).

**Electron Microscopy**

We utilized scanning electron microscopy (SEM) on a ZEISS GeminiSEM500 NanoVP microscope to characterize the morphology of the catalysts before and after OER. The more in-depth insights into the microstructure of the catalysts were gained by transmission electron microscopy (TEM) using an FEI Tecnai G2 20 S-TWIN transmission electron microscope (FEI Company, Eindhoven, Netherlands) equipped with a LaB_6_-source at 200 kV acceleration voltage. The EDX analysis was carried out with an EDAX r-TEM SUTW Detector (Si (Li)-detector). Images were captured with a GATAN MS794 P CCD-camera. The SEM characterizations were performed at the Zentrum für Elektronenmikroskopie (ZELMI) of TU Berlin.

**X-ray Photoelectron Spectroscopy (XPS)**

In order to acquire information on the chemical state, the Kratos Axis Ultra X-ray photoelectron spectrometer (XPS) equipped with an Al *K*α (1486.7 eV) monochromatic radiation source (Kratos Analytical Ltd., Manchester, UK) was employed to the catalysts before and after the electrochemical reaction. The vacuum pressure in the analyzing chamber was kept at 2 x 10^−9^ Torr during the measurement. The high-resolution XPS spectra were collected for C 1s, Ni 2p, Ba 3p, and Ge 2p levels with a pass energy of 20 eV and step size of 0.1 eV. The binding energies were then calibrated against the C 1s peak energy position (285.0 eV). All the as-obtained data were analyzed by XPSPEAK software.

**Raman Spectroscopy**

The *in-situ* Raman spectra were collected by an Argon ion laser with a 532 nm emission for excitation (50 mW). Meanwhile, a confocal Raman spectrometer (Renishaw inVia) coupled with an electrochemical Raman cell was adopted, that comprises a three-electrode PVDF cell with a sapphire window (0.5 mm thickness). The sample surface was focused via a 50X long focus objective. During the process of measurements, the sapphire window was less than 0.1 mm from the working electrode surface, through which, a very thin solution layer was kept on the surface of the working electrode. Thus, the weakening of the Raman signals brought by the solution layer was suppressed as much as possible. To prepare the catalyst films that were used for measurements, the catalyst powders were electrophoretically deposited on FTO at an area of 1 × 1 cm. The *in-situ* Raman spectra were collected in 1.0 M KOH electrolyte under the applied potentials from 1.0 to 1.8 V *vs.* RHE (an increasing interval of 0.1 V).

**X-ray Absorption Spectroscopy (XAS)**

To shed more light on the local atomic and electronic structure, we also carried out the X-ray absorption near-edge structure (XANES) and extended X-ray absorption fine structure (EXAFS) studies under *ex-situ* and quasi *in-situ* conditions. The EXAFS and XANES spectra were recorded at the BESSY II synchrotron radiation source located at the Helmholtz-Zentrum Berlin. The measurements were run at the KMC-3 bending-magnet beamline at 20 K in a helium-flow cryostat (Oxford-Danfysik). The incident beam energy was chosen using a Si(111) double-crystal monochromator. The measurements at the Ni and Ge *K*-edges as well as the Ba *L3* edge were carried out in fluorescence mode (fluorescence energy region selected) using a 13-element energy-resolving Ge detector (Canberra) cooled with liquid nitrogen (N_2_). The extracted spectra were weighted by *k*^3^ and simulated in k-space (Ni: E_0_ = 6547 eV, Ge: E_0_ = 11113 eV, Ba: E_0_ = 5247 eV). The in-house software (SimXLite, programmed by Dr. Petko Chernev) was used to conduct the EXAFS simulations after calculating phase functions *via* the FEFF program (version 8.4, self-consistent field option activated). The EXAFS spectra were simulated in the data range of Ni: *k* = (2~12) Å^–1^ and Ge: *k* = (2~10) Å^–1^. Furthermore, we optimized the EXAFS simulation by minimizing the error sum from the summation of the squared deviations between measured and simulated values (least-squares fit). The fitting was operated *via* the Levenberg-Marquardt method with numerical derivatives.^[S4]^ The fit parameters error ranges were assessed from the covariance matrix of the fit. Cosine windows covering 10% of the low k-side and 10% of the high k-side of the spectra were utilized to calculate the Fourier transformation. To prepare the sample for the quasi *in-situ* XAS tests, the Ba_8_Ni_6_Ge_40_ was deposited on the FTO substrate (Ba_8_Ni_6_Ge_40_/FTO) using electrophoretic loading. The as-deposited film was then freeze-quenched after 24 h of OER chronoamperometry (CA) at 1.55 V *vs*. RHE (denoted as “1.55 V”) *via* liquid N_2_ under vigorous Ar gas flow and stored in liquid N_2_. The sample 1.55 V was further subject to CA at 1.0 V *vs.* RHE for 24 h (denoted as “1.0 V”) and rapidly freeze-quenched following the same procedures. In addition, the freshly prepared Ba_8_Ni_6_Ge_4_/FTO were also freeze-quenched after 10 cycles of cyclic voltammetry (CV) from 1.1 to 1.75 V *vs.* RHE (denoted as Activation).

**Electrophoretic Deposition (EPD) of Catalysts**

The EPD was realized by the occurrence of the migration and deposition of charged particles in suspension initiated by applying an electric field between the anode and the cathode. In our deposition protocol, 20 mg of the electrocatalyst powder was first dispersed in 10 ml acetone, followed by the addition of 3 mg of iodine. This solution was further ultrasonicated for 20 min. The detailed mechanism involving EPD is schematically shown in the following equation, in which iodine acetone reacted with iodine through the keto-enol tautomerism to produce protons. Then, the released protons are adsorbed on the surface of the suspended particles by making them positively charged. The applied electric field enables the positively charged particles to migrate towards and deposit on the cathode. The details about this EPD method can also be found elsewhere.^[S5]^ Based on this, the as-deposited catalyst films on the FTO and NF were 1 mg cm^–2^ and 2 mg cm^−2^, respectively.

**
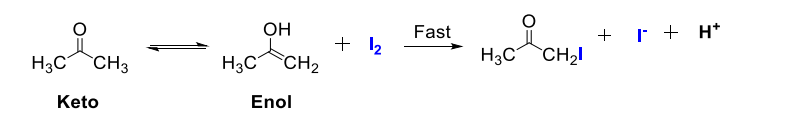
**

**Electrochemical Measurements**

The typical electrochemical measurements were carried out in a standard three-electrode electrochemical cell in 1 M aqueous KOH electrolyte using a potentiostat (SP-200, BioLogic Science Instruments) controlled by the EC-Lab v10.20 software package. The substrates (NF and FTO) supporting deposited catalyst films served as the working electrodes, while a Pt wire (0.5 mm diameter × 230 mm length; A-002234, BioLogic) and a Hg/HgO (CH Instruments, Inc.) worked as the counter and reference electrode, respectively. CV and linear sweep voltammetry (LSV) were carried out at a low scan rate of 5 mV s^−1^ and corrected with an applied iR compensation of 95%. The potentials measured in this work were recalculated to the reversible hydrogen electrode (RHE) through the following equation in 1.0 M aqueous KOH, *E*_(RHE)_ = *E*_(Hg/HgO)_ + 0.098 V + (0.059 × pH) V. The chronoamperometric measurements (CA) were performed in 1 M aqueous KOH at the desired constant potentials against RHE. We applied the steady-state method to determine the Tafel slopes,^[S6]^ which were realized by running CA for 3 min at each fixed potential. The applied potentials were increased in steps of 15 mV. The current density associated with each potential was subsequently utilized to calculate the Tafel slope, according to the equation: *η* = *b*log *j* + *a*. Herein, *η*, *j*, and *b* represent the overpotentials (mV) obtained from the associated applied potentials, current densities (mA cm^−2^), and Tafel slope (mV dec^−1^), respectively. The electrochemical impedance spectroscopy (EIS) was recorded at 1.55 V *vs*. RHE for the samples deposited on FTO and NF, respectively. The amplitude of the sinusoidal wave was defined in a frequency range from 100 kHz to 1 mHz. The charge transfer resistance (*R*_ct_) was determined by the diameter value of the semicircle in the Nyquist plots. Note that all the crucial electrochemical tests, including CV, LSV, Tafel, and EIS measurements, were performed after the CV activation until a stable curve was attained (as exemplified in Figure S11). The long-term stability was directly tested by CA without CV activation and operated at a desired potential which was constant during the measurement. The two-electrode electrolyzer used in the current work was assembled by Ba_8_Ni_6_Ge_40_/NF as both cathode and anode, where no reference electrode and diaphragm/membrane were adopted. The surface working area of these two electrodes was both 0.4 * 0.5 cm^2^. The 1 M KOH aqueous electrolyte was employed. The working temperature for all electrochemistry was room temperature otherwise specially mentioned.

**Turnover Frequency (TOF) Estimation**

The TOF refers to the moles of O_2_ per moles of total metal content (Ni in our case) evolved per second (s^−1^). The redox-normalized TOF values of the measured catalysts were obtained based on the equation:^[S7]^ TOF (s^−1^) = (*j* × *N*_A_) / (*z* × *F* × *n*), in which j is the OER current density, *N*_A_, *F*, and *z*, mean the Avogadro number (6.02×10^23^), the Faraday constant (96485 C mol^−1^), electron transfer number for the overall reaction (*z* = 4 for OER). *n* represents the number of the participated redox-active Ni sites (per cm^2^ of the geometrical sample area).

**Table S1.** Lattice parameters, atomic coordinates, isotropic thermal parameters (*B* values), and occupation numbers (g values) for the Ba_8_Ni_6_Ge_40_ compound determined from the Rietveld refinement of PXRD shown in Figure 1a of the main text.

| atom | site | *g* | *x* | *y* | *z* | *B* (Å^2^) |
| --- | --- | --- | --- | --- | --- | --- |
| Ba | 2*a* | 1 | 0 | 0 | 0 | 0.011(6) |
| Ni | 6*c* | 1 | 0.25 | 0 | 0.5 | 0.183(3) |
| Ba | 6*d* | 1 | 0.25 | 0.5 | 0 | 0.266(1) |
| Ge | 16*i* | 1 | 0.1837(2) | *x* | *x* | 0.302(8) |
| Ge | 24*k* | 1 | 0 | 0.3175(3) | 0.1221(3) | 0.106(3) |
| **Space group**: *Pm*-3*n* (223); *a* = *b* = *c* = 10.6754(8) Å, *V* = 1216.6 (2) Å^3^ | | | | | | |

**
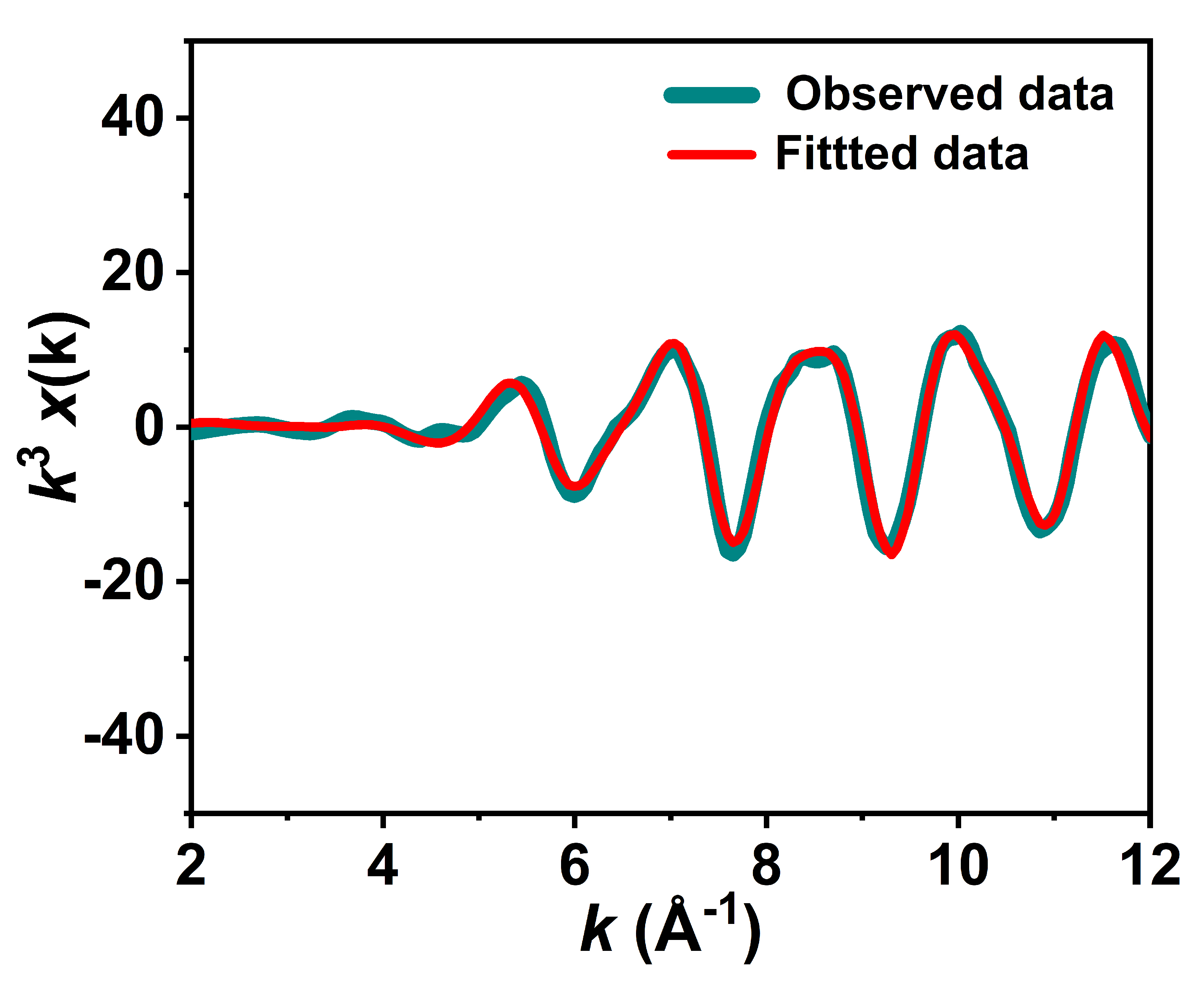
**

**Figure S1.** The Ni K-edge *k*^3^-weighted EXAFS spectra for the as-prepared Ba_8_Ni_6_Ge_40_ powder. The fit parameters are shown in Table S2.

**Table S2.** Structural parameters for Ba_8_Ni_6_Ge_40_ powder obtained from Ni K-edge EXAFS fitting of the spectra shown in Figure 1c of the main text. The bond distances obtained from PXRD data (PDF#97-005-7034) are also recorded for comparison, which were marked by *.

| EXAFS | Path | *R* (Å) | *σ* (Å) | *N* | *R*_f_ |
| --- | --- | --- | --- | --- | --- |
|  | Ni-Ge | 2.29±0.01 | 0.064±0.001 | 4 | 14.3 |
|  |  | 2.3790* |  |  |  |
|  | Ni-Ba | 3.88±0.02 | 0.064±0.001 | 2 |  |
|  |  | 3.7743* |  |  |  |
|  | Ni-Ge | 3.96±0.01 | 0.064±0.001 | 6 |  |
|  |  | 3.9684* |  |  |  |

*Note:* The range of the fits was 2~12 Å^–1^. *R* is the absorber-backscatter distance, *N* means the EXAFS coordination number, *σ* represents the Debye Waller factor. The *R*_f_ represents the fit error sum in %. The amplitude reduction factor, *S*_0_^2^ (k), was 0.9 in all refinements. The errors represent the 68% confidence interval of the respective fit parameter. N was constrained to represent the XRD structure, *σ* was restrained to be the same for all shells.


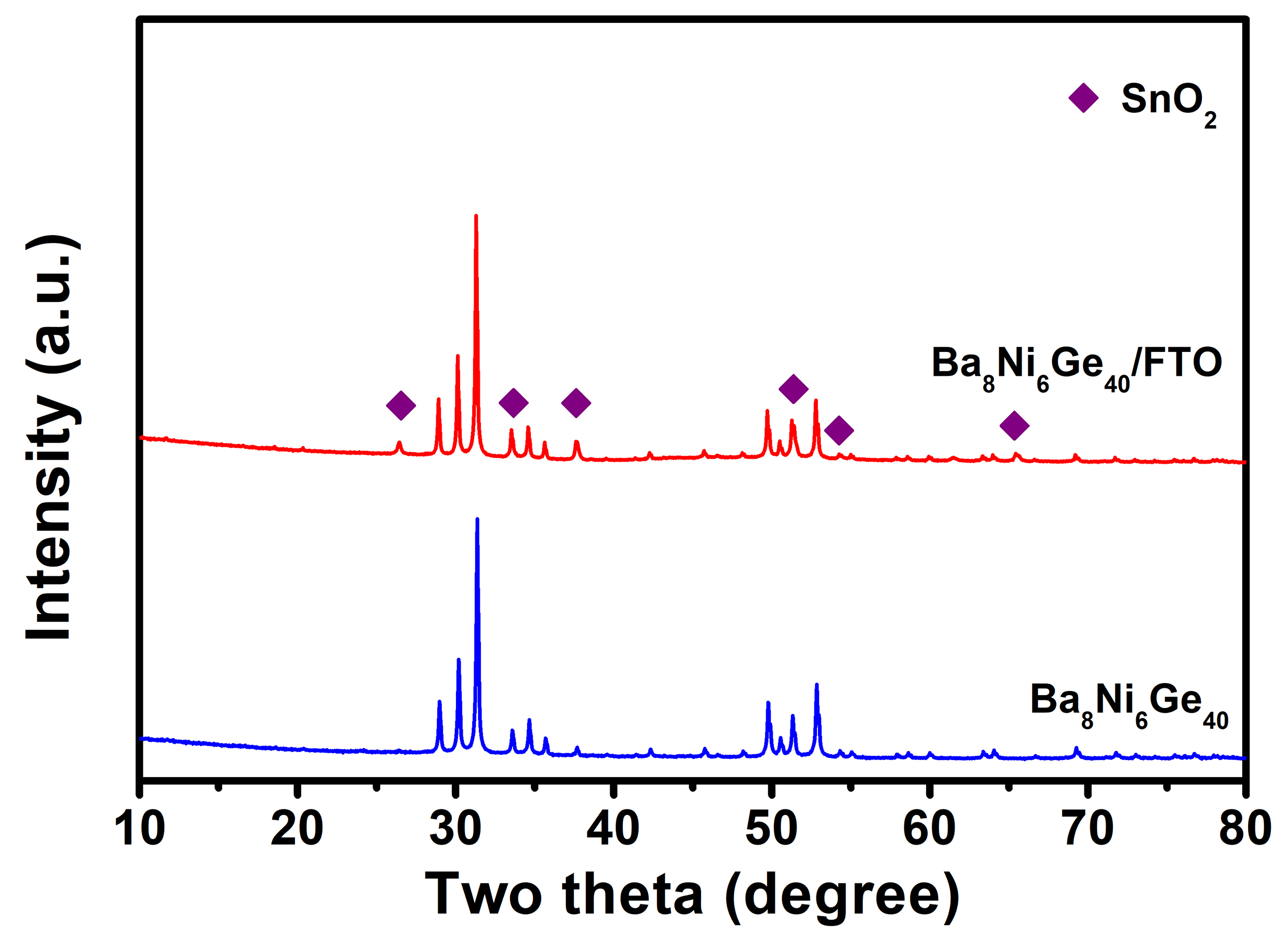


**Figure S2.** The PXRD pattern of the as-deposited Ba_8_Ni_6_Ge_40_/FTO electrode. The violet diamonds represent the FTO peaks. The XRD peaks are consistent with PDF#97-005-7034.


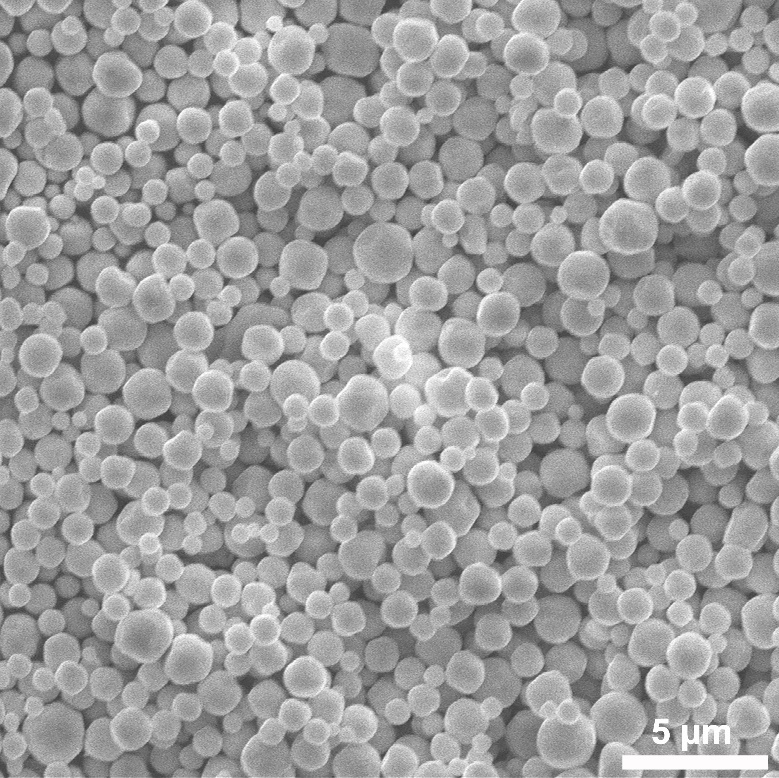


**Figure S3.** SEM image of the metallic Ni reference used for the targeted sample Ba_8_Ni_6_Ge_40_.


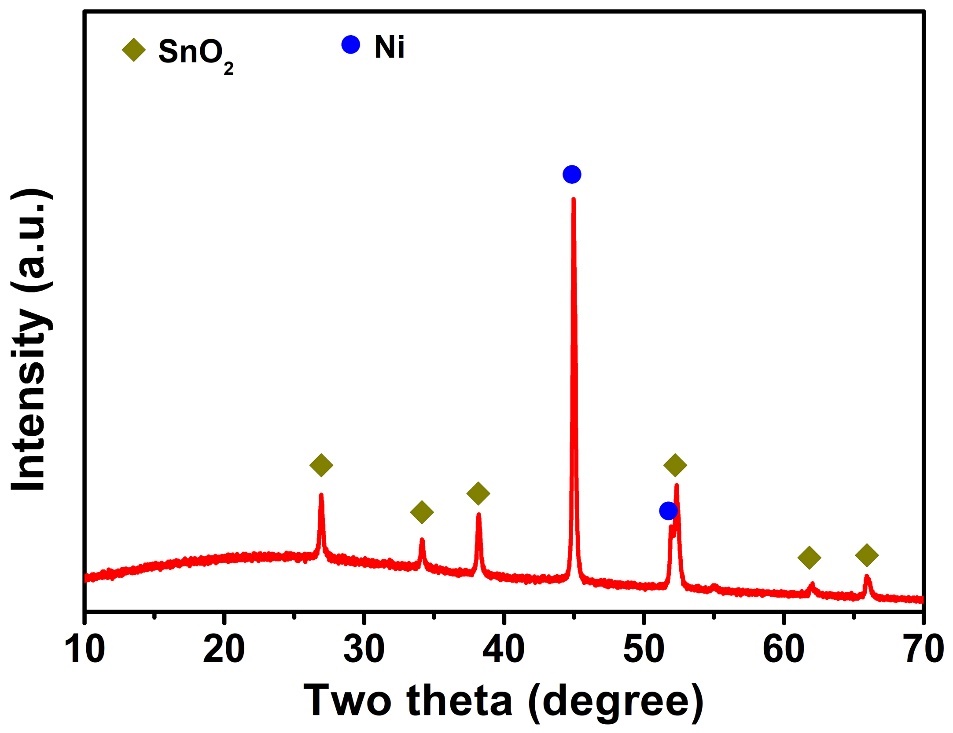


**Figure S4.** PXRD pattern of the metallic Ni reference deposited on FTO (Ni/FTO) used for the targeted sample Ba_8_Ni_6_Ge_40_/FTO.


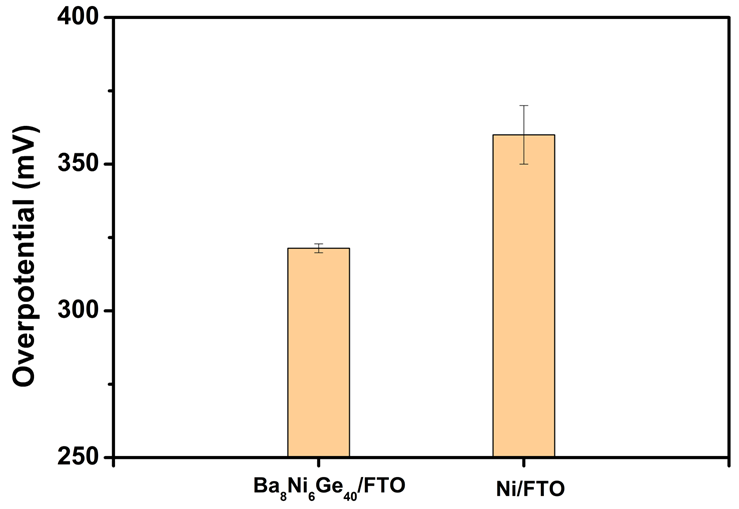


**Figure S5.** The mean values of the overpotentials with standard deviation at 10 mA cm^–2^ current density for Ni/FTO and Ba_8_Ni_6_Ge_40_/FTO based on three independent CV measurements (the values of the forward-scanned curves were used).

**Table S3.** The comparison of OER activity of Ba_8_Ni_6_Ge_40_/FTO with those of recently reported FTO-supported intermetallics and Ni-based OER catalysts in 1 M KOH electrolyte. The abbreviation rGO denotes reduced graphene oxide.

| **Electrode** | **Loading  (mg cm^−2^)** | **Temperature** | **Overpotential @ 10 mA cm^–2^** | **Ref.** |  |
| --- | --- | --- | --- | --- | --- |
| FeSi/FTO | ~0.4±0.1 | R.T.^a^ | 415±4 mV | [S1] |  |
| NiGe | ~0.4±0.08 | R.T. | 322±2 mV | [S9] |  |
| Ni(OH)_2_/*β*-like FeOOH | NA | R.T. | 300 mV | [S10] |  |
| Ni-Fe-O | NA | NA | 330 mV | [S11] |  |
| Ni-Co-S-Se | 0.1 | NA | 340 mV | [S12] |  |
| CuNi | ~5 | R.T. | 294 mV | [S13] |  |
| FeAs | 0.4 ±0.1 | R.T. | 395±6 mV | [S14] |  |
| Ni_2_P | ~1 | R.T. | 330 mV | [S15] |  |
| Ni_12_P_5_ | ~1 | R.T. | 295 mV | [S15] |  |
| NiFeO*_x_*H*_y_* | 6.1056 * 10^–3^ | R.T. | 298 mV | [S16] |  |
| Ni-Co-Se/FTO | 0.072 | NA | 340 mV | [S17] |  |
| Ni_2_Si/FTO | 0.4 ±0.1 | R.T. | 321 mV | [S18] |  |
| CoSn/FTO | ~1 | R.T. | 299 mV | [S19] |  |
| Mn_3_N_2_/FTO | ~1 | R.T. | 390 mV | [S20] |  |
| NiFeCo/FTO | NA | NA | 300 mV | [S21] |  |
| Ni-Co-O/FTO | | NA | NA | 299 mV | [S22] |
| Ni-FeOOH/rGO/FTO | NA | NA | 260 mV | [S23] |  |
| Ni_2_P-NW//FTO | 0.1 | Ambient temperature | 340 mV | [S24] |  |
| Ni(OH)_2_/FTO | 0.57±0.04 * 10^–3^ | NA | 324 ± 4 mV | [S25] |  |
| Ni_4_-MOF/FTO | 0.1 | 25 °C | 282 mV | [S26] |  |
| Ba_8_Ni_6_Ge_40_/FTO | 1 | R.T. | 321 ± 2 mV | This work |  |

^a^ R.T. means the room temperature, NA means not available

**
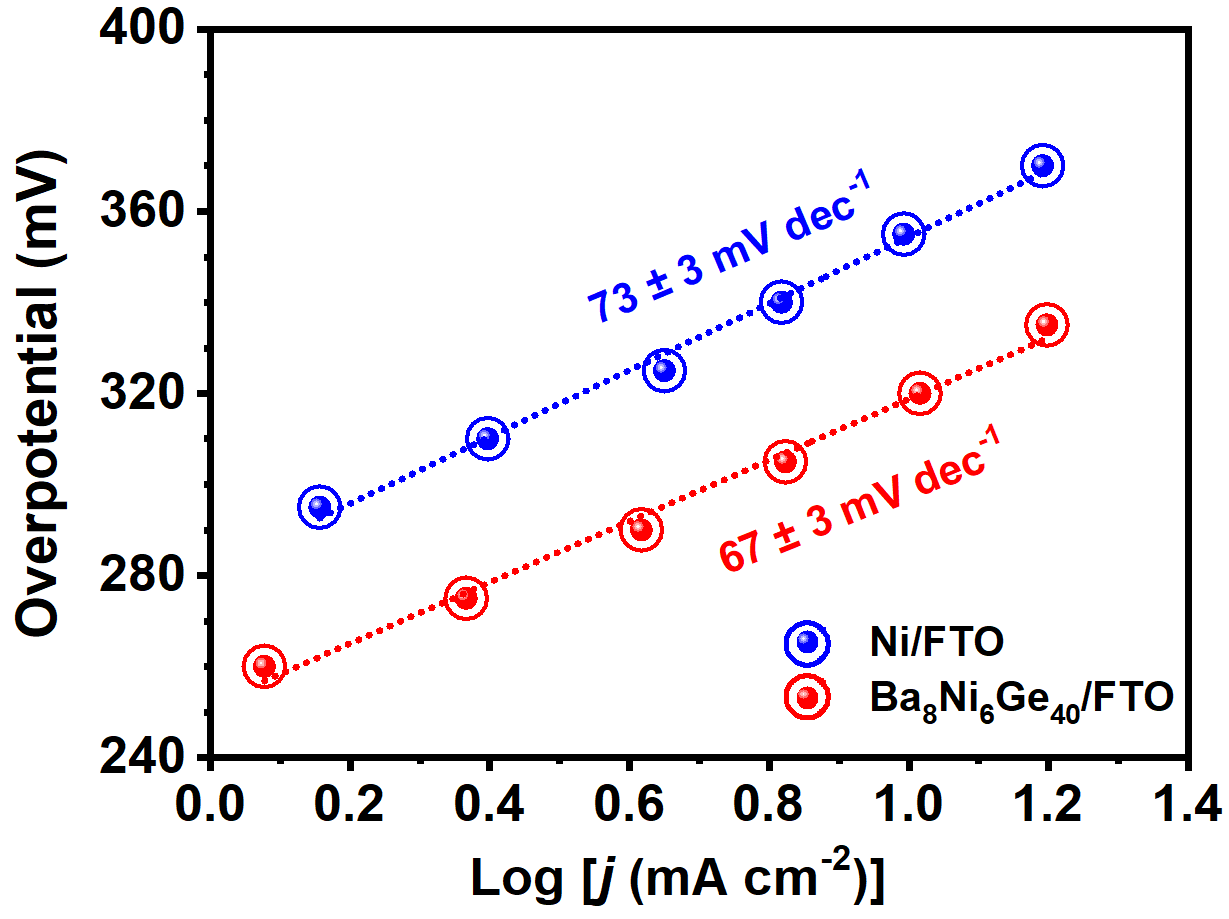
**

**Figure S6.** Comparison of Tafel slopes between Ni/FTO and Ba_8_Ni_6_Ge_40_/FTO electrode in 1.0 M KOH electrolyte.


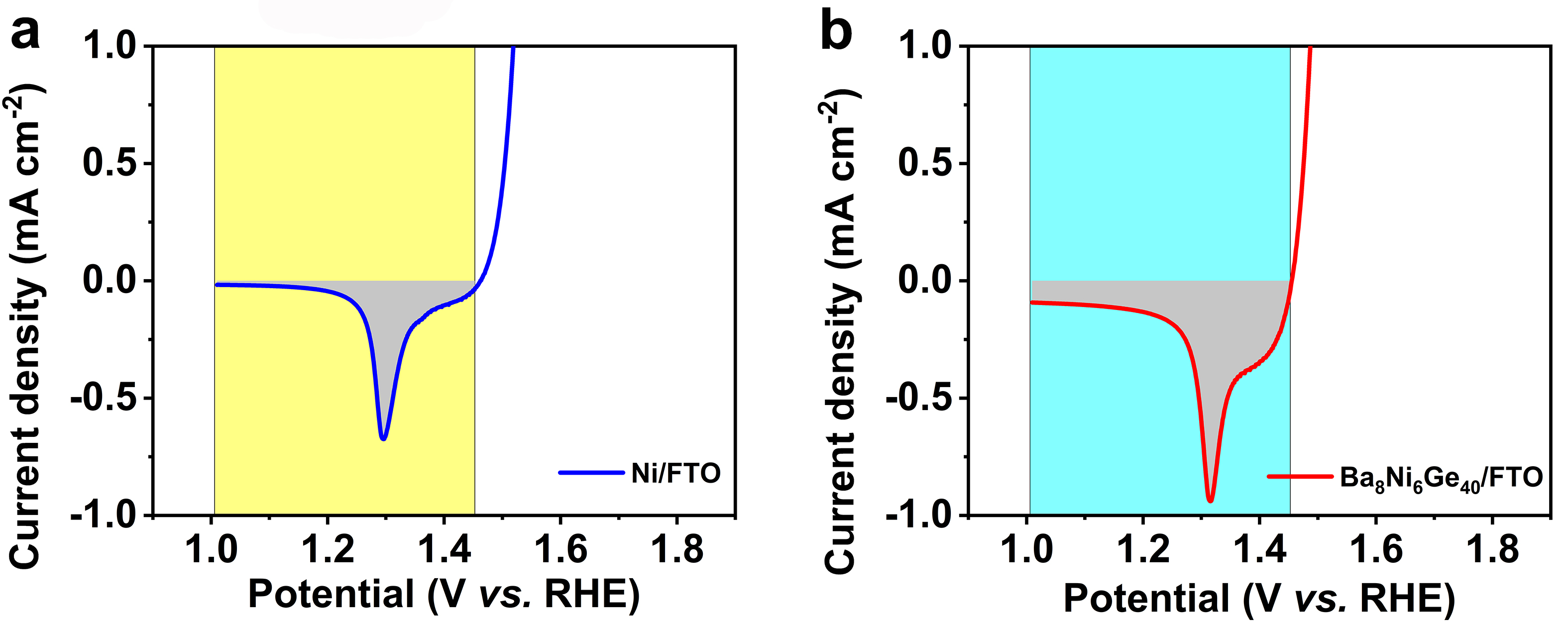


**Figure S7.** The integrated reduction peaks (from CV curves in Figure 3a of main text) of (a) Ni/FTO and (b) Ba_8_Ni_6_Ge_40_/FTO.

***For Ba_8_Ni_6_Ge_40_/FTO***:

Calculated area associated with the reduction peak = **0.11155 × 10****^−3^ V A cm^−2^**

Hence the associated charge is = 0.11155 × 10^−3^ V A cm^−2^ / 0.005 V s^−1^

= 22.31 × 10^−3^ A s cm^−2^

= 22.31 × 10^−3^ C cm^−2^

Now, the number of electron transferred is = 22.31 × 10^−3^ C cm^−2^ / 1.602 × 10^−19^ C

= 13.926 ×10^16^ cm^−2^

Since the reduction of Ni^III^ to Ni^II^ is a single electron transfer reaction, the number of electrons calculated above is the same as the number of the redox-active Ni sites (per cm^2^ of the geometrical sample area) involved in OER catalysis.

Therefore, *n* = **13.926 ×10^16^**

At an overpotential of 0.3 V,

TOF = [(6.1 × 10^−3^) × (6.023× 10^23^)] / [96485 × 4 × (13.926 × 10^16^)]

**TOF = 0.068 s^−1^**

***For Ni/FTO***:

Calculated area associated with the reduction peak = **0.0549 × 10^−3^ V A cm^−2^**

Hence the associated charge is = 0.0549 × 10^−3^ V A cm^−2^ / 0.005 V s^-1^

= 10.98 × 10^−3^ A s cm^−2^

= 10.98 × 10^−3^ C cm^−2^

Now, the number of electron transferred is = 10.98 × 10^−3^ C cm^−2^ / 1.602 × 10^−19^ C

= 6.854 ×10^16^ cm^−2^

Since the reduction of Ni^III^ to Ni^II^ is a single electron transfer reaction, the number of electrons calculated above is the same as the number of the redox-active Ni sites (per cm^2^ of the geometrical sample area) involved in OER catalysis.

Therefore, *n* = **6.854 ×10^16^**

At an overpotential of 0.3 V,

TOF = [(1.8 × 10^−3^) × (6.023× 10^23^)] / [96485 × 4 × (6.854 ×10^16^)]

**TOF =** **0.041 s^−1^**


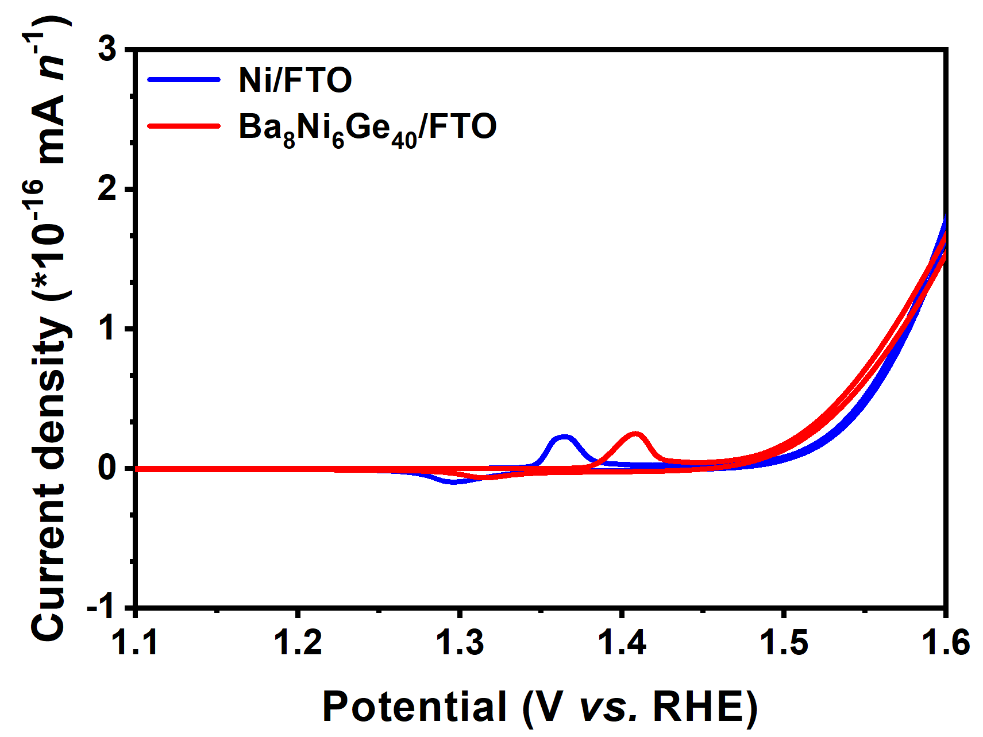


**Figure S8.** Current densities of the OER CV normalized against the associated numbers of the participated redox-active Ni sites per cm^2^ of the geometrical sample area (*n* values) for Ni/FTO and Ba_8_Ni_6_Ge_40_/FTO.


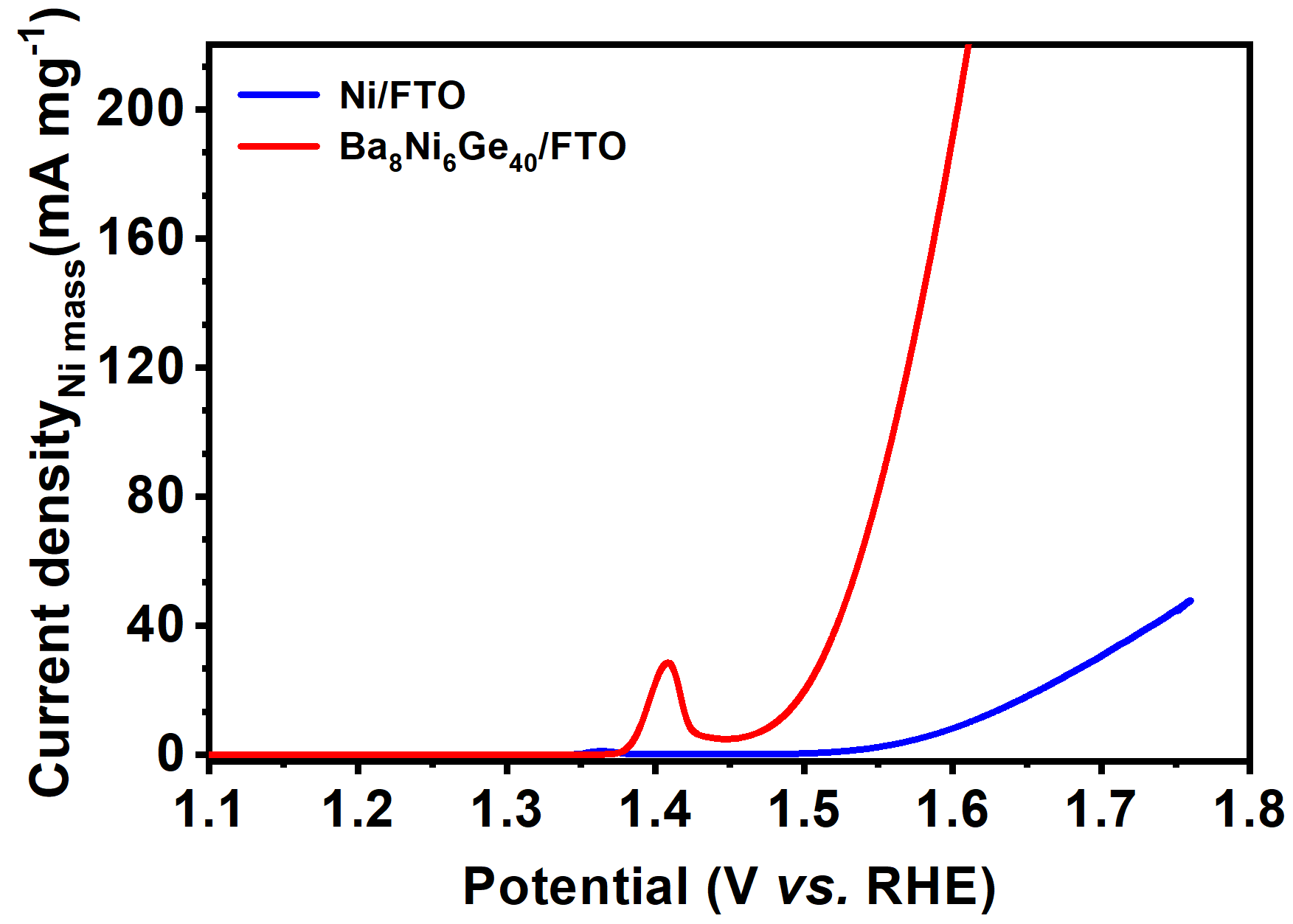


**Figure S9.** Comparison of LSV curves (5 mv s^−1^) based on the Ni mass content of Ni/FTO and Ba_8_Ni_6_Ge_40_/FTO electrode in 1.0 M KOH electrolyte.


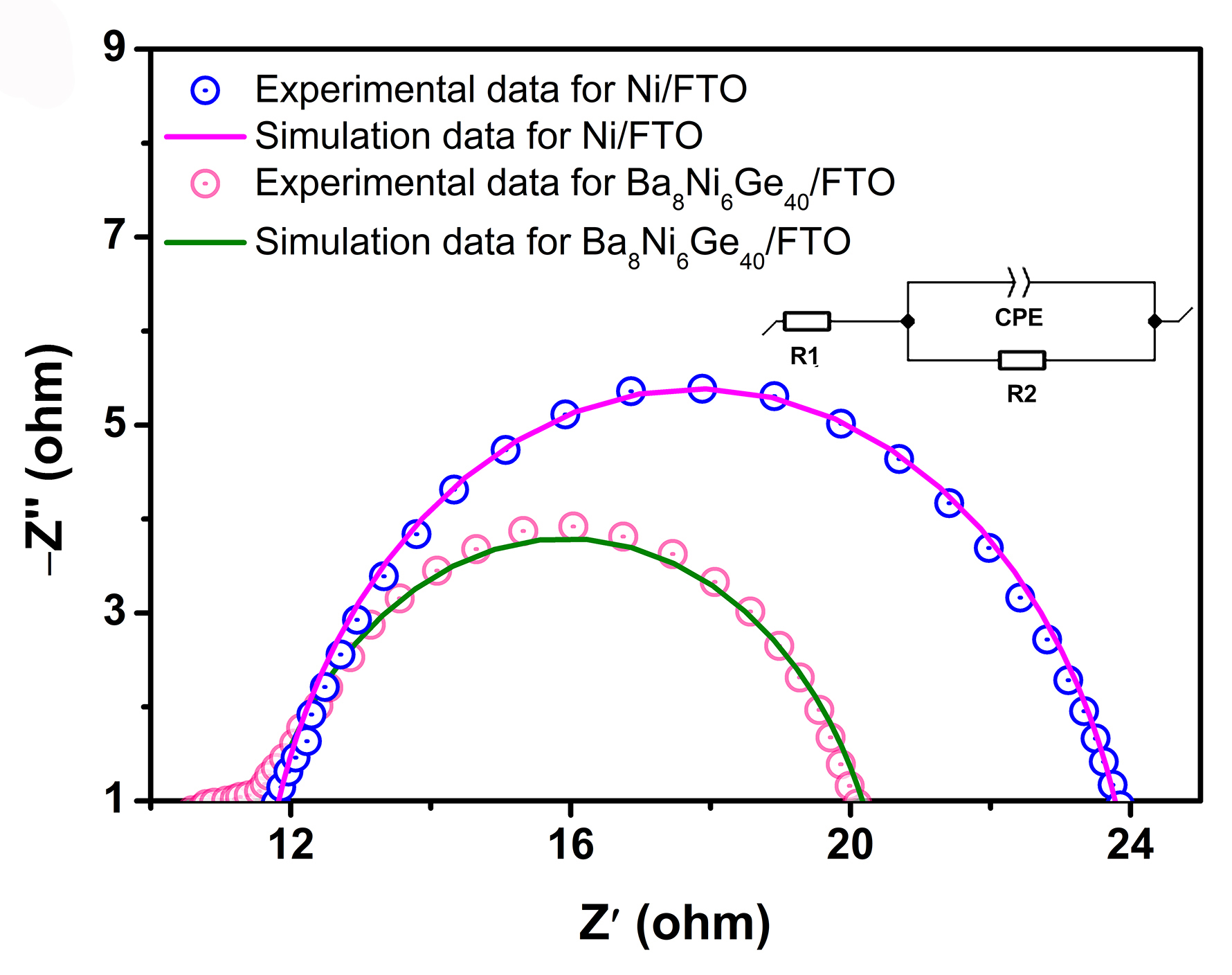


**Figure S10.** Comparison of EIS spectra between Ni/FTO and Ba_8_Ni_6_Ge_40_/FTO electrodes at the potential of 1.55 V *vs*. RHE in 1.0 M KOH electrolyte. Note that the pre-activation was performed before measurement.

**Table S4.** *R*_ct_ (Ω), *R*_s_ (Ω), CPE (*F* x *s*^(^*^a2^*^-1)^) and *a2* for Ni/FTO, Ba_8_Ni_6_Ge_40_/FTO, Ni/NF, and Ba_8_Ni_6_Ge_40_/NF obtained from EIS fitting at 1.55 V (*vs.* RHE).

| **Sample** | **R_s_ (Ω)** | **R_ct_ (Ω)** | **CPE** | ***a2*** |
| --- | --- | --- | --- | --- |
| Ni/FTO | 11.6 | 12.41 | 0.0116 | 0.9104 |
| Ba_8_Ni_6_Ge_40_/FTO | 11.4 | 9.071 | 0.01123 | 0.8867 |
| Ni/NF | 0.95 | 4.632 | 0.01953 | 0.7618 |
| Ba_8_Ni_6_Ge_40_/NF | 1.35 | 3.0 | 0.04349 | 0.8545 |


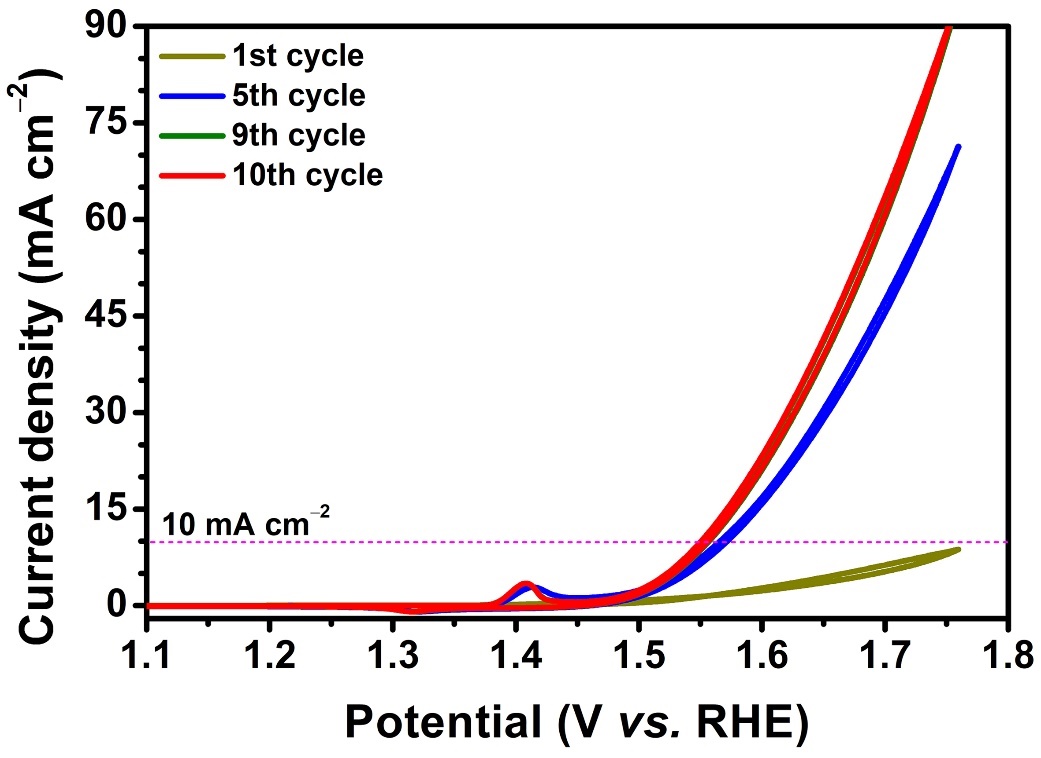


**Figure S11.** CV activation of the FTO-supported Ba_8_Ni_6_Ge_40_ precatalyst in 1.0 M KOH electrolyte (5 mV s^−1^). It can be observed that a stable CV can be obtained after 10 cycles of CV activation.


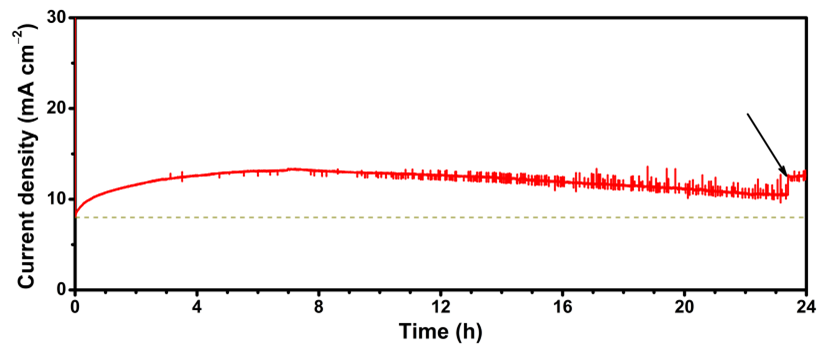


**Figure S12.** CA curve of the FTO-supported Ba_8_Ni_6_Ge_40_ precatalyst at 1.55 V vs. RHE (corresponding to the current density of ~10 mA cm^−2^) in 1.0 M KOH electrolyte. The position shown by the black arrow means the replenishment of electrolyte.


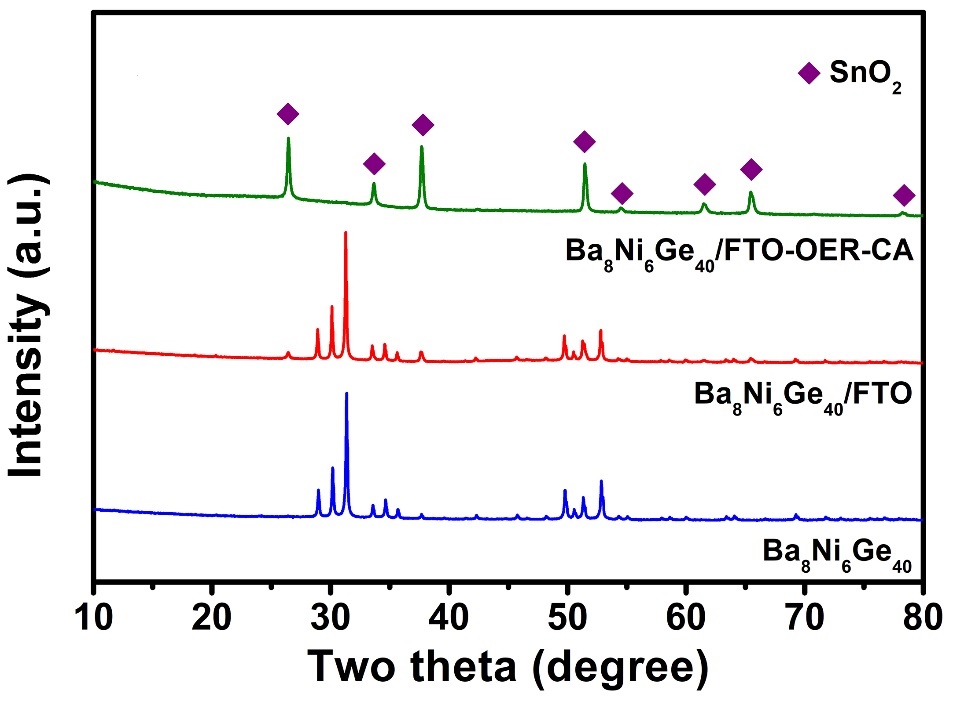


**Figure S13.** PXRD patterns of Ba_8_Ni_6_Ge_40_/FTO electrode before and after OER CA at 1.55 V *vs*. RHE in 1.0 M KOH electrolyte.


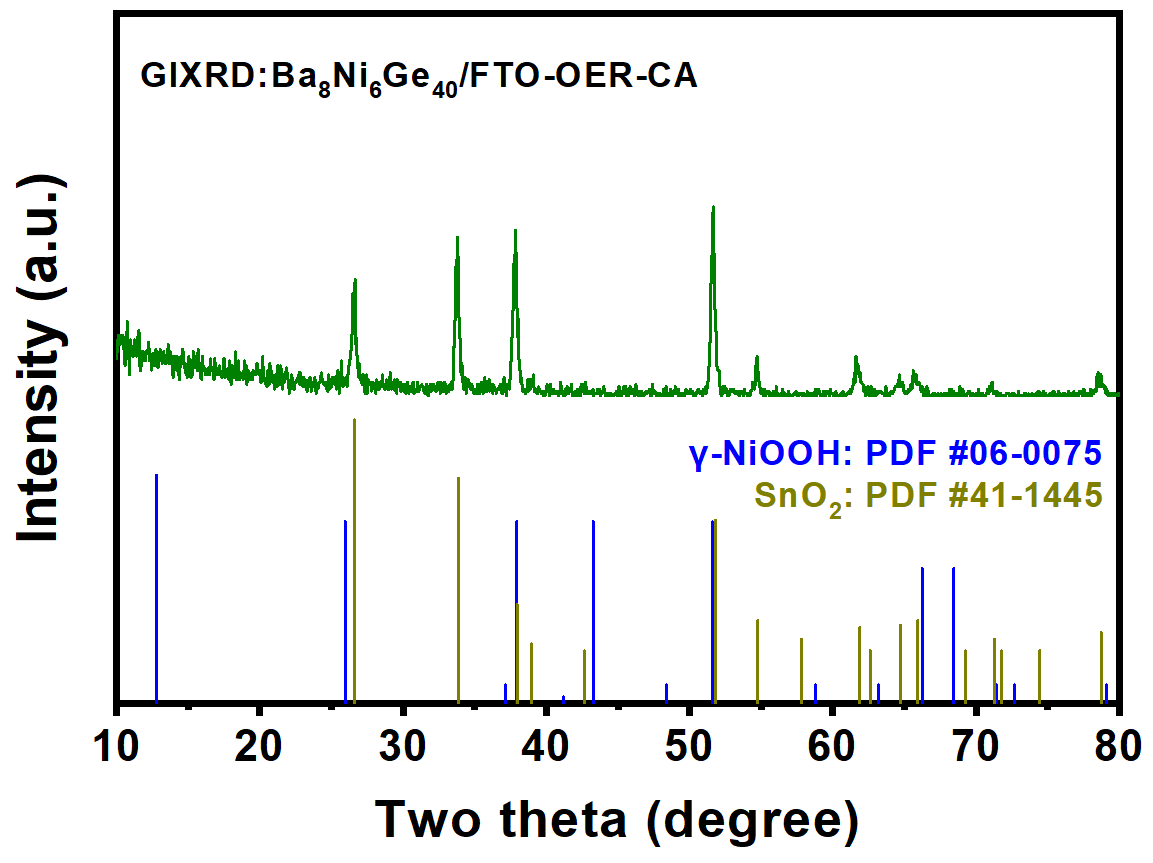


**Figure S14.** GIXRD pattern of the post-OER Ba_8_Ni_6_Ge_40_ film (supported on FTO and treated with a 24 h OER CA at 1.55 V *vs.* RHE).

**
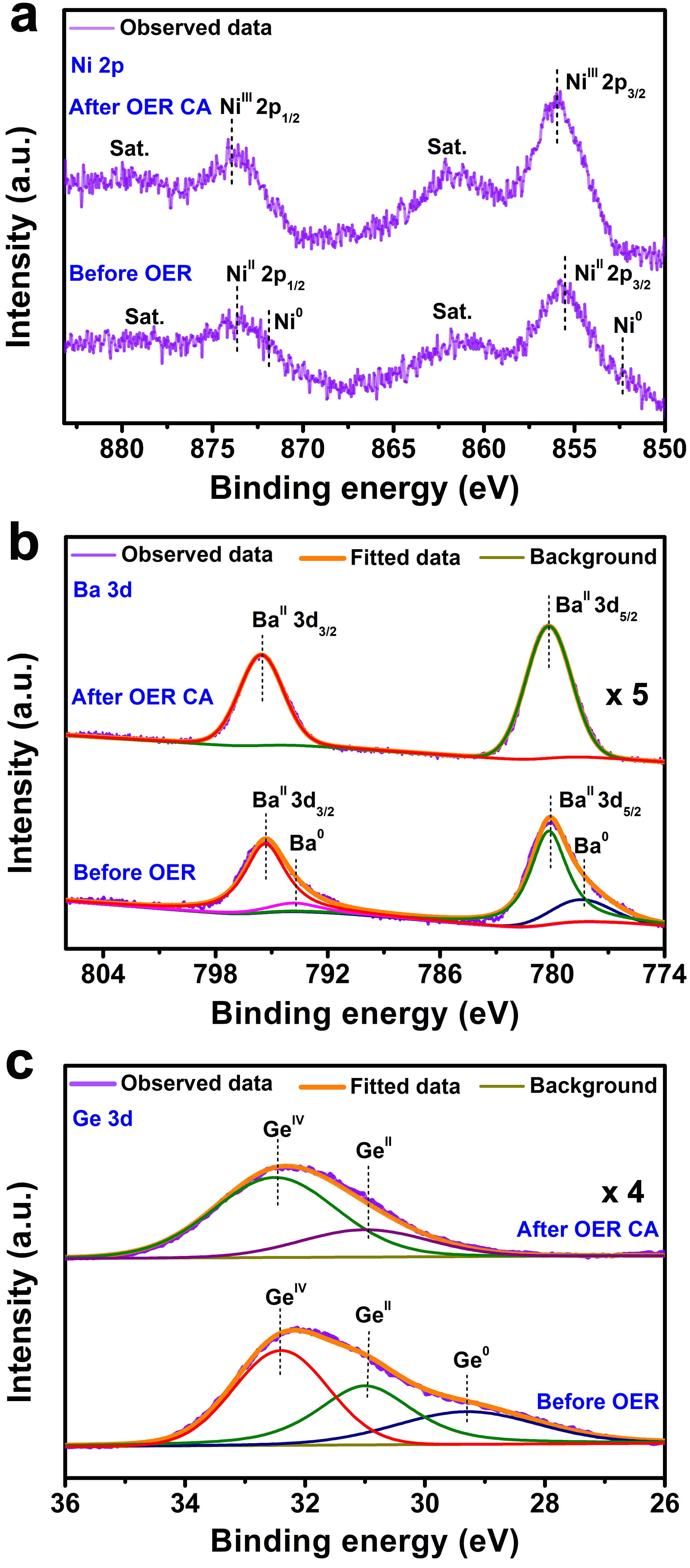
**

**Figure S15.** Comparison of high-resolution XPS spectra of (a) Ni 2p, (b) Ba 3d, and (c) Ge 3d in Ba_8_Ni_6_Ge_40_/FTO electrode before and after OER CA at 1.55 V *vs*. RHE in 1.0 M KOH electrolyte.


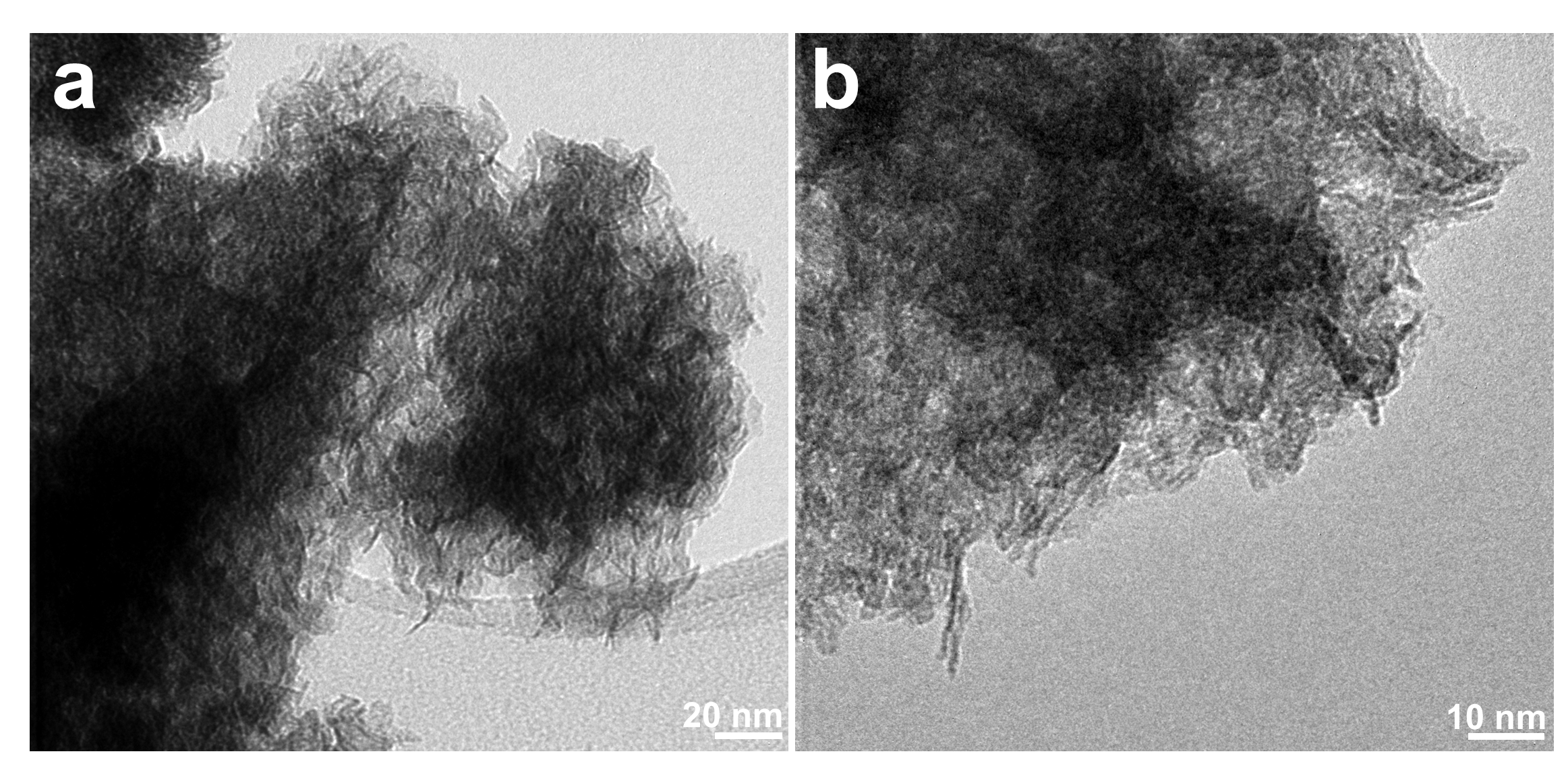


**Figure S16.** (a,b) TEM images of Ba_8_Ni_6_Ge_40_ after 24 h CA at 1.55 V *vs.* RHE.

**
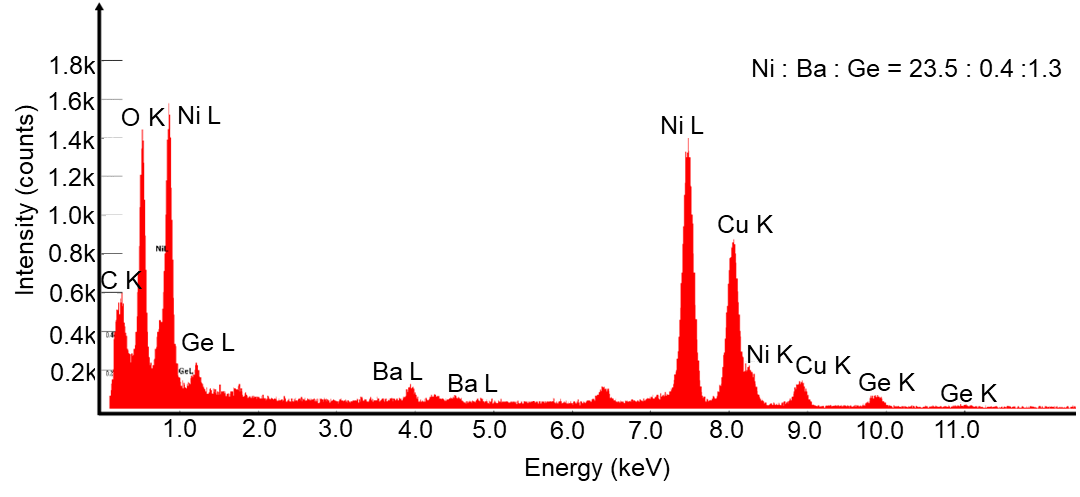
**

**Figure S17.** The EDX result recorded for the Ba_8_Ni_6_Ge_40_/FTO electrode after OER CA at 1.55 V *vs*. RHE in 1.0 M KOH electrolyte showing massive leaching of Ge and Ba.


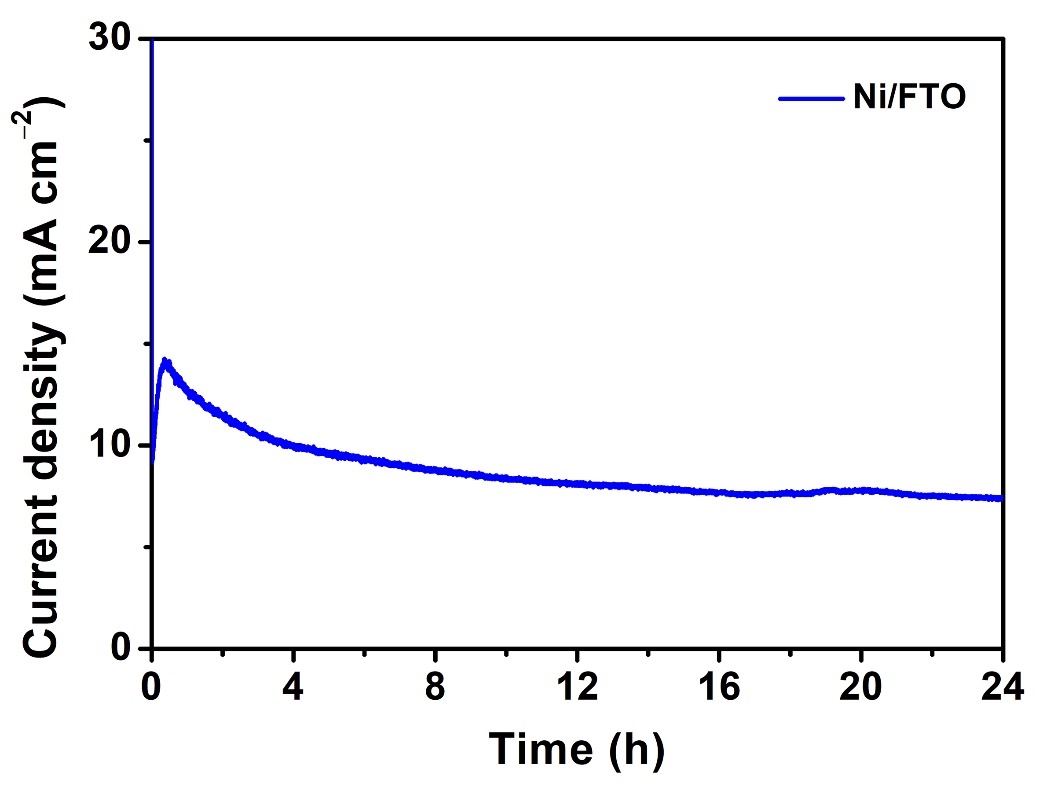


**Figure S18.** CA curve of Ni/FTO electrode at a potential of 1.59 V *vs.* RHE (corresponding to the current density of around 10 mA cm^−2^) in 1.0 M KOH.


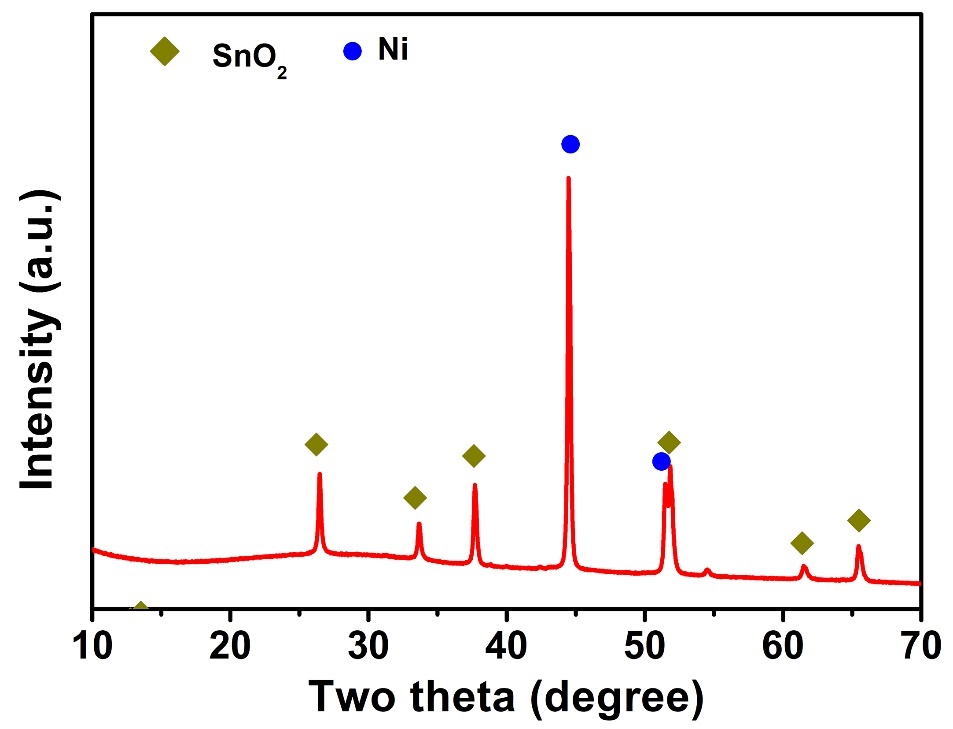


**Figure S19.** PXRD pattern of Ni/FTO electrode after OER CA in 1.0 M KOH electrolyte showing that the metallic Ni core was preserved during OER electrocatalysis.


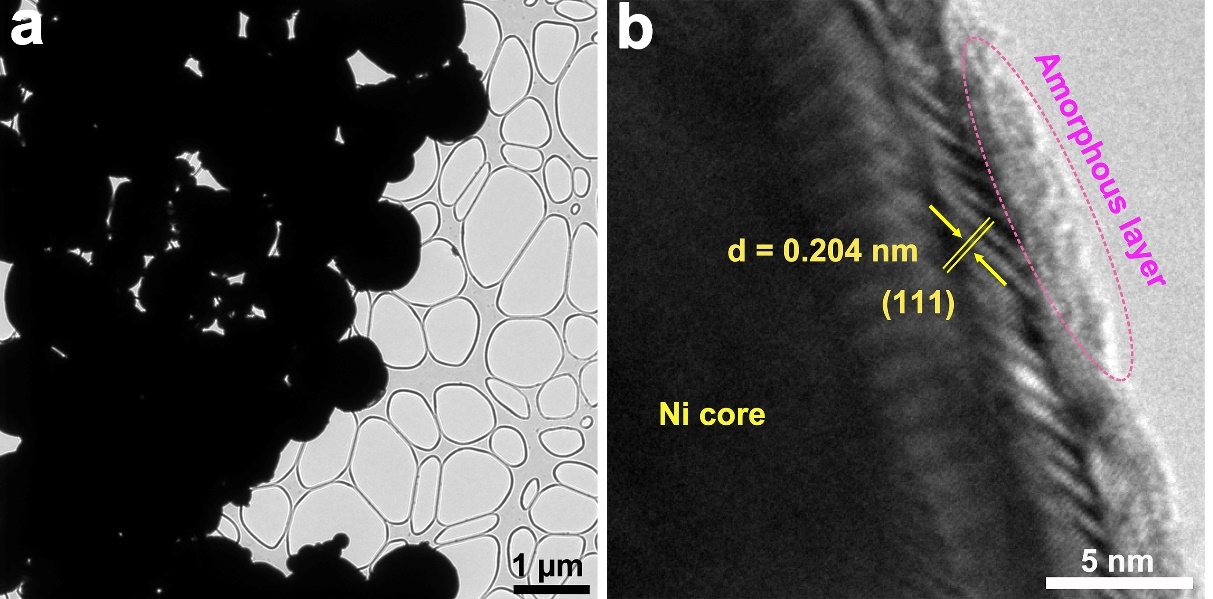


**Figure S20.** (a) TEM image and (b) high resolution TEM image of particle scratched off from Ni/FTO electrode after OER CA. The results still showed the average particle size of 1 µm and an amorphous layer with a thickness of 5 nm covering the Ni core.


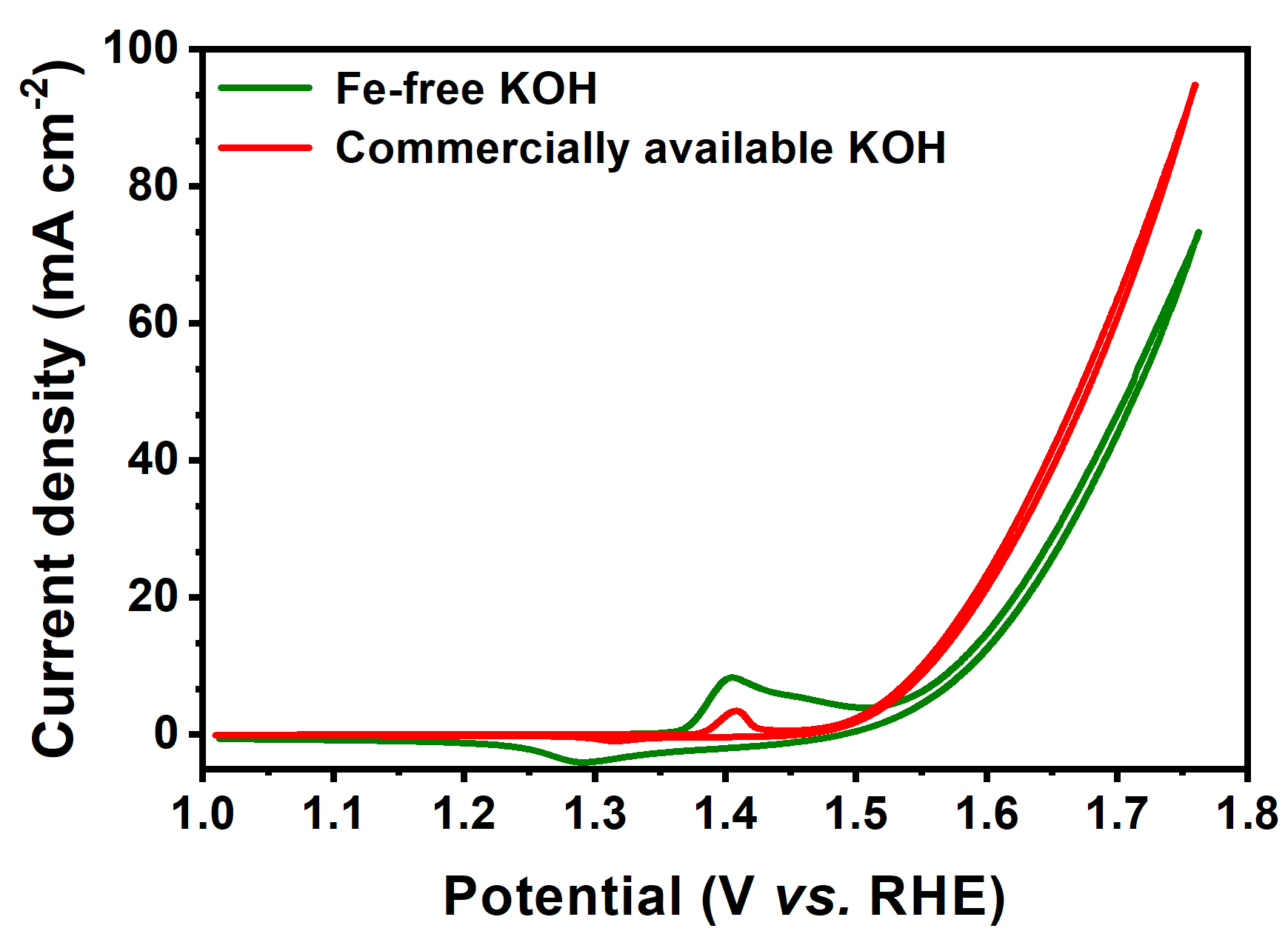


**Figure S21.** CV curves of Ba_8_Ni_6_Ge_40_/FTO recorded in commercially available 1 M KOH electrolyte and the Fe-free one which was purified using the same method reported in our previous work.^[S18]^

**
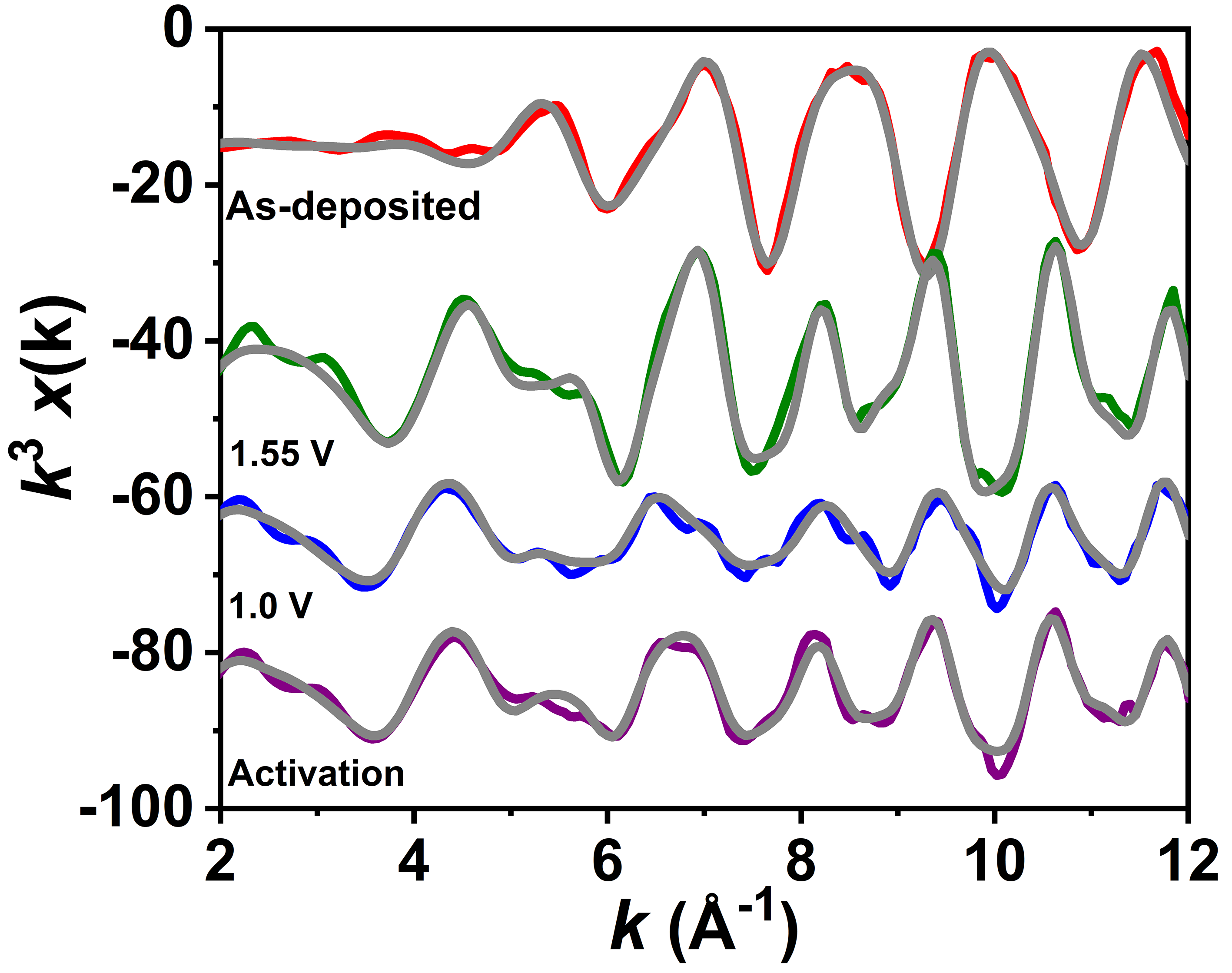
**

**Figure S22.** The *k*^3^-weighted EXAFS spectra of Ni K-edge for the *ex-situ* sample (as-deposited Ba_8_Ni_6_Ge_40_), as well as quasi *in-situ* ones (1.55 V, 1.0 V, and Activation). The grey lines represent the fitted data. The EXAFS simulation parameters (fit results) are shown in Table S5.

**Table S5.** Structural parameters for Ba_8_Ni_6_Ge_40_/FTO electrode at various states obtained from Ni *K*-edge EXAFS fitting.

| Sample | Path | *R* (Å) | *σ* (Å) | *N* | *R*_f_ |
| --- | --- | --- | --- | --- | --- |
| As-deposited | Ni-Ge | 2.29±0.01 | 0.063±0.001 | 4 | 14.5 |
|  | Ni-Ba | 3.90±0.04 | 0.063±0.001 | 2 |  |
|  | Ni-Ge | 3.97±0.02 | 0.063±0.001 | 6 |  |
| 1.55 V | Ni-O | 1.88±0.01 | 0.050 | 4.6±0.2 | 14.2 |
|  | Ni-Ni | 2.83±0.01 | 0.050 | 4.7±0.1 |  |
|  | Ni-O | 3.39±0.02 | 0.050 | 2.6±0.8 |  |
|  | Ni-Ni | 5.50±0.01 | 0.050 | 7.1±0.8 |  |
| 1.0 V | Ni-O | 1.87±0.01 | 0.050 | 2.4±0.2 | 18.7 |
|  | Ni-O | 2.04±0.01 | 0.050 | 2.8±0.3 |  |
|  | Ni-Ni | 2.84±0.01 | 0.050 | 2.3±0.3 |  |
|  | Ni-Ni | 3.06±0.01 | 0.050 | 1.5±0.4 |  |
|  | Ni-O | 3.44±0.03 | 0.050 | 2.9±1.0 |  |
|  | Ni-Ni | 5.30±0.03 | 0.050 | 2.4±1.3 |  |
|  | Ni-Ni | 5.50±0.02 | 0.050 | 3.4±1.5 |  |
| Activation | Ni-O | 1.88±0.01 | 0.050 | 3.1±0.2 | 19.4 |
|  | Ni-O | 2.06±0.02 | 0.050 | 2.0±0.3 |  |
|  | Ni-Ni | 2.84±0.01 | 0.050 | 3.5±0.3 |  |
|  | Ni-Ni | 3.03±0.02 | 0.050 | 1.1±0.4 |  |
|  | Ni-O | 3.45±0.03 | 0.050 | 3.2±0.9 |  |
|  | Ni-Ni | 5.29±0.04 | 0.050 | 1.5±1.2 |  |
|  | Ni-Ni | 5.50±0.02 | 0.050 | 4.3±1.4 |  |

*Note:* The range of the fits was 2~12 Å^–1^. *R* is the absorber-backscatter distance, *N* means the EXAFS coordination number, *σ* represents the Debye Waller factor. The *R*_f_ represents the mean fit error in %. The amplitude reduction factor, *S*_0_^2^ (k), was 0.9 in all refinements. The uncertainty ranges of the fit parameters represent the 68% confidence interval of the respective fit parameter. For the as-deposited sample: *N* was constrained (fixed) to represent the XRD structure, *σ* was restrained to be the same for all shells; for the OER samples: *σ* was constrained to a reasonable value to minimize the number of variable simulation parameters.


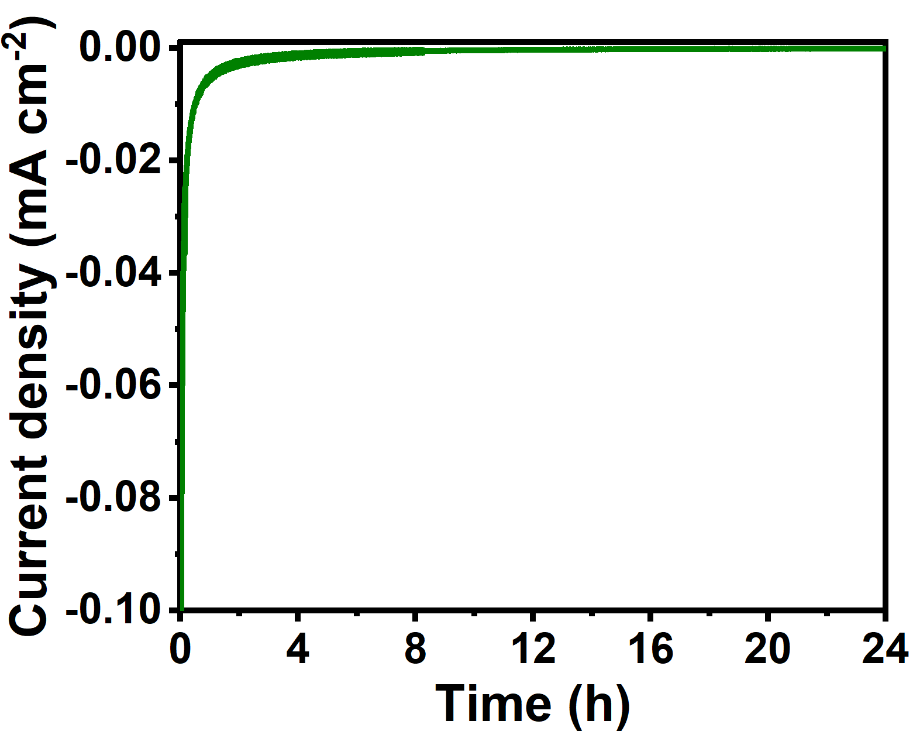


**Figure S23.** CA of the sample 1.55 V run under 1.0 V *vs.* RHE for 24 h.


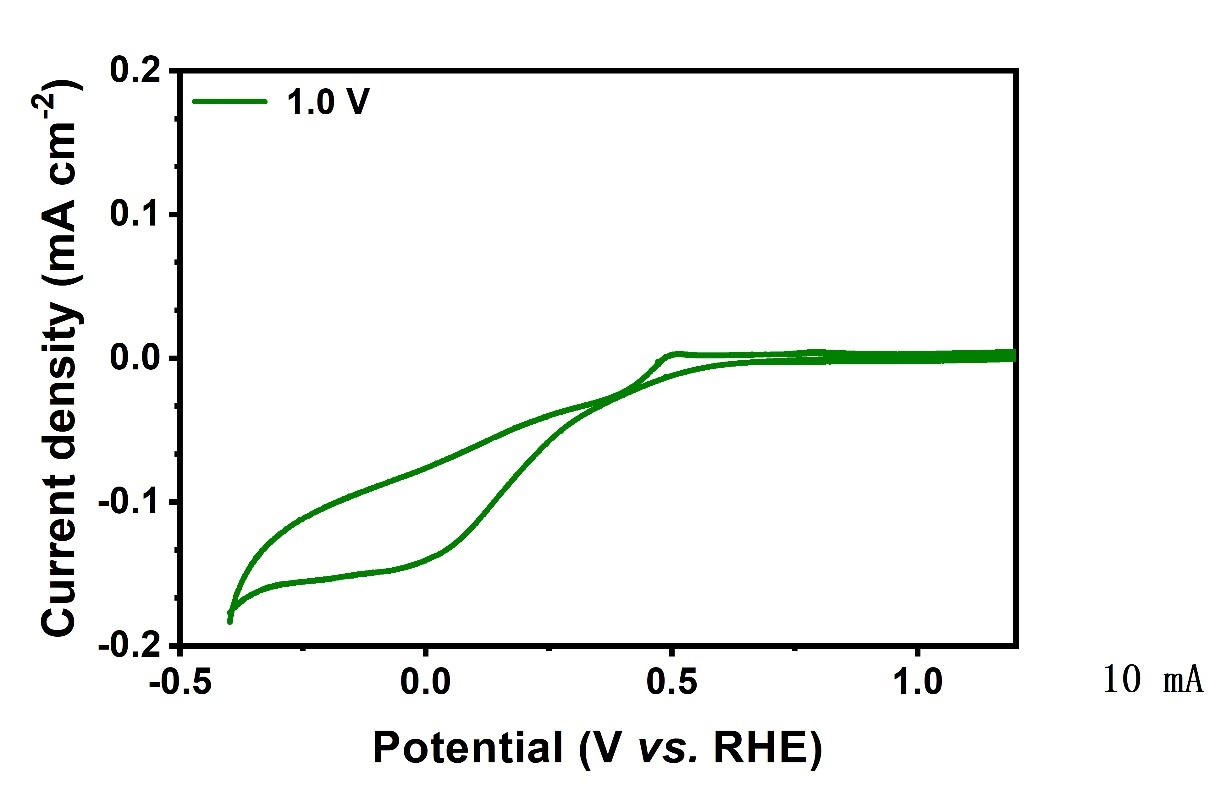


**Figure S24.** CV curve at a scan rate of 5 mV s^–1^ for the sample 1.0 V in 1 M KOH aqueous electrolyte.


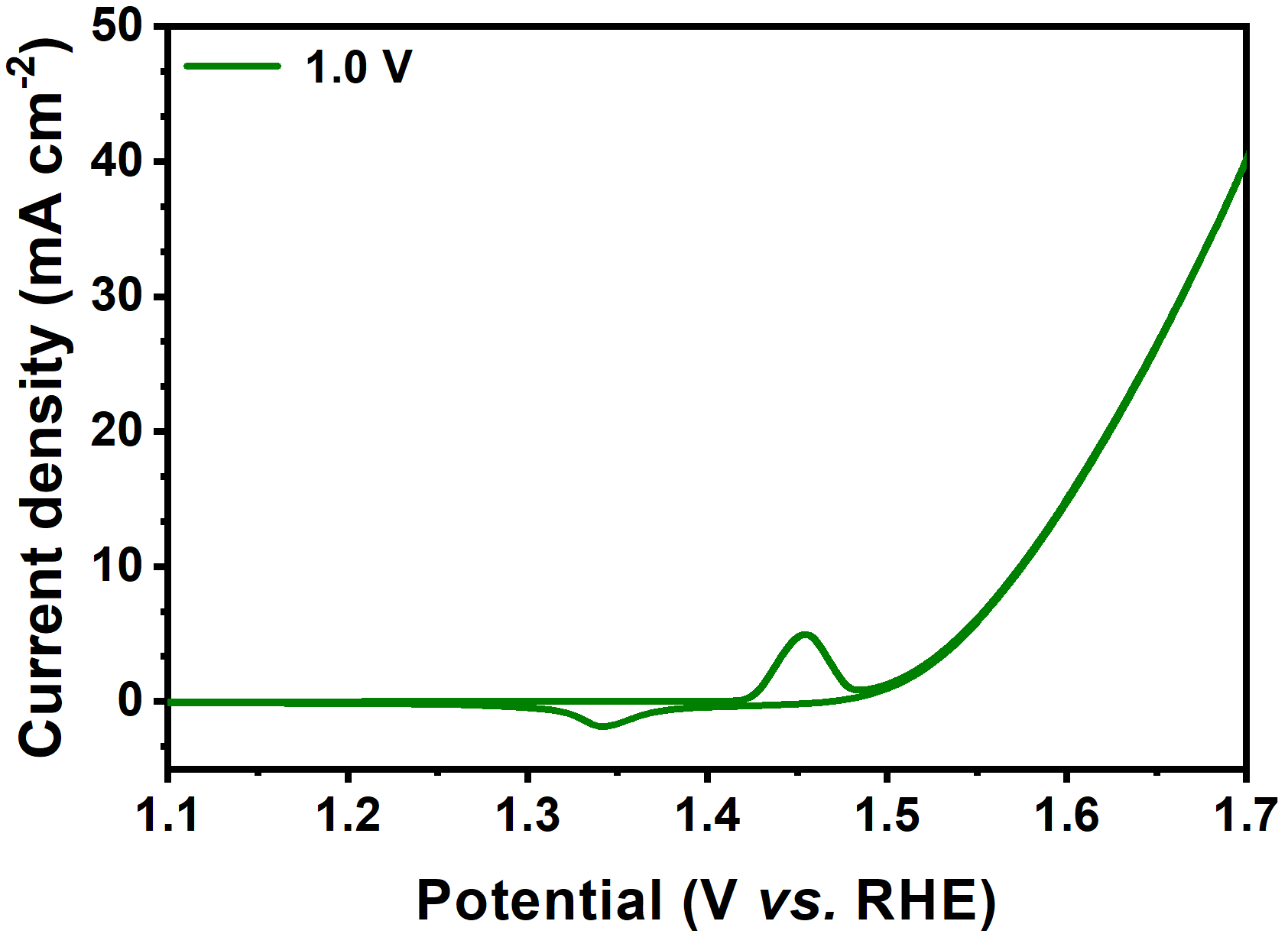


**Figure S25.** CV curve of Ba_8_Ni_6_Ge_40_/FTO after 24 h CA at 1.55 V *vs.* RHE and subsequently 24 h CA at 1.0 V *vs.* RHE (the sample “1.0 V”).


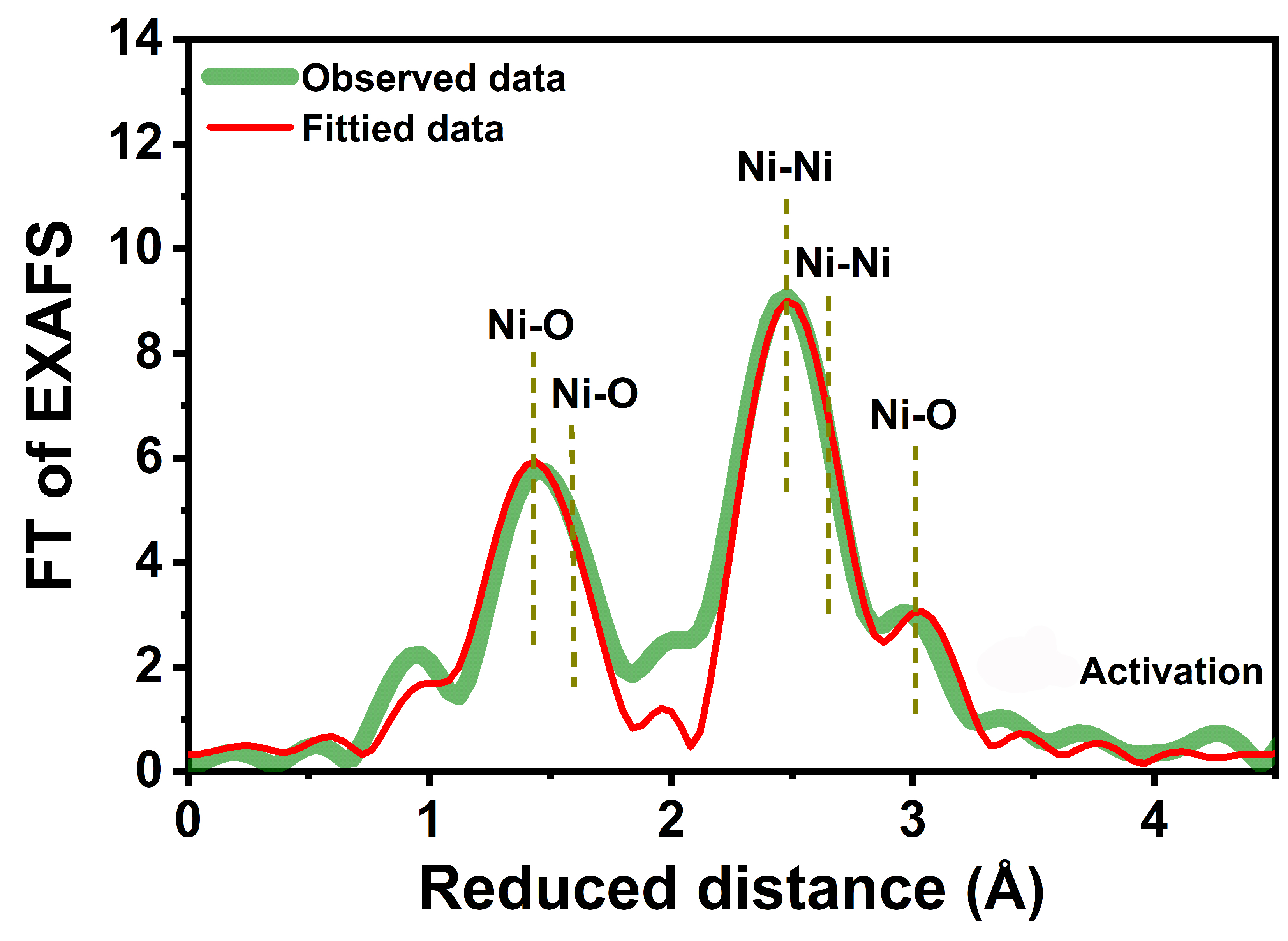


**Figure S26.** Fitted FT-EXAFS spectra of Ni *K*-edge in Ba_8_Ni_6_Ge_40_/FTO after OER activation in 1.0 M KOH electrolyte. The EXAFS simulation parameters (fit results) are shown in Table S5.


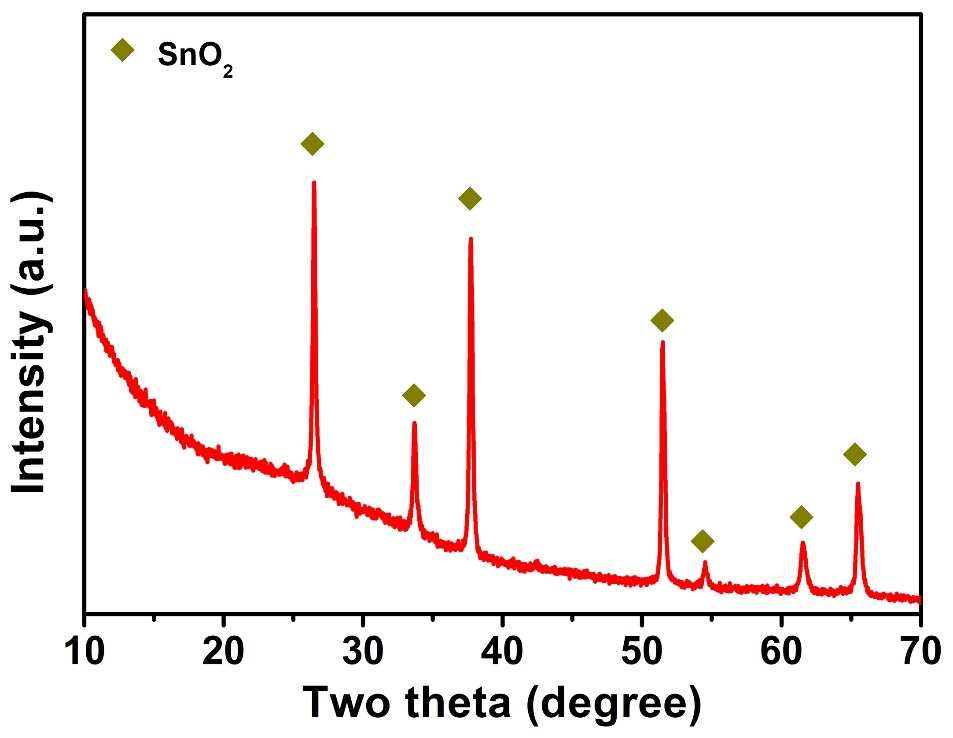


**Figure S27.** PXRD pattern of Ba_8_Ni_6_Ge_40_/FTO electrode after OER activation in 1.0 M KOH electrolyte showing the deep OER-driven reconstruction.

**
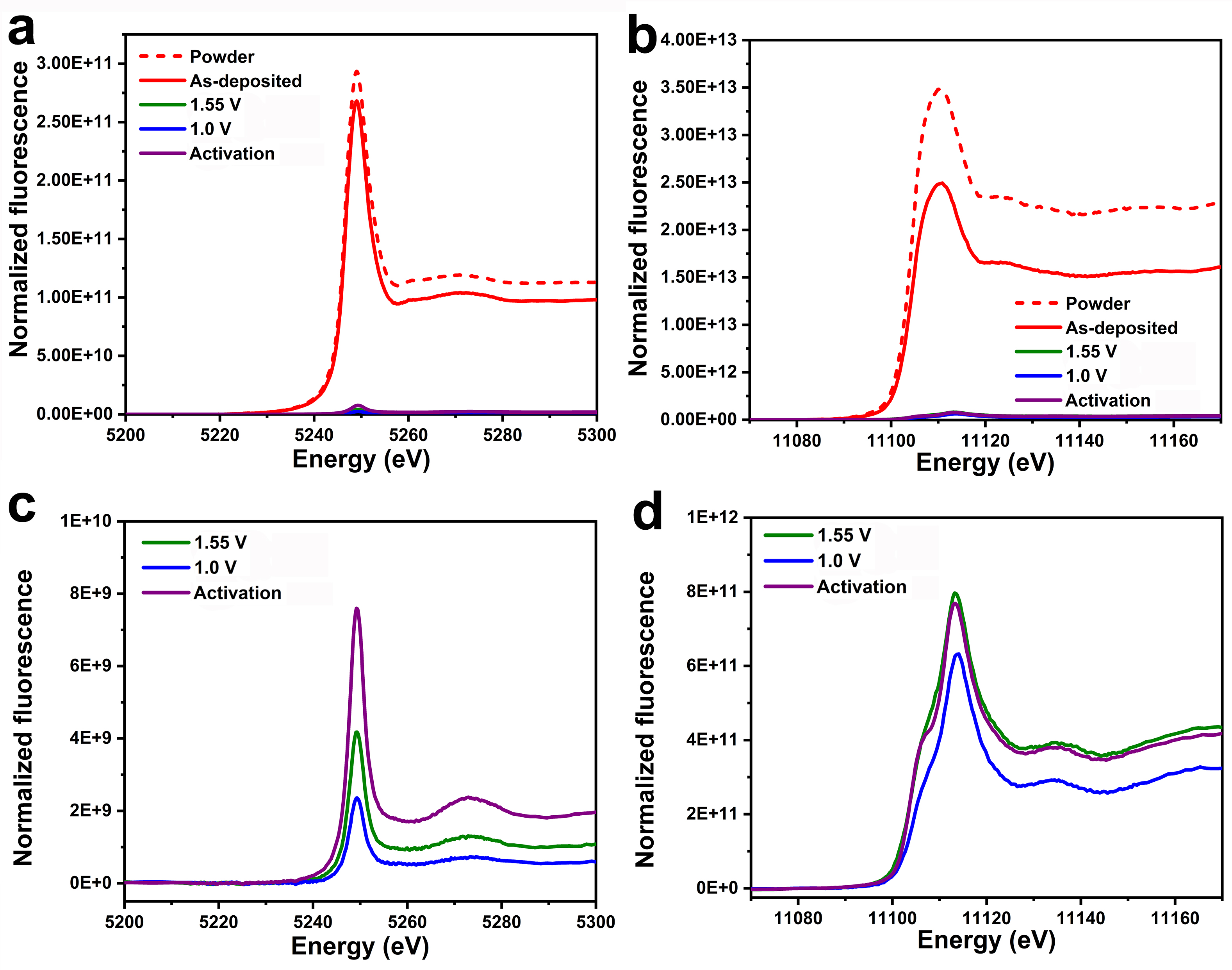
**

**Figure S28.** The raw XAS spectra of (a) Ba and (b) Ge for the *ex-situ* samples (as-prepared and as-deposited Ba_8_Ni_6_Ge_40_) and quasi *in-situ* ones (1.55 V, 1.0 V, and Activation). The corresponding magnification of the raw XAS spectra of (c) Ba and (d) Ge for the three quasi *in-situ* samples (1.55 V, 1.0 V, and Activation).

**
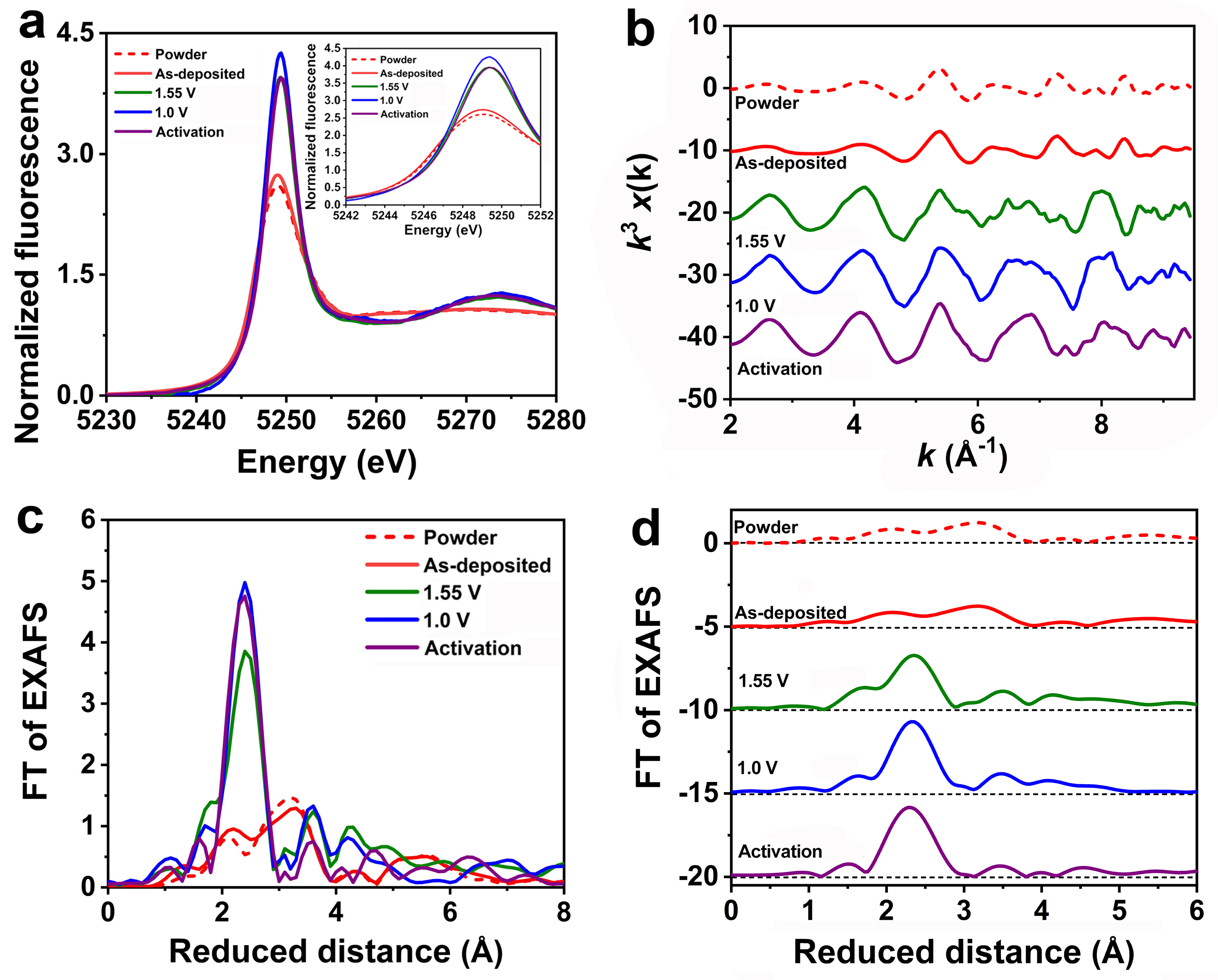
**

**Figure S29.** (a) XANES spectra (inset: magnified area), (b) *k*^3^-weighted EXAFS spectra, (c, d) FT-EXAFS spectra of Ba L-edge for the *ex-situ* samples (as-prepared and as-deposited Ba_8_Ni_6_Ge_40_) and quasi *in-situ* ones (1.55 V, 1.0 V, and Activation). Due to the small EXAFS amplitudes and low spectral resolution, EXAFS was not fitted for Ba.

**
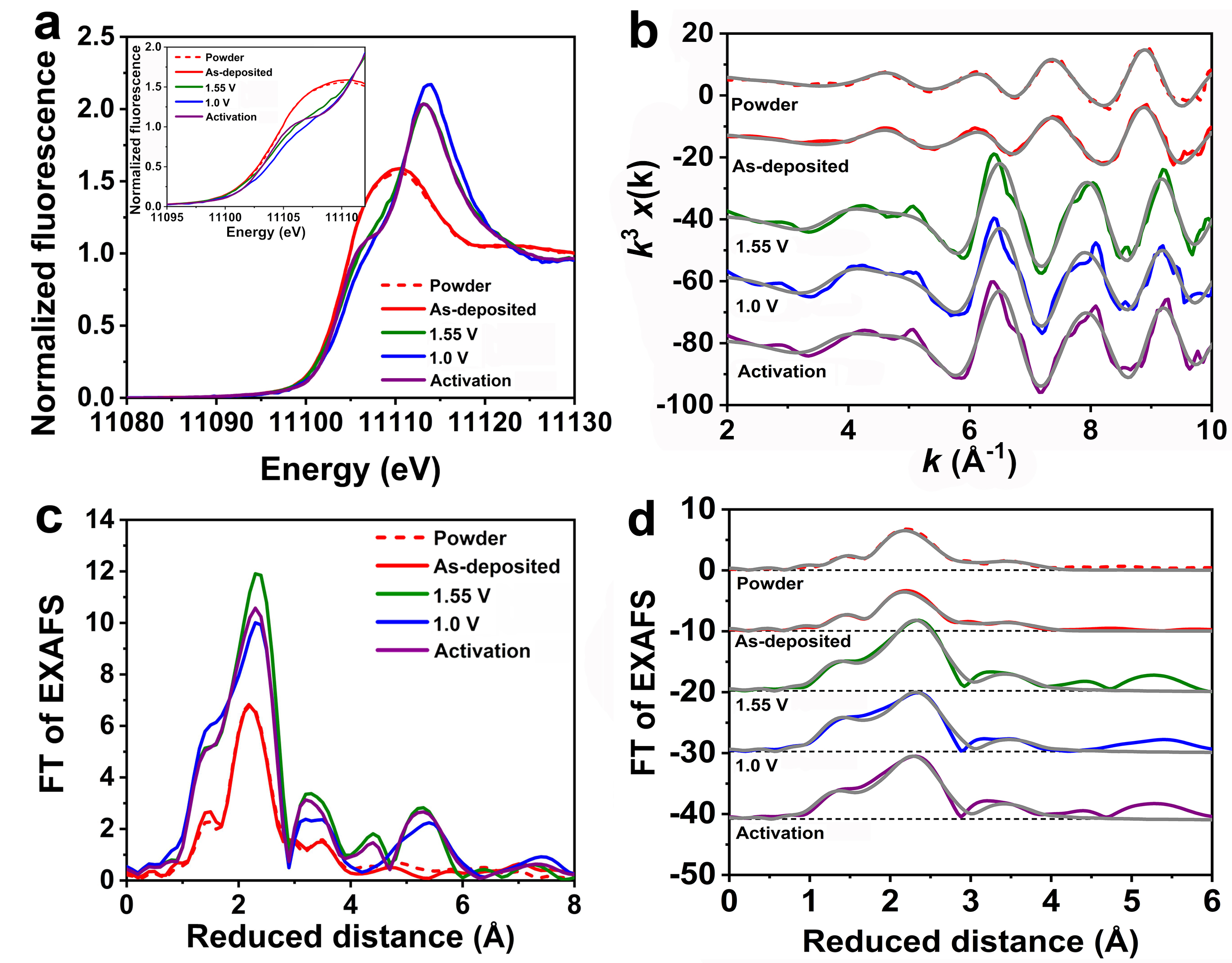
**

**Figure S30.** (a) XANES spectra (inset: magnified area), (b) *k*^3^-weighted EXAFS spectra, (c, d) FT-EXAFS spectra of Ge K-edge for the *ex-situ* samples (as-prepared and as-deposited Ba_8_Ni_6_Ge_40_) and quasi *in-situ* ones (1.55 V, 1.0 V, and Activation). The grey lines shown in Figure b and d represent the fitted data.

**Table S6.** Structural parameters for as-prepared Ba_8_Ni_6_Ge_40_ powder and Ba_8_Ni_6_Ge_40_/FTO electrode at various states obtained from Ge *K*-edge EXAFS fitting.

| Sample | Path | *R* (Å) | *σ* (Å) | *N* | *R*_f_ |
| --- | --- | --- | --- | --- | --- |
| Powder | Ge-O | 1.77±0.02 | 0.050 | 0.7±0.2 | 10.2 |
|  | Ge-Ge | 2.52±0.01 | 0.050 | 2.1±0.1 |  |
|  | Ge-Ge | 3.94±0.02 | 0.050 | 1.3±0.4 |  |
| As-deposited | Ge-O | 1.78±0.02 | 0.050 | 0.8±0.2 | 9.2 |
|  | Ge-Ge | 2.52±0.01 | 0.050 | 2.1±0.1 |  |
|  | Ge-Ge | 3.95±0.02 | 0.050 | 1.2±0.4 |  |
| 1.55 V | Ge-O | 1.83±0.02 | 0.050 | 2.2±0.2 | 13.3 |
|  | Ge-Ge | 2.33±0.01 | 0.050 | 2.2±0.2 |  |
|  | Ge-Ge | 2.80±0.01 | 0.050 | 4.0±0.3 |  |
|  | Ge-Ge | 3.80±0.01 | 0.050 | 2.2±0.4 |  |
| 1.0 V | Ge-O | 1.84±0.02 | 0.050 | 2.5±0.2 | 18.0 |
|  | Ge-Ge | 2.34±0.01 | 0.050 | 2.2±0.1 |  |
|  | Ge-Ge | 2.81±0.01 | 0.050 | 3.4±0.3 |  |
|  | Ge-Ge | 3.78±0.02 | 0.050 | 1.8±0.4 |  |
| Activation | Ge-O | 1.81±0.02 | 0.050 | 2.1±0.2 | 15.9 |
|  | Ge-Ge | 2.32±0.01 | 0.050 | 2.2±0.1 |  |
|  | Ge-Ge | 2.79±0.01 | 0.050 | 3.5±0.3 |  |
|  | Ge-Ge | 3.77±0.02 | 0.050 | 2.1±0.4 |  |

*Note:* The range of the fits was 2~10 Å^–1^. *R* is the absorber-backscatter distance, *N* means the EXAFS coordination number, *σ* represents the Debye Waller factor. The *R*_f_ represents the fit error sum in %. The amplitude reduction factor, *S*_0_^2^ (k), was 1.0 in all refinements. The errors represent the 68% confidence interval of the respective fit parameter. *σ* was constrained to a reasonable value to to minimize the number of variable simulation parameters.


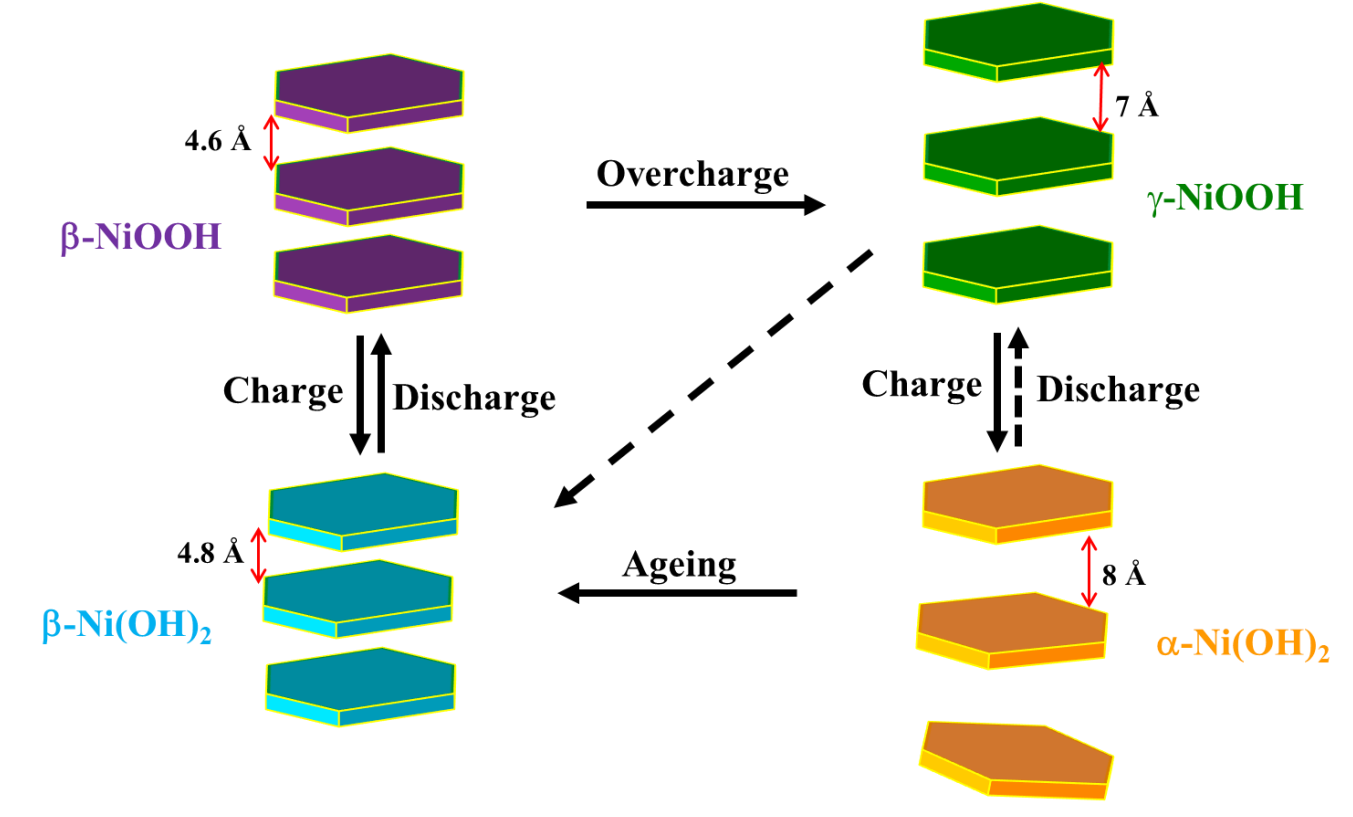


**Figure S31.** Bode’s diagram representing different variation of nickel (oxy)hydroxides and possible phase transformation within them.^[S8]^


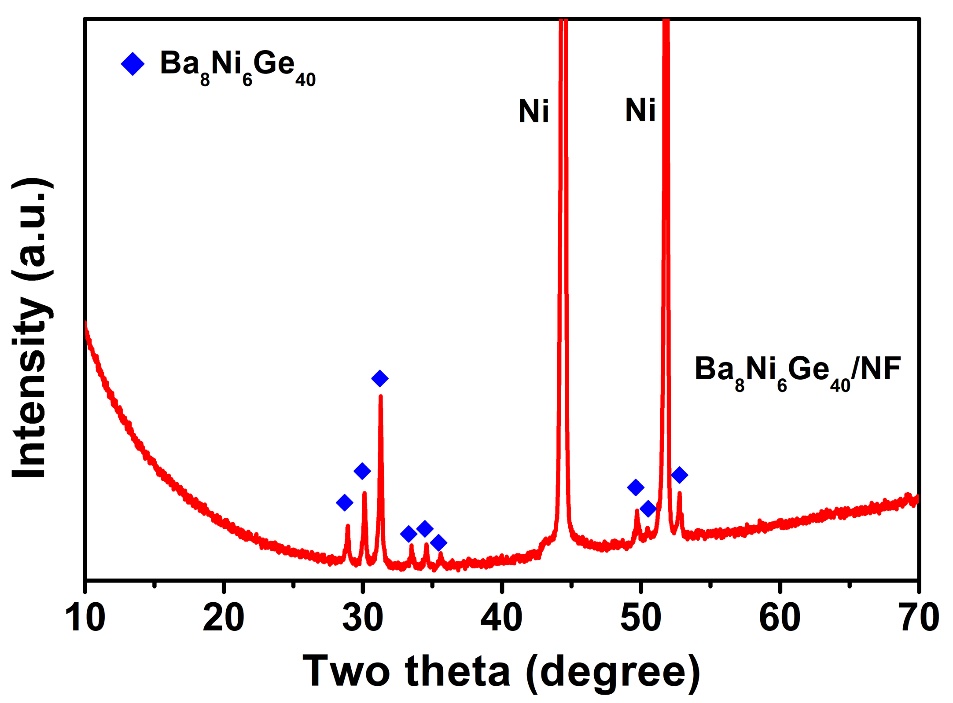


**Figure S32.** The PXRD pattern of the as-deposited Ba_8_Ni_6_Ge_40_/NF electrode showing that Ba_8_Ni_6_Ge_40_ was successfully supported on NF substrate.


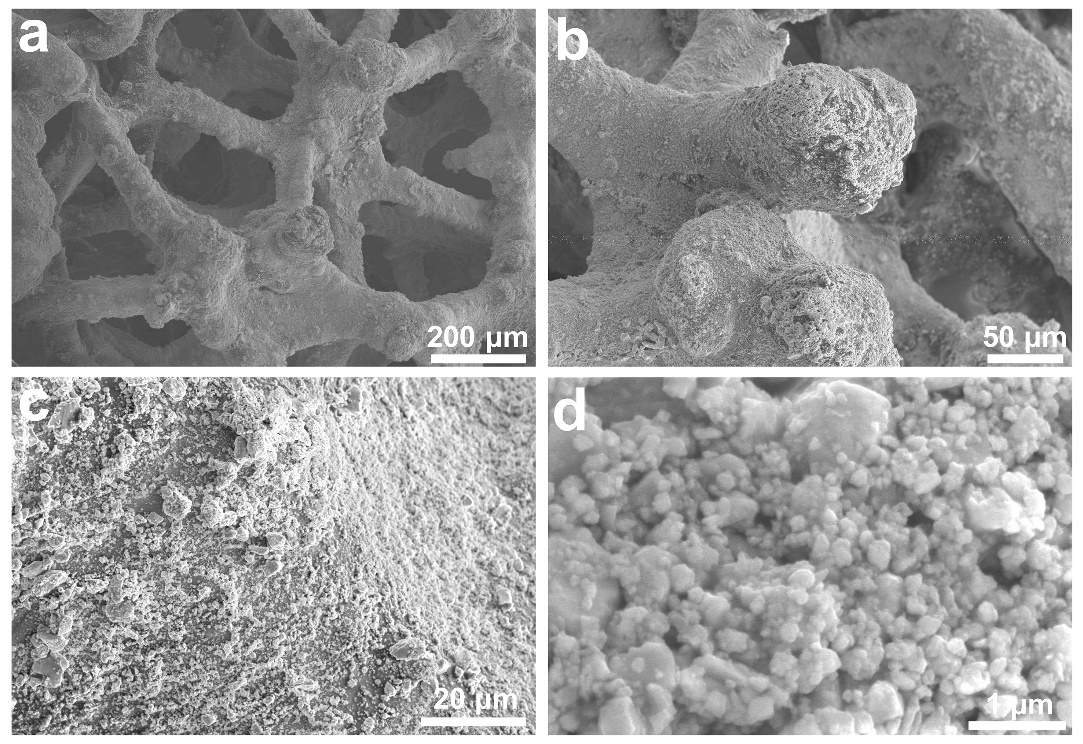


**Figure S33.** (a–d) SEM images of the as-deposited Ba_8_Ni_6_Ge_40_/NF electrode.


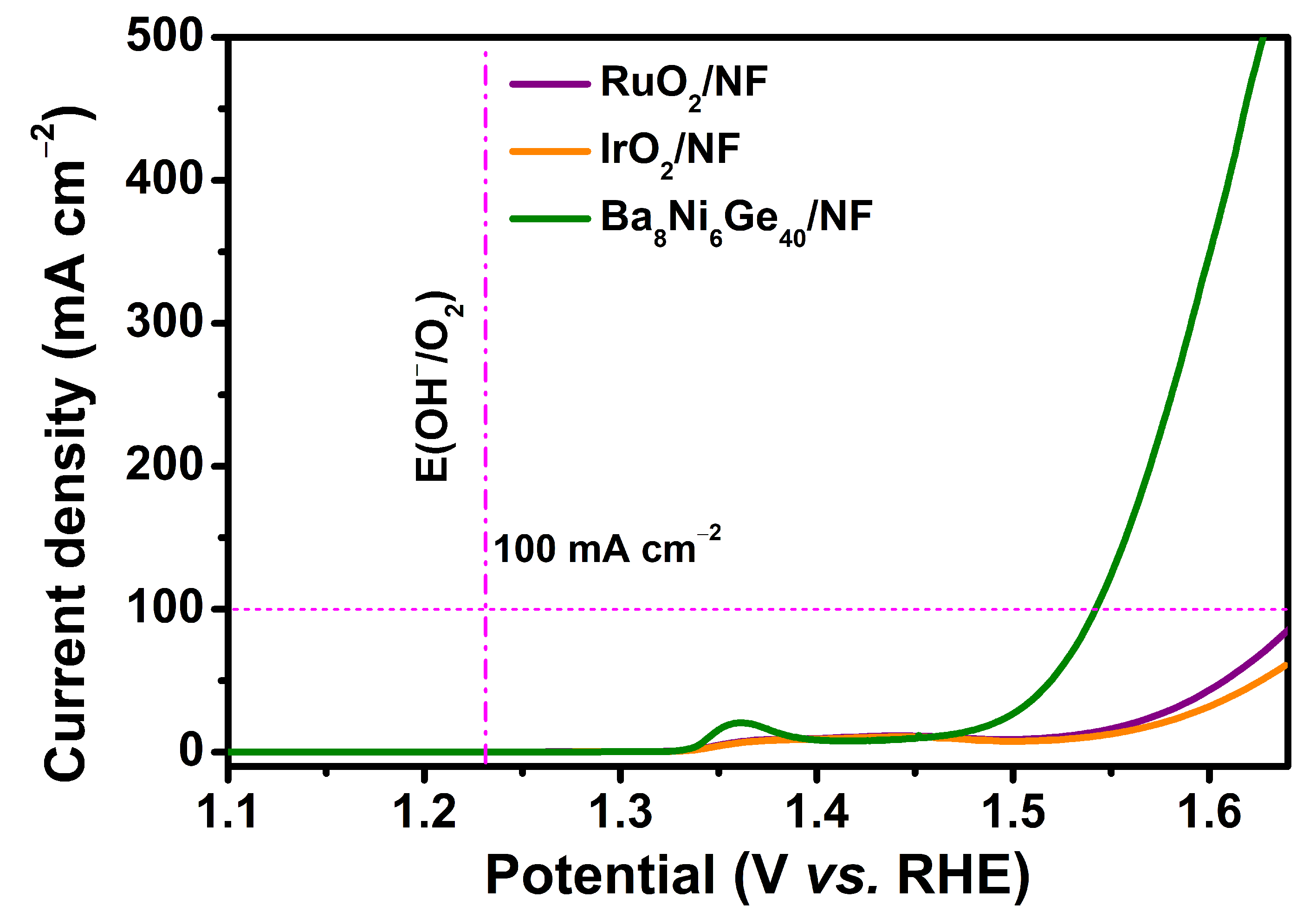


**Figure S34.** LSV curves (5 mV s^−1^) of RuO_2_/NF and IrO_2_/NF with the same mass loading as that used for the Ba_8_Ni_6_Ge_40_/NF.


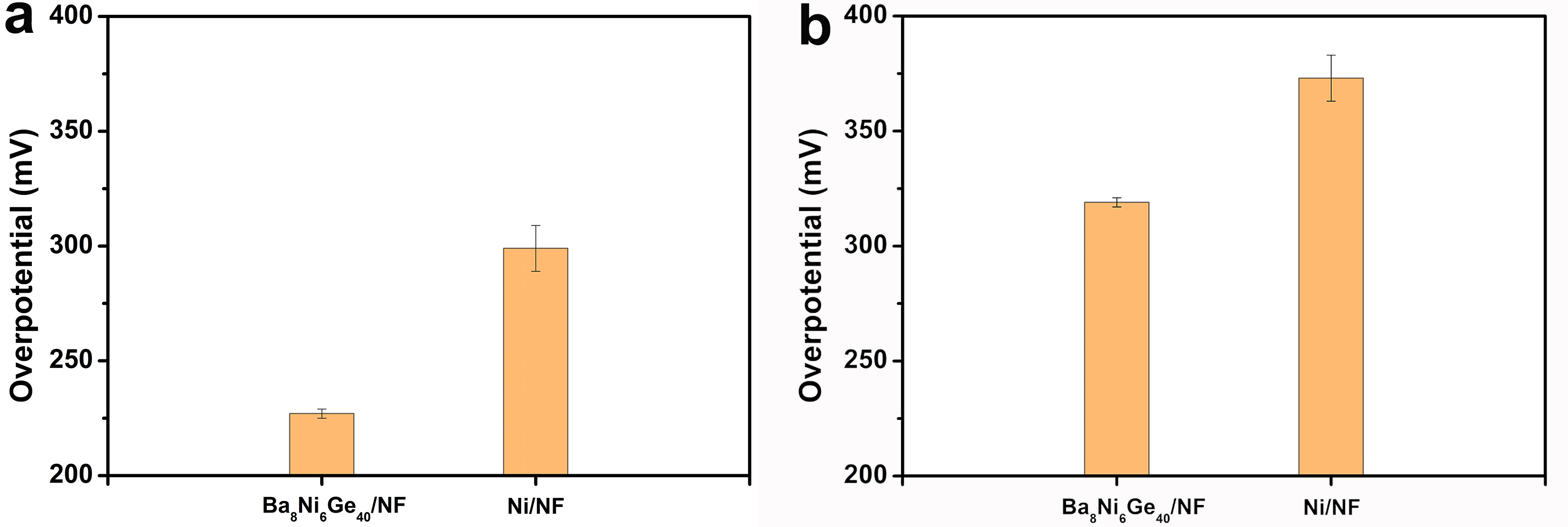


**Figure S35.** The mean values of the overpotentials with standard deviation at (a) 10 mA cm^–2^ and (b) 100 mA cm^–2^ for Ni/NF and Ba_8_Ni_6_Ge_40_/NF based on three independent CV measurements (the values of the forward-scanned curves were used).

**Table S7.** The comparison of OER activity and stability of Ba_8_Ni_6_Ge_40_/NF with those of recently reported NF-supported intermetallics- and Ni-based OER catalysts in 1 M KOH electrolyte.

| **Electrode** | **Loading (mg cm^−2^)** | **Temperature** | **Overpotential @ 10 mA cm^–2^** | **Stability**  ***j* (mA cm^–2^) @ *t* (h)** | **Ref.** |  |
| --- | --- | --- | --- | --- | --- | --- |
| CaFe_6_Ge_6_/NF | ~3.5 | R.T.^a^ | 264 mV | 1000@25 | [S7b] |  |
| NiGe/NF | ~1±0.08 | R.T. | 228±3 mV | 10@505 | [S9] |  |
| FeAs/NF | 1.0 ±0.1 | R.T. | 252±3 mV | 10@24 | [S14] |  |
| Ni_12_P_5_/NF | | ~3 | R.T. | 240 mV | 10@11* | [S15] |
| Ni/NiFeMoO*_x_*/NF | 1.8 | R.T. | 255 mV | 500@100* | [S27] |  |
| MnGa_4_/NF | ~2 | R.T. | 293 mV | 15@120 | [S28] |  |
| MoNi_4_/NF | ~1.09 | 25 °C | 280 mV | 10@24 | [S29] |  |
| MoFeNi/NF | 1.6 | 25 °C | 238 mV | 25@50* | [S30] |  |
| LaNi_5_-H/NF | 3 | R.T. | 254 mV | ~560@240 | [S31] |  |
| NiCo@C-NiCoMoO*_x_*/NF | 10.5 | NA | 260 mV | 1000@340 | [S32] |  |
| NiCo/NF | 4 | NA | 320 mV | 50@15 | [S33] |  |
| NiFeSn/NF^b^ | NA | R.T. | 253 mV | 200@12 | [S34] |  |
| NiAl/NF | NA | R.T. | 251 mV | 10~30@12 | [S35] |  |
| N,C-FeCoNiMg/NF | NA | 25 | 235 mV | 1000@100 | [S36] |  |
| NiCo_2_S_4_/NF | NA | NA | 260 mV | 10@50 | [S37] |  |
| Ni@C/NF | 7.3 ± 0.3 | R.T. | 265 mV | 36@70 | [S38] |  |
| Ni-S-P-O/NF | ~4.8 | NA | 259 mV | 20@20 | [S39] |  |
| NiMoFe-O/NF | 18 | R.T. | 262 mV | \ | [S40] |  |
| NiFe-OOH/NF | -14^c^ | R.T. | 250 mV | \ | [S40] |  |
| Ag/NiCoMoO/ /NF | ~11.3 | 25 ± 2 °C | 243 mV | 10@70 | [S41] |  |
| NiS_0.5_Se_0.5_/NF | 2.4 | R.T. | 257 mV | 100@300 | [S42] |  |
| Ni_3_S_2_/NF | 1.6 | NA | 260 mV | 10@200 | [S43] |  |
| Ni(OH)_2_(CO_3_)_2_-Fe^2+^/NF | 0.4 | NA | 277 | 15.5@36 | [S44] |  |
| NiFe-(CO_3_)^2-^-LDH/NF | 3.2~4.5 | NA | 228 | 100@20 | [S45] |  |
| Ba_8_Ni_6_Ge_40_/NF | 2 | R.T. | 227 ± 2 | ~550@240^d^ | This work |  |

^a^ R.T. means the room temperature; ^b^ 30 wt.% KOH was used as the electrolyte; ^c^ The negative loading was caused by the galvanic replacement between Ni^0^ of the substrate surface and Fe^III^ ions from the solution;^[S40] d^ The stability was conducted by an assembled two-electrode alkaline water electrolyzer where Ba_8_Ni_6_Ge_40_/NF acted as both cathode and anode.

**
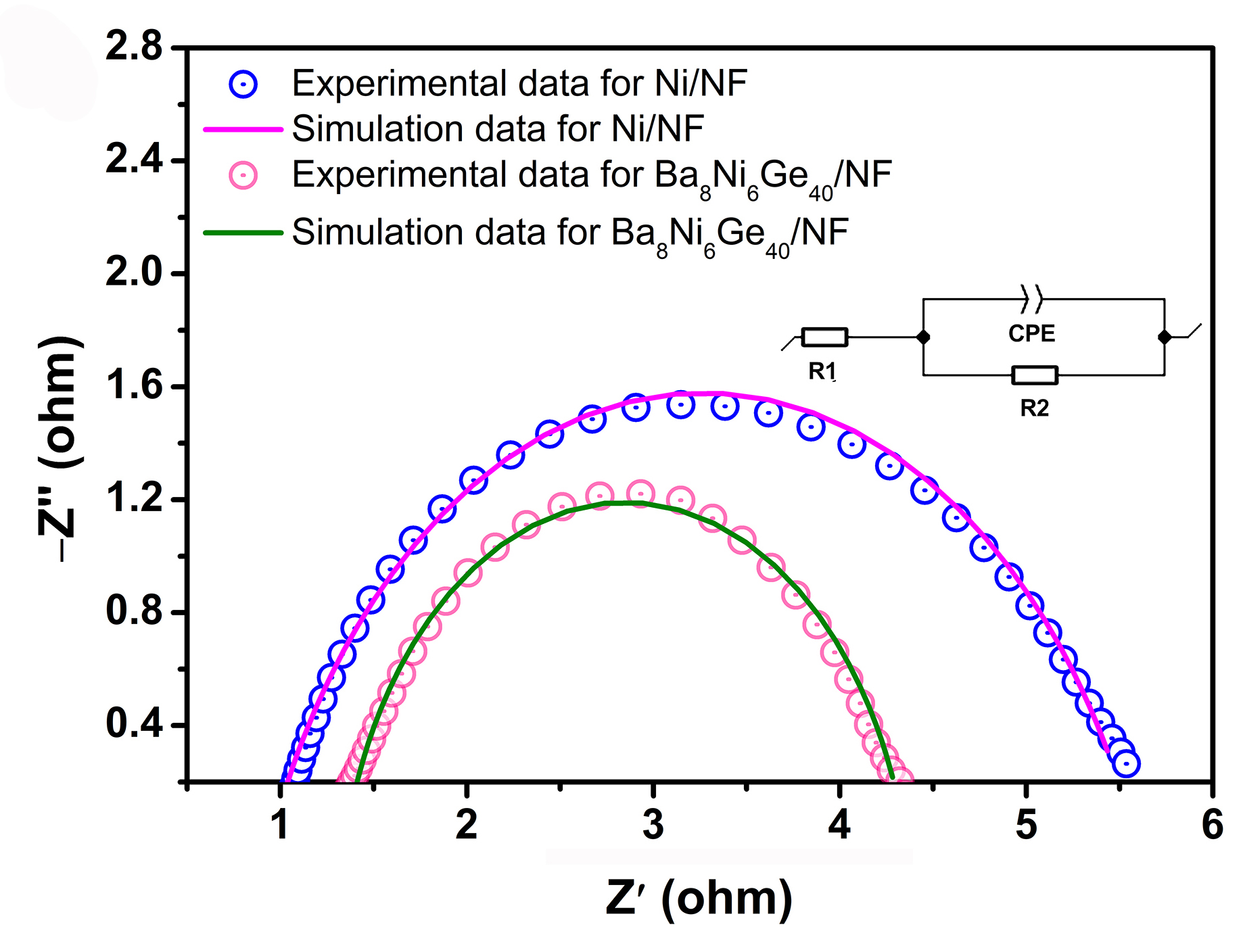
**

**Figure S36.** Comparison of EIS spectra between Ni/NF and Ba_8_Ni_6_Ge_40_/NF electrode at the potential of 1.55 V *vs*. RHE in 1.0 M KOH electrolyte. Note that the pre-activation was performed.

**
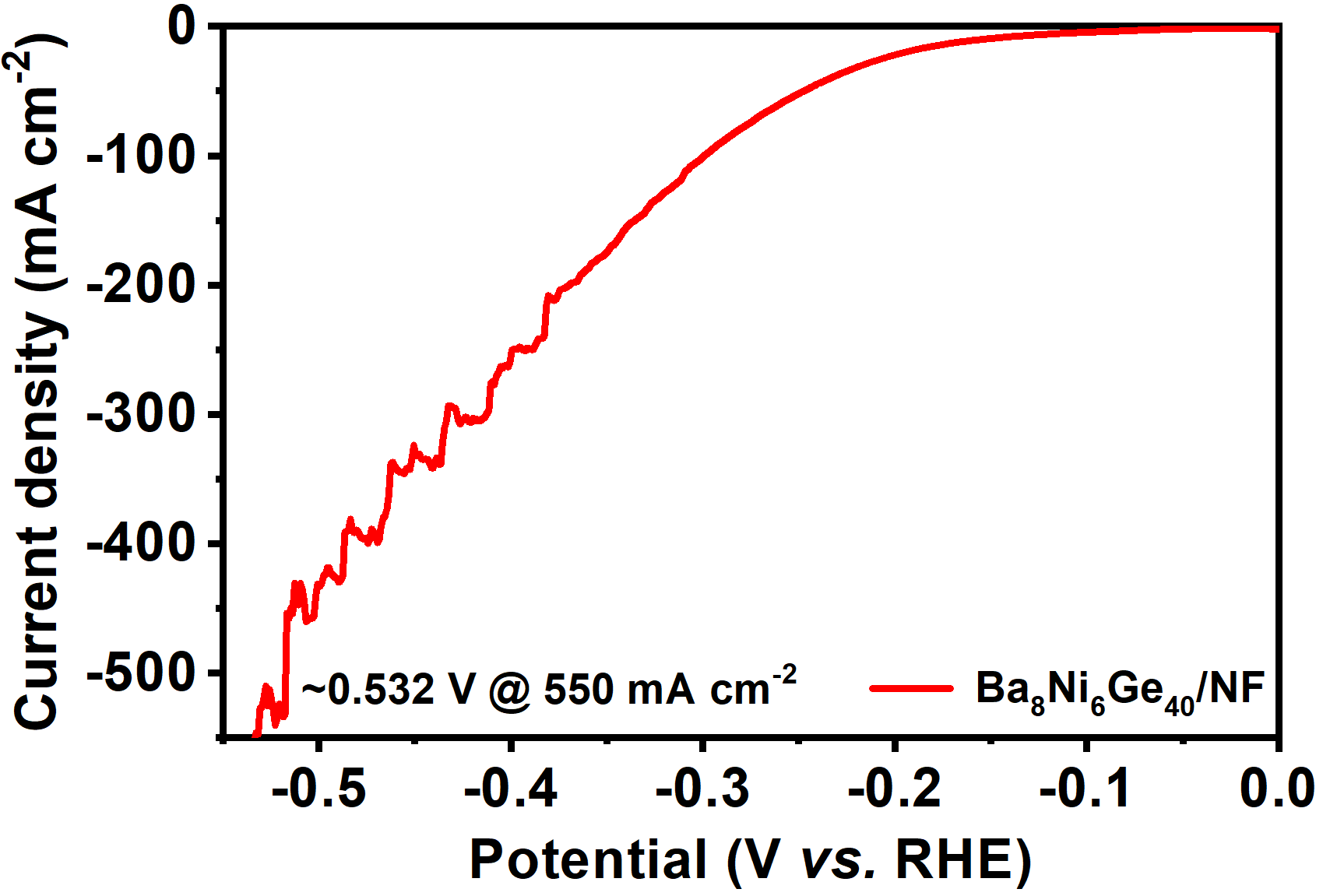
**

**Figure S37.** Hydrogen evolution reaction (HER) LSV curves (5 mV s^−1^) of Ba_8_Ni_6_Ge_40_/NF in 1.0 M KOH electrolyte.


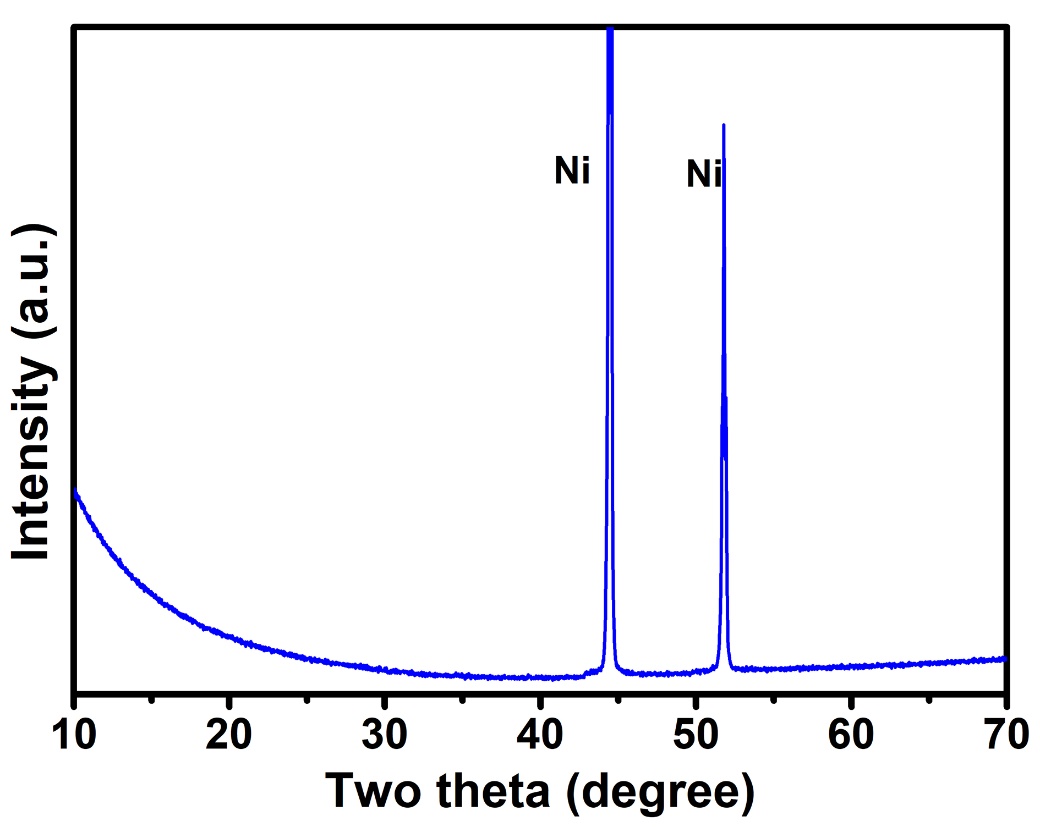


**Figure S38.** PXRD pattern of the as-deposited Ba_8_Ni_6_Ge_40_/NF electrode after OER CA showing that the deep structural reconstruction of Ba_8_Ni_6_Ge_40_ took place.


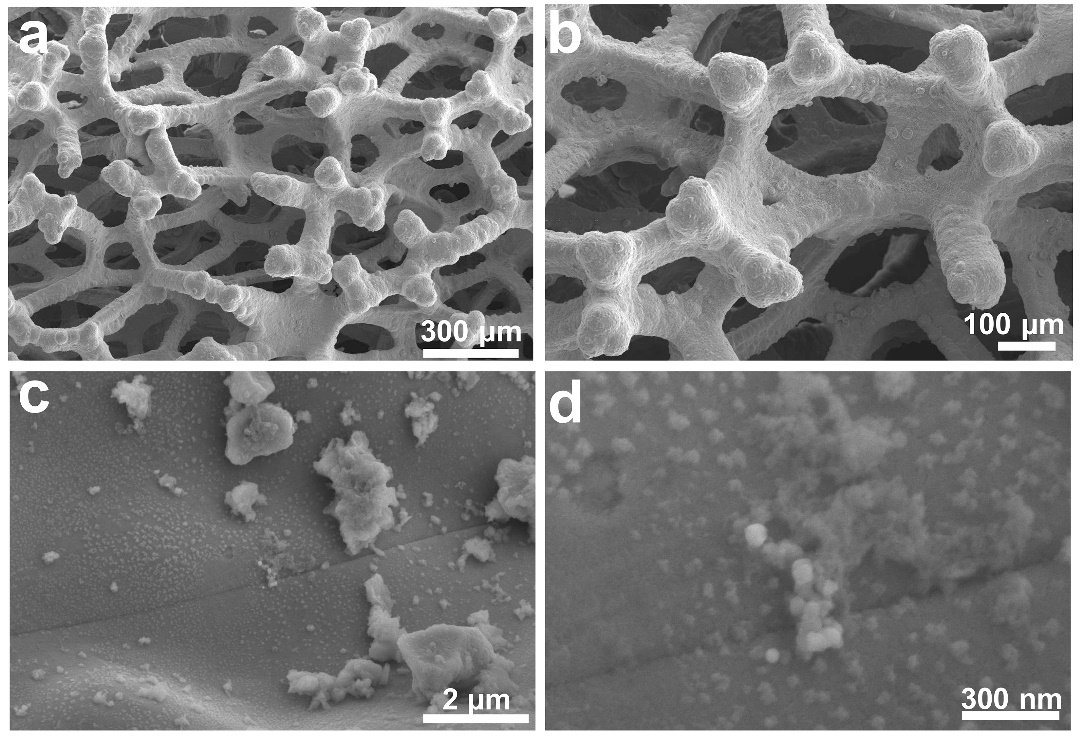


**Figure S39.** SEM images of Ba_8_Ni_6_Ge_40_/NF electrode after OER CA.


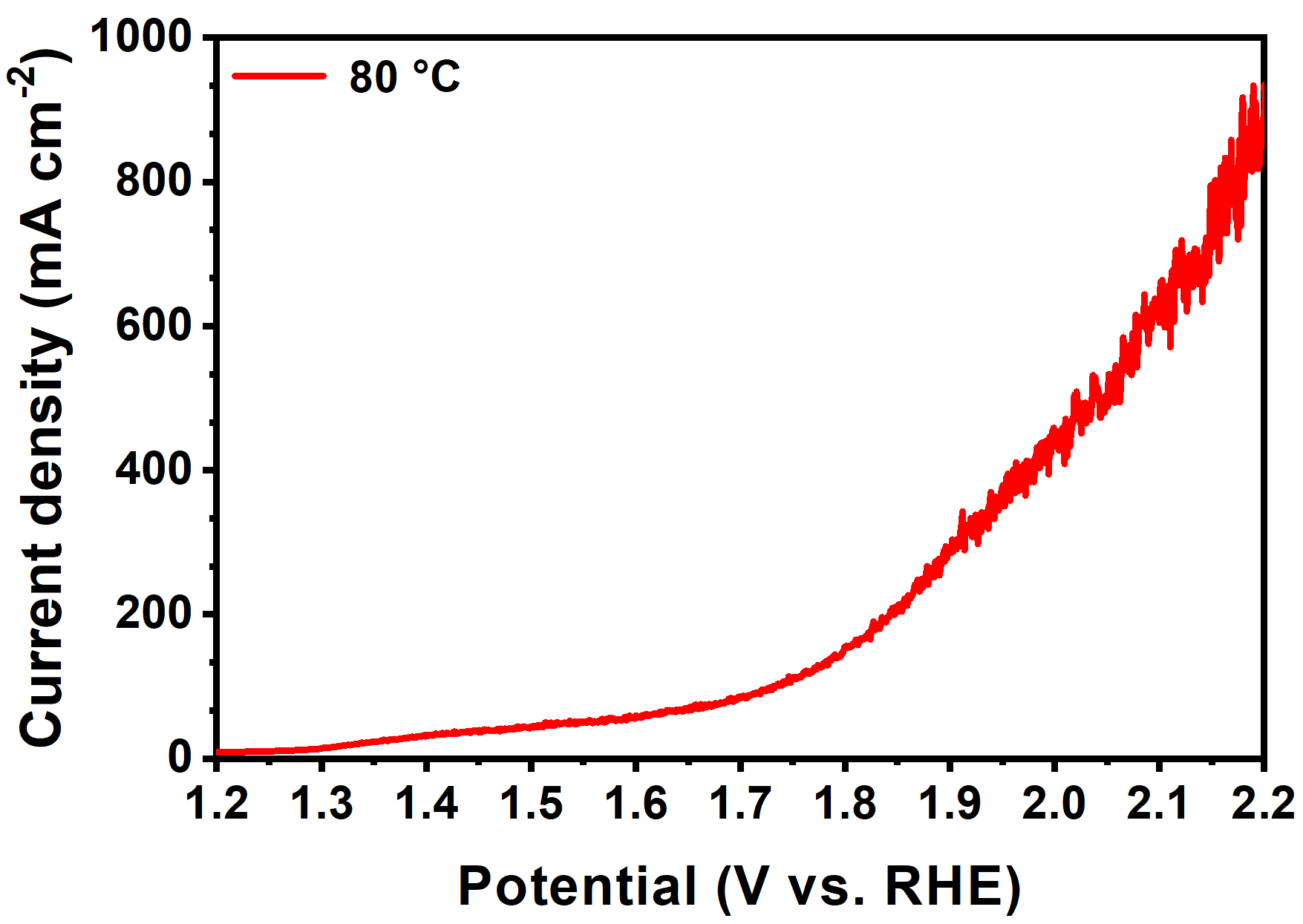


**Figure S40.** The LSV curve of the assembled membrane-free two-electrode electrolyzer using Ba_8_Ni_6_Ge_40_/NF as both cathode and anode at 80 °C.

**References**

[S1] J. N. Hausmann, R. Beltrán-Suito, S. Mebs, V. Hlukhyy, T. F. Fässler, H. Dau, M. Driess, P. W. Menezes, *Adv. Mater.* **2021**, *33*, 2008823.

[S2] F. Izumi, K. Momma, *Solid State Phenomena, Vol. 130*, Trans Tech Publ, **2007**, pp. 15−20.

[S3] K. Momma, F. Izumi, *J. Appl. Crystallogr.* **2011**, *44*, 1272−1276.

[S4] B. Ravel, M. Newville, *J. Synchrotron Radiat.* **2005**, *12*, 537−541.

[S5] a) L. Besra, M. Liu, *Prog. Mater. Sci.* **2007**, *52*, 1-61; b) F. Bozza, R. Polini, E. Traversa, *Fuel Cells* **2008**, *8*, 344−350; c) P. W. Menezes, C. Panda, S. Garai, C. Walter, A. Guiet, M. Driess, *Angew. Chem. Int. Ed* **2018**, *130*, 15457−15462; d) J. Pfrommer, A. Azarpira, A. Steigert, K. Olech, P. W. Menezes, R. F. Duarte, X. Liao, R. G. Wilks, M. Bär, T. Schedel‐Niedrig, *ChemCatChem* **2017**, *9*, 672−676; e) Y. Yang, J. Huang, J. Zeng, J. Xiong, J. Zhao, *ACS Appl. Mater. & Inter.* **2017**, *9*, 32801−32811; f) Y. Yang, J. Li, D. Chen, T. Fu, D. Sun, J. Zhao, *ChemElectroChem* **2016**, *3*, 757−763.

[S6] S. Anantharaj, S. Noda, M, Driess, P, W. Menezes, *ACS Energy Lett.* **2021**, *6*, 1607–1611.

[S7] a) S. Anantharaj, S. R. Ede, K. Karthick, S. S. Sankar, K. Sangeetha, P. E. Karthik, S. Kundu, *Energy Environ. Sci.* **2018**, *11*, 744; b) H. Yang, J. N. Hausmann, V. Hlukhyy, T. Braun, K. Laun, I. Zebger, M. Driess, P. W. Menezes, *ChemCatChem* **2022**, *14*, e202200293.

[S8] a) S. Jahangiri, N. J. Mosey, *Phy. Chem. Chem. Phys.* **2018**, *20*, 11444−11453; b) A. Van der Ven, D. Morgan, Y. S. Meng, G. Ceder, *J. Electrochem. Soc.* **2006**, *153*, A210−A215; c) S. Lee, I. Kim, H. Cho, C. Kim, Y. Lee, *Appl. Catal. B* **2021**, *284*, 119729.

[S9] P. W. Menezes, S. Yao, R. Beltrán-Suito, J. N. Hausmann, P. V. Menezes, M. Driess, *Angew. Chem. Int. Ed* **2021**, *60*, 4640−4647.

[S10] K. Zhu, W. Luo, G. Zhu, J. Wang, Y. Zhu, Z. Zou, W. Huang, *Chem. Asian J.* **2017**, *12*, 2720.

[S11] M. A. Schoen, O. Calderon, N. M. Randell, S. Jimenez-Villegas, K. M. Daly, R. Chernikov, S. Trudel, *J. Mater. Chem. A* **2021**, *9*, 13252.

[S12] M. Wang, Z. Dang, M. Prato, D. V. Shinde, L. De Trizio, L. Manna, *ACS Appl. Nano Mater.* **2018**, *1*, 5753

[S13] S. Manzoor, T. Munawar, S. Gouadria, M. Sadaqat, A. G. Abid, A. Munawar, F. Hussain, F. Iqbal, I. Ahmad, M. N. Ashiq, *J. Energy Storage* **2022**, *55*, 105488.

[S14] R. Beltrán-Suito, V. Forstner, J. N. Hausmann, S. Mebs, J. Schmidt, I. Zaharieva, K. Laun, I. Zebger, H. Dau, P. W. Menezes, *Chem. Sci.* **2020**, *11*, 11834.

[S15] P. W. Menezes, A. Indra, C. Das, C. Walter, C. Göbel, V. Gutkin, D. Schmeiβer, M. Driess, *ACS Catal.* **2017**, *7*, 103.

[S16] H. Lee, X. Wu, X. Yang, L. Sun, *ChemSusChem* **2017**, 10, 4690.

[S17] D. V. Shinde, L. D. Trizio, Z. Dang, M. Prato, R. Gaspari, L. Manna, *Chem. Mater.* **2017**, *29*, 7032.

[S18] I. Mondal, J. N. Hausmann, G. Vijaykumar, S. Mebs, H. Dau, M. Driess, P. W. Menezes, *Adv. Energy Mater.* **2022**, *12*, 2200269.

[S19] P. W. Menezes, C. Panda, S. Garai, C. Walter, A. Guiet, M. Driess, *Angew. Chem. Int. Ed.* **2018**, *130*, 15457.

[S20] C. Walter, P. W. Menezes, S. Orthmann, J. Schuch, P. Connor, B. Kaiser, M. Lerch, M. Driess, *Angew. Chem., Int. Ed.* **2018**, *130*, 706.

[S21] M. L. Lindstrom, R. Gakhar, K. Raja, D. Chidambaram, *J. Electrochem. Soc.* **2020**, *167*, 046507.

[S22] H. Y. Wang, Y. Y. Hsu, R. Chen, T. S. Chan, H. M. Chen, B. Liu, *Adv. Energy Mater.* **2015**, *5*, 1500091.

[S23] X. Zhang, B. Zhang, S. Liu, H. Kang, W. Kong, S. Zhang, Y. Shen, B. Yang, *Appl. Surf. Sci.* **2018**, *436*, 974.

[S24] A. Han, H. Chen, Z. Sun, J. Xu, P. Du, *Chem. Commun.* **2015**, *51*, 11626.

[S25] P. Tian, Y. Yu, X. Yin, X. Wang, *Nanoscale* **2018**, *10*, 5054.

[S26] X. Wang, B. Li, Y.-P. Wu, A. Tsamis, H.-G. Yu, S. Liu, J. Zhao, Y.-S. Li, D.-S. Li, *Inorg. Chem.* **2020**, *59*, 4764.

[S27] Y. K. Li, G. Zhang, W. T. Lu, F. F. Cao, *Adv. Sci.* **2020**, *7*, 1902034.

[S28] P. W. Menezes, C. Walter, J. N. Hausmann, R. Beltrán-Suito, C. Schlesiger, S. Praetz, V. Yu. Verchenko, A. V. Shevelkov, M. Driess, *Angew. Chem. Int. Ed.* **2019**, *58*, 16569−16574.

[S29] Y. Jin, X. Yue, C. Shu, S. Huang, P. K. Shen, *J. Mater. Chem. A* **2017**, *5*, 2508−2513.

[S30] F. Qin, Z. Zhao, M. K. Alam, Y. Ni, F. Robles-Hernandez, L. Yu, S. Chen, Z. Ren, Z. Wang, J. Bao, *ACS Energy Lett.* **2018**, *3*, 546−554.

[S31] Z. Chen, H. Yang, S. Mebs, H. Dau, M. Driess, Z. Wang, Z. Kang, P. W. Menezes, *Adv. Mater.* **2023**, *35*, 2208337.

[S32] G. Qian, J. Chen, T. Yu, L. Luo, S. Yin, *Nano-micro Lett.* **2021**, *13*, 1−13.

[S33] B. Zhang, X. Zhang, Y. Wei, L. Xia, C. Pi, H. Song, Y. Zheng, B. Gao, J. Fu, P. K. Chu, *J. Alloys Compd.* **2019**, *797*, 1216−1223.

[S34] Y. Wu, Y. Gao, H. He, P. Zhang, *Electrochim. Acta* **2019**, *301*, 39-46.

[S35] Q. Tan, T. Xiong, F. Yang, P. Huang, D. Adekoya, Y. Huang, M.-S. Balogun, *J. Alloys Compd.* **2021**, *858*, 157729.

[S36] Y. Huang, F. Pei, G. Ma, Z. Ye, X. Peng, D. Li, Z. Jin, *ACS Appl. Mater. Interfaces* **2022,** *14*, 784−793.

[S37] A. Sivanantham, P. Ganesan, S. Shanmugam, *Adv. Funct. Mater.* **2016**, *26*, 4661−4672.

[S38] H. Sun, Y. Lian, C. Yang, L. Xiong, P. Qi, Q. Mu, X. Zhao, J. Guo, Z. Deng, Y. Peng, *Energy Environ. Sci.* **2018**, *11*, 2363−2371.

[S39] R. A. Marquez-Montes, K. Kawashima, Y. J. Son, J. A. Weeks, H. H. Sun, H. Celio, V. H. Ramos-Sanchez, C. B. Mullins, *J. Mater. Chem. A* **2021**, *9*, 7736−7749.

[S40] A. Peugeot, C. E. Creissen, D. Karapinar, H. N. Tran, M. Schreiber, M. Fontecave, *Joule* **2021**, *5*, 1281−1300.

[S41] D. Li, Y. Qin, J. Liu, H. Zhao, Z. Sun, G. Chen, D. Y. Wu, Y. Su, S. Ding, C. Xiao, *Adv. Funct. Mater.* **2022**, *32*, 2107056.

[S42] Y. Wang, X. P. Li, M. M. Zhang, Y. G. Zhou, D. W. Rao, C. Zhong, J. F. Zhang, X. P. Han, W. B. Hu, Y. C. Zhang, K. Zaghib, W. S. Wang, Y. D. Deng, *Adv. Mater*. **2020**, *32*, 2000231.

[S43] L.-L. Feng, G. Yu, Y. Wu, G.-D. Li, H. Li, Y. Sun, T. Asefa, W. Chen, X. Zou, *J. Am. Chem. Soc.* **2015**, *137*, 14023−14026.

[S44] W. Zhu, G. Zhu, J. Hu, Y. Zhu, H. Chen, C. Yao, Z. Pi, S. Zhu, E. Li, *Inorg. Chem. Commun.* **2020**, *114*, 107851.

[S45] S. Liang, B. Wei, M. Yuan, Y. Li, X. Ma, Y. Wu, L. Xu, *ChemistrySelect* **2020**, *5*, 3062−3068.
